# Pseudomolecule-level assembly of the Chinese oil tree yellowhorn (*Xanthoceras sorbifolium*) genome

**DOI:** 10.1093/gigascience/giz070

**Published:** 2019-06-26

**Authors:** Quanxin Bi, Yang Zhao, Wei Du, Ying Lu, Lang Gui, Zhimin Zheng, Haiyan Yu, Yifan Cui, Zhi Liu, Tianpeng Cui, Deshi Cui, Xiaojuan Liu, Yingchao Li, Siqi Fan, Xiaoyu Hu, Guanghui Fu, Jian Ding, Chengjiang Ruan, Libing Wang

**Affiliations:** 1 State Key Laboratory of Tree Genetics and Breeding, Research Institute of Forestry, Chinese Academy of Forestry, Beijing 100091, China; 2 Key Laboratory of Biotechnology and Bioresources Utilization, State Ethnic Affairs Commission & Ministry of Education, Dalian Minzu University, Dalian 116600, China; 3 National Demonstration Center for Experimental Fisheries Science Education, Key Laboratory of Exploration and Utilization of Aquatic Genetic Resources (Ministry of Education) and International Research Center for Marine Biosciences (Ministry of Science and Technology), Shanghai Ocean University, Shanghai 201306, China; 4 State Key Laboratory of Tree Genetics and Breeding, Northeast Forestry University, Harbin 150040, China; 5 Key Laboratory of Saline-alkali Vegetation Ecology Restoration (SAVER), Ministry of Education, Alkali Soil Natural Environmental Science Center (ASNESC), Northeast Forestry University, Harbin 150040, China; 6 Beijing ABT Biotechnology Co., Ltd., Beijing 102200, China; 7 Zhangwu Deya yellowhorn Professional Cooperatives, Zhangwu 123200, China

**Keywords:** *Xanthoceras sorbifolium*, yellowhorn, PacBio sequencing, genome assembly, Hi-C, genome annotation, conserved chromosome

## Abstract

**Background:**

Yellowhorn (*Xanthoceras sorbifolium*) is a species of the Sapindaceae family native to China and is an oil tree that can withstand cold and drought conditions. A pseudomolecule-level genome assembly for this species will not only contribute to understanding the evolution of its genes and chromosomes but also bring yellowhorn breeding into the genomic era.

**Findings:**

Here, we generated 15 pseudomolecules of yellowhorn chromosomes, on which 97.04% of scaffolds were anchored, using the combined Illumina HiSeq, Pacific Biosciences Sequel, and Hi-C technologies. The length of the final yellowhorn genome assembly was 504.2 Mb with a contig N50 size of 1.04 Mb and a scaffold N50 size of 32.17 Mb. Genome annotation revealed that 68.67% of the yellowhorn genome was composed of repetitive elements. Gene modelling predicted 24,672 protein-coding genes. By comparing orthologous genes, the divergence time of yellowhorn and its close sister species longan (*Dimocarpus longan*) was estimated at ∼33.07 million years ago. Gene cluster and chromosome synteny analysis demonstrated that the yellowhorn genome shared a conserved genome structure with its ancestor in some chromosomes.

**Conclusions:**

This genome assembly represents a high-quality reference genome for yellowhorn. Integrated genome annotations provide a valuable dataset for genetic and molecular research in this species. We did not detect whole-genome duplication in the genome. The yellowhorn genome carries syntenic blocks from ancient chromosomes. These data sources will enable this genome to serve as an initial platform for breeding better yellowhorn cultivars.

## Data Description

### Background

**Figure 1:**
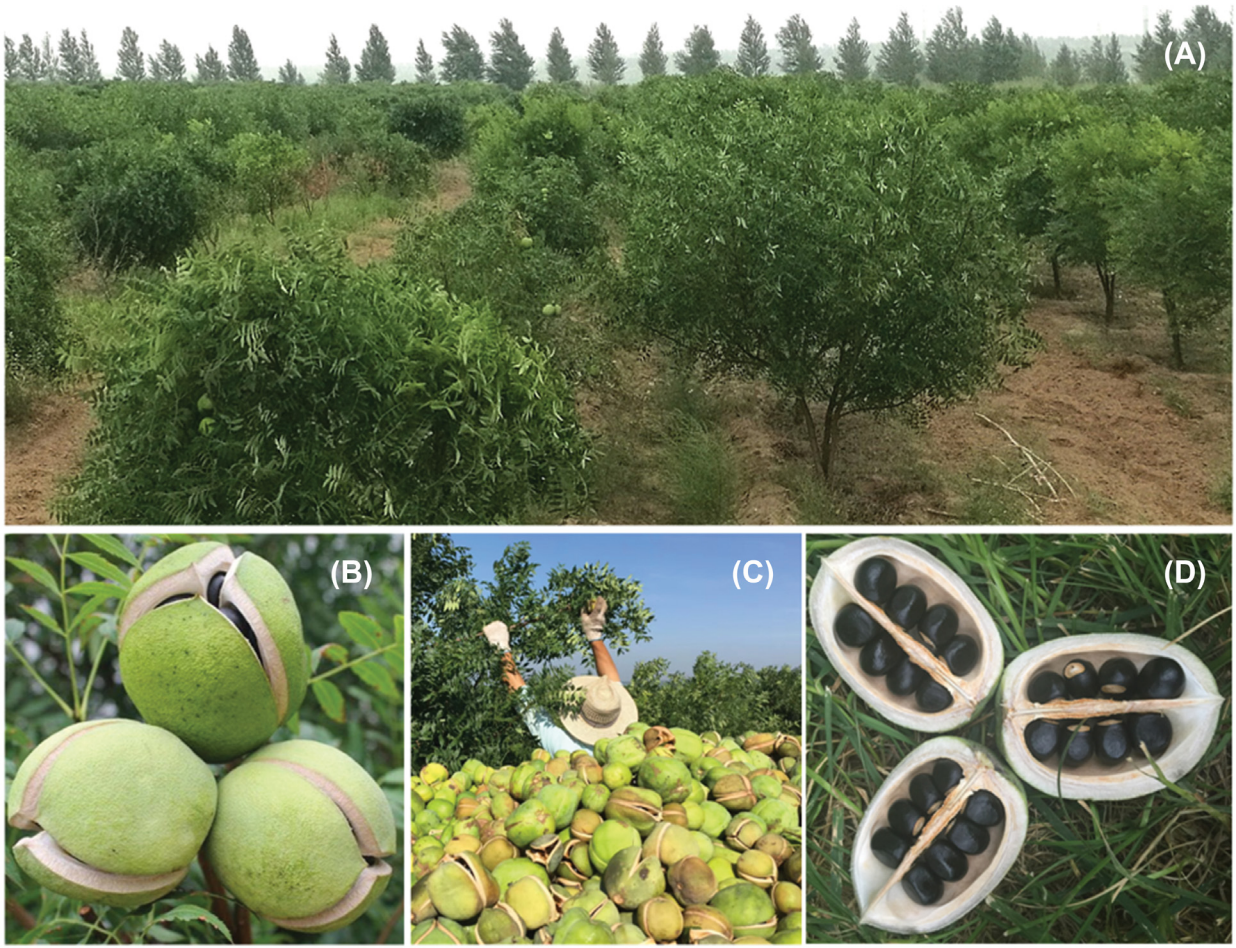
Images of yellowhorn plants. (**A**) Yellowhorn tree in an artificial forest. (**B**) Ripe fruit, which dehisce into 3 parts by the carpels. (**C**) A harvest scene of yellowhorn in northern China. (**D**) Seeds in ripe fruits, which number 18–24 in 1 fruit.

Yellowhorn (*Xanthoceras sorbifolium*) (NCBI: txid99658) is a woody oil species [1] that belongs to the Sapindaceae family and the monotypic genus *Xanthoceras*. As an endemic and economically important species in northern China, it is widely used for soil and water conservation owing to its capacity to survive on arid, saline, and alkaline land and in extreme temperatures even below −40°C [2, 3]. Almost 7.5 × 10^5^ tons of yellowhorn seeds are harvested in autumn every year [4] (Fig. 1). The oil content of its seed kernels can be as high as 67%, of which 85–93% is unsaturated fatty acid, including 37.1–46.2% linoleic acid and 28.6–37.1% oleic acid, which are essential fatty acids in the human diet [5]. Recently, as a major woody oil plant species, yellowhorn has drawn governmental and popular attention because of the shortage of vegetable oil resources in China. Notably, an essential nutrient for brain growth and maintenance—nervonic acid, which is rarely found in plants—accounts for 3.04% of the seed oil of yellowhorn [6, 7]. Recent results indicate that xanthoceraside, a novel triterpenoid saponin extracted from yellowhorn husks, has an antitumor effect and the potential to treat Alzheimer disease [8–10]. In this study, we generated a high-quality yellowhorn genome assembly and conducted annotation and genomic structure and evolution analyses. Our data provide a rich resource of genetic information for developing yellowhorn resources and understanding the special place of *Xanthoceras* and Sapindaceae in plant evolution.

### Sequenced individual and sample collection

Tender leaves were collected from an individual of *X. sorbifolium* cv. Zhongshi 4, which is a new variety issued by the National Forestry and Grassland Administration (variety rights No. 20180121), in Zhangwu, Liaoning, China. This tree was produced via clone of a plus tree from natural population in Tongliao, Inner Mongolia, China. The leaves were frozen in liquid nitrogen and stored at −80°C until DNA extraction.

### Estimation of genome size through flow cytometry analysis

**Figure 2:**
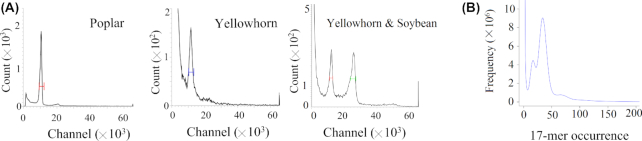
Estimation of genome size. (**A**) Test results of yellowhorn, poplar, and yellowhorn + soybean samples using flow cytometry. (**B**) Distribution of 17-mer frequency. The *x*-axis and *y*-axis indicate the 17-mer frequency and number, respectively. The leftmost truncated peak at a low occurrence frequency (1–2) was mainly due to random base errors in the raw sequencing reads.

One-month-old leaves from the sequenced yellowhorn individual were subjected to flow cytometry analysis to estimate the genome size as described by Galbraith et al. [11]. *Glycine max* var. William 82 (2C genome size = 2.28 pg) [12, 13] and *Populus trichocarpa* var. Nisqually 1 (2C genome size = 0.99 pg) [14] were used as standard references. The soybean and yellowhorn samples were chopped together using a razor blade and the nuclei were stained with propidium iodide. To avoid peaks that were too close to be distinguished when run simultaneously, the poplar and yellowhorn samples were run separately. Each sample was measured 3 times on the flow cytometer. More than 3,000 nuclei were analysed per sample with a FACSAria flow cytometer (Becton, Dickinson and Company, Franklin Lakes,NJ, USA). A total of 16 samples were analysed using soybean and poplar as standard species. The software BD FACSDiva (version 8.0.1) was used for data analysis with the coefficient of variation controlled at 5%. Compared with the soybean internal standard (peak at 25,413) and poplar reference (peak at 10,363), the peak fluorescence intensity values of yellowhorn samples were 11,968 and 11,558, respectively. With the soybean genome size (1,115 Mb) and poplar genome size (485 ± 10 Mb) [13–15] taken as reference, the yellowhorn genome size was estimated to be ∼525.94 and 540.93 Mb, which were relatively close (Fig. 2A).

### Illumina short-read sequencing and heterozygosity analysis

DNA was extracted from the leaves of the same individual using a DNA Secure Plant Kit (Tiangen, China). The DNA concentration and quality were assessed by 1% agarose gel electrophoresis and with a 2.0 Fluorometer (Life Technologies, CA, USA). One shotgun library with an insert size of 350 bp was prepared using a NEB Next® Ultra DNA Library Prep Kit (NEB, USA). A total of 34.51 Gb raw sequencing data were generated by the Illumina HiSeq X Ten sequencing platform. Primary data analysis was carried out using the standard Illumina pipeline [16]. Short reads were processed with Trimmomatic version 0.33 (Trimmomatic, RRID:SCR_011848) [17, 18] and Cutadapt (version 1.13) [19] to remove adapters, leading and trailing bases with a quality score <20, and reads with an average per-base quality of 20 over a 4-bp sliding window. Trimmed reads <70 nucleotides long were discarded. Finally, 34.40-Gb clean reads were used for the following analysis and error correction of Pacific Biosciences (PacBio) reads.

A *k*-mer analysis was performed to estimate the genomic characteristics as mentioned by Marçais and Kingsford [20]. After low-quality, duplicate, and contaminating reads were filtered out from 34.4 Gb Illumina sequencing data, 21.17-Gb high-quality clean reads were used to generate a *k*-mer (*k* = 17) depth distribution curve using Jellyfish (v2.1.1) (with the parameters -m 17 -t 10 -s 550M) and GCE v1.0.0 [21]. The frequency of 17-mer occurrence (17-mer depth) and the frequency of those 17-mers’ species at a given sequencing depth were counted and drawn distribution curves of *k*-mer frequency (Fig. 2B). On the basis of the flow cytometry results and computational method [21, 22], the middle peak (∼34×) was homogzygous peak. The left peak of 17× was heterozygous peak, and the right tiny peak (66×) observed in Fig. 2B was caused by repeat sequences. Depending on the formula reported by Liu et al. [21], the heterozygosity was estimated at ∼0.75%.

### PacBio SMRT sequencing

Genomic DNA was extracted following the ∼40 kb SMRTbell™ Libraries Protocol [23]. The DNA was purified with a Mobio PowerClean® Pro DNA Clean-Up Kit, and its quality was assessed by standard agarose gel electrophoresis and Thermo Fisher Scientific Qubit Fluorometry. The genomic DNA was sheared to a size range of ∼40 kb using g-TUBE (Covaris) and 0.45× AMPure beads were used to enrich and purify large fragments of DNA. Damaged DNA and ends were enzymatically repaired as recommended by PacBio. Following this procedure, hairpin adapters were ligated using a blunt-end ligation reaction. The remaining damaged DNA fragments and fragments without adapters at both ends were digested using exonuclease. Subsequently, the resulting SMRTbell templates were purified by Blue Pippin electrophoresis (Sage Sciences) and sequenced on a PacBio RS II instrument using P6-C4 sequencing chemistry. A primary filtering analysis was performed on the sequencer, and the secondary analysis was performed utilizing the Single-Molecule Real-Time (SMRT) analysis pipeline version 2.1.0 (PacBio). In total, we generated 66.44 Gb (∼122.83-fold coverage of the yellowhorn genome) of single-molecule sequencing data (6,105,692 PacBio post-filtered reads), with a mean read length of 10,882 bp (Fig. S1; Table S1).

### Genome assembly

After stringent filtering and correction steps using *k*-mer frequency-based methods [24], we assembled contigs using the PacBio reads. Preliminary assembly with the assembler Falcon v0.7 (Falcon, RRID:SCR_016089) [25] (falcon_sense_option = –output_multi –min_idt 0.70 –min_cov 4 –max_n_read 300 –n_core 8 overlap_filtering_setting = –max_diff 100 –max_cov 100 –min_cov 2 –n_core 12 –bestn 10) generated a total length of 598.65 Mb of contigs with an N50 length of 1.11 Mb, using the 66.44-Gb PacBio long reads. The software Quiver (based on pbsmrtpipe.pipelines.sa3_ds_resequencing in smrtlink_5.0.1; [26]) was used to polish the PacBio consensus sequence clusters. The assembly was corrected with Pilon version 1.22 (Pilon, RRID:SCR_014731) [27] using the Illumina short reads. Finally, heterozygous sequences were identified and removed using the Purge Haplotigs pipeline, with the parameters -a 75 [28, 29]. Contigs from organelle DNA sources can also be identified and filtered out during the processing with Purge Haplotigs. After the heterozygous sequences were removed, a final assembly from the PacBio reads (504.20 Mb) was generated (Table 1).

**Table 1:** Overview of assembly and annotation for the yellowhorn genome

Total length	504,196,643 bp
Length of unclosed gaps	73,800 bp
N50 length	
Initial contigs	1,044,891 bp
Scaffolds	32,173,403 bp
N90 length (scaffolds)	25,069,408 bp
No. of scaffolds (>N90 length)	21
Largest scaffold	40,097,451 bp
Guanine-cytosine content	36.95%
No. predicted	
Protein-coding genes	24,672
Noncoding RNA genes	1,066
Content of repetitive sequences	68.67%
Length of genome anchored on linkage groups	489,286,946 bp (97.04%)

### Pseudomolecule construction and 3D chromatin conformation analysis

The Hi-C technology is an efficient strategy for pseudomolecule construction and enables the generation of genome-wide 3D chromosome architectures. We constructed Hi-C fragment libraries of 350 bp and sequenced them using the Illumina Hi-Seq platform (Illumina, San Diego, CA, USA) for chromosome pseudomolecule construction. Mapping of the Hi-C reads and assignment to restriction fragments were performed as described by Burton et al. [30]. A total of 53.39 Gb of trimmed reads, representing ∼98.70-fold coverage of the yellowhorn genome, were mapped to the assembly with the aligner BWA version 0.7.10 (BWA, RRID:SCR_010910; parameters: bwa index -a bwtsw fasta bwa aln -M 3 -O 11 -E 4 -t 2 fq1 bwa aln -M 3 -O 11 -E 4 -t 2 fq2) [31]. Only uniquely aligned reads with high alignment quality (>20) were selected for pseudomolecule construction. Duplicate removal and quality assessment were performed using HiC-Pro (version 2.8.1) with the following parameters: mapped_2hic_fragments.py -v -S -s 100 -l 1000 -a -f -r -o [32]. In total, 50.56% of the Hi-C data were grouped into valid interaction pairs. A total of 2,836 contigs (N50 length at 1.04 Mb) were assembled after error correction. LACHESIS (parameters: cluster_min_re_sites = 48; cluster_max_link_density = 2; cluster_noninformative_ratio = 2; order_min_n_res_in_trun = 14; order_min_n_res_in_shreds = 15) [30] was used to assign the order and orientation of each group, with a scaffold N50 of 32.17 Mb.

Using the 98.70-fold coverage of Hi-C reads, 489.28 Mb (97.04%) of the assembly were anchored onto the 15 pseudomolecules, which were in agreement with the yellowhorn karyotype (2n = 30) identified by Li [33]. The assembly (477.59 Mb, 94.76%) was ordered by the frequency distribution of valid interaction pairs (Table 2, Fig. S2). The coverage of the assembly reached 93.96% and the ratio of unclosed gaps was 0.15‰ (Table 1). The assembly was of sufficient quality to be used as a reference for studying yellowhorn biology and plant genomics.

**Table 2:** Quantity of the contigs anchored with Hi-C

Linkage Group	No. of anchored contigs	Sequence length (bp)
1	68	40,738,791
2	92	40,039,835
3	38	37,159,809
4	112	35,552,403
5	84	35,291,867
6	62	35,706,508
7	66	33,002,525
8	46	32,947,898
9	66	30,804,552
10	62	30,699,318
11	68	29,306,026
12	56	29,390,540
13	47	29,816,145
14	71	25,601,946
15	72	23,228,783
Total (ratio %)	1,010 (35.61)	489,286,946 (97.04)

### Transcriptome sequencing

RNA was extracted from 3 tissues (flowers, leaves, and roots) of the same individual used for DNA sequencing using the Easy Spin RNA extraction kit (Sangon Biotech, Shanghai, China; No. SK8631). The concentration of each RNA sample was checked using a NanoDrop spectrophotometer (Thermo Fisher Scientific Inc., USA) and a QUBIT® Fluorometer (Life Technologies). The RNA integrity was checked using a Bioanalyzer 2100 (Agilent Technologies, Santa Clara, CA). Iso-Seq libraries were prepared according to the Isoform Sequencing protocol (Iso-Seq) using the Clontech SMARTer PCR cDNA Synthesis Kit and the BluePippin Size Selection System protocol as described by PacBio (PN 100–092-800–03). A mixed sample was sequenced on the PacBio RS II platform using P6-C4 chemistry.

The sequence data were processed using the SMRTlink 4.0 software. Circular consensus sequences were derived from the subread BAM files with the following parameters: min_length 200, max_drop_fraction 0.8, no_polish TRUE, min_zscore -999, min_passes 1, min_predicted_accuracy 0.8, max_length 18 000. Separation of the full-length and non−full-length reads was conducted using pbclassify.py (ignorepolyA false, minSeqLength 200). The non−full-length and full-length fasta files produced were then fed into the cluster step to cluster the isoforms, and subjected to final Arrow polishing with the parameters hq_quiver_min_accuracy 0.99, bin_by_primer false, bin_size_kb 1, qv_trim_5p 100, qv_trim_3p 30. The LoRDEC software (version 0.3) was used to correct sequencing errors in the consensus transcripts using the Illumina reads as a reference (parameters: -k 19 -s 3) [34]. The corrected consensus transcripts were clustered using CD-HIT (version 4.6.8) (-c 0.99 -T 6 -G 0 -aL 0.90 -AL 100 -aS 0.99 -AS 30) [35] to reduce sequence redundancy and improve the performance of other sequence analyses.

A total of 110,584 non-redundant unigenes were generated from 142,396 transcripts in the final RNA assemblies, which were used as evidence to assist with gene prediction. Among the 110,584 non-redundant transcripts, 8,466 (7.66%) were non-coding messenger RNAs (mRNAs). Each gene had an average of 2–7 transcripts, among which the longest transcript representing that gene was kept in the final gene model set.

### Evaluation of assembly quality

The completeness of the final assembly was evaluated using CEGMA version 2.5 (CEGMA, RRID:SCR_015055) [36, 37] and BUSCO version 3.0.2 (BUSCO, RRID:SCR_015008) [38–40]. The CEGMA outputs showed that 94.76% of the core eukaryotic genes (235 of 248 core eukaryotic genes) were present in our assembly. The BUSCO test, referencing the embryophyta protein set (run_BUSCO.py -i plant_species.fa -o plant_species-l embryophyta_odb9/-m proteins), identified 94.7% of plant gene sets as complete (1,364 of 1,440 BUSCOs), including 89.0% single-copy and 5.7% duplicated genes (Table S2). All of these results suggested a high assembly quality for the yellowhorn genome.

### Annotation of repetitive sequences

A *de novo* repeat database was constructed using RepeatScout version 1.0.5 (RepeatScout, RRID:SCR_014653) [41], LTR-FINDER (version 1.0.7) [42], MITE-Hunter (version 1.0) [43], and PILER (version 1.0) with default parameters [44]. The predicted repeats were classified using PASTEClassifier (version 1.0) with default parameters [45, 46]. Then, RepeatMasker version 4.0.7 (RepeatMasker, RRID:SCR_012954) [47] was used with the following parameters: “-nolow -no_is -norna -engine wublast -qq -frag 20 000” to identify repeat sequences by aligning them against known gene and genome sequences, based on Repbase (version 19.06) [48] and the *de novo* repeat database.

**Figure 3:**
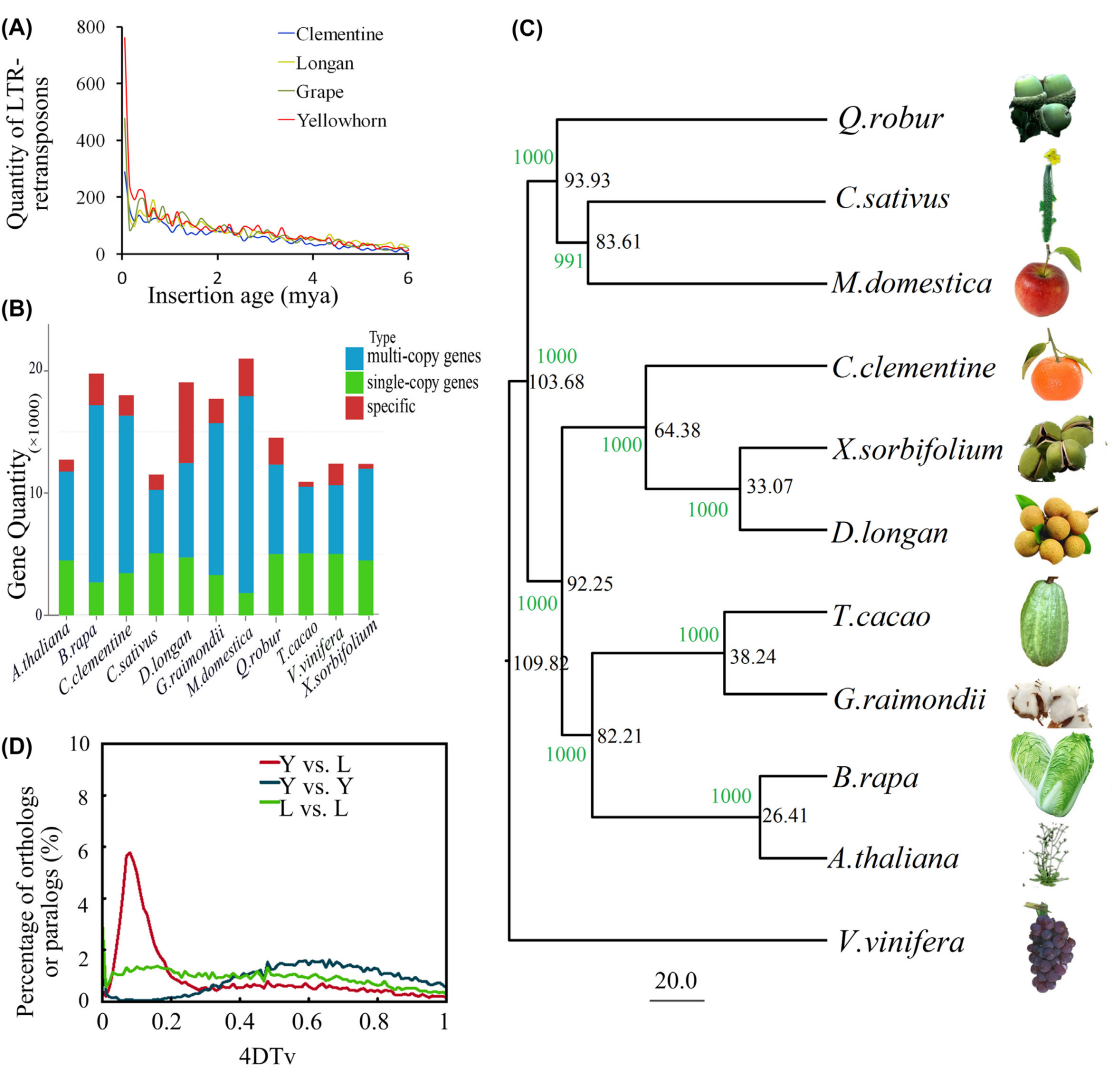
Genome evolution. (**A**) Distribution of insertion ages of LTR-retrotransposons. The *x*-axis represents the estimated insertion age (mya) of the LTR-retrotransposons. The *y*-axis represents the number of intact LTR-retrotransposons. (**B**) Comparison of copy numbers in gene clusters of analysed eudicot genomes. According to the identified gene clusters, the genes were grouped into single-copy, multiple-copy, and species-specific (specific) genes. (**C**) Constructed phylogenetic tree and divergence time estimation. The black numbers represent estimated divergence times (mya), which are measured with a scale bar of 20 million years, and green numbers represent bootstrap values. Grape (*V. vinifera*) was used as an outgroup. (**D**) Genome duplication in eudicot genomes as revealed through 4DTv analyses. The percentages of the orthologous pairs (Y vs L) between yellowhorn (Y) and longan (L) and paralogous gene pairs within the yellowhorn (Y vs Y) and longan (L vs L) genomes are plotted against their calculated 4DTv values.

The predicted repeats represented 346.39 Mb (68.67%) of the yellowhorn genome assembly. Among these repeats, 2 types of long terminal repeat (LTR)-retrotransposons were the most abundant, including 98.68 Mb of *Copia*-type (19.57%) and 88.24 Mb of *Gypsy*-type (17.50%) repeats (Table S3). Accumulation of LTR-retrotransposons is an important contributor to genome expansion and diversity [49]. The insertion time of LTR-retrotransposons in the genome was estimated by calculating the sequence variance between the LTR arms of each LTR-retrotransposon, using a substitution rate of 1.3 × 10^−8^ substitutions per site per year [50]. To calculate the insertion age of each LTR retrotransposon, the 5′ and 3′ LTRs of each element were aligned with MUSCLE version 3.8.31 (MUSCLE, RRID:SCR_011812) using default setting parameters [51, 52]. Distmat (with default parameters) was used to estimate the DNA divergence between the LTR sequences with the Kimura-2-parameter base substitution model [53] and DNA divergence was converted to divergence time. A comparison of the insertion ages for LTR-retrotransposons showed similar insertion profiles among the genomes of clementine [54] (annotation version 1.0), longan [55] (annotation version 1.0), grape [56] (*Vitis vinifera*, annotation version GenomeScope.12X), and yellowhorn (Fig. 3A). We observed that the yellowhorn genome carried more young LTR-retrotransposons, with the highest proportion of LTR-retrotransposons with insertion ages <0.2 million years ago (mya). This might have resulted from rapid changes of its growing environment, such as the effects of pathogens and interference from human activities in recent years. The genomes sequenced by pure next-generation sequencing technology might show fewer LTR-retrotransposons because the sequence similarity between LTR arms and among different LTR-retrotransposons probably caused assembly errors in these regions, which may have led to underestimation of the number of LTR-retrotransposons in clementine and longan. Comparison of the insertion ages suggested a similar insertion age between *Copia*-type and *Gypsy*-type LTR-retrotransposons (Fig. S3).

### Prediction of protein-coding genes

Annotation of protein-coding genes in the yellowhorn genome was conducted by combining *de novo* prediction, homology information, and RNA-seq data. For the *de novo* prediction, Genscan (version 3.1) [57], Augustus (Augustus: Gene Prediction, RRID:SCR_008417) (version 3.1) [58], GlimmerHMM version 3.0.4 (GlimmerHMM, RRID:SCR_002654) [59], GeneID (version 1.4) [60], and SNAP (version 2006–07-28) [61] were used to analyse the repeat-masked genome with default parameters. For the similarity-based prediction, the Uniprot protein sequences from 3 sequenced plants, Arabidopsis (The Arabidopsis Information Resource [TAIR] 10, [62]), longan (V1.0, [63]), and grape (Genomescope 12×, [64]), were aligned against the *ab initio* gene models in the yellowhorn genome using GeMoMa (version 1.3.1) [65]. When multiple transcripts were predicted at the same location, the highest GeMoMa scoring transcript was chosen as the optimal model [66]. The RNA-seq data were aligned to the reference genome with PASA (version 2.0.2) [67] under default parameters. All predictions from the 3 methods were combined with EVidenceModeler (v1.1.1) (Mode: STANDARD S-ratio: 1.13 score>1000) [68] to produce a consensus gene set. During the EVM integration, higher weights were assigned to the predicted PASA and GeMoMa models than the *ab initio* models. PASA was used to modify the final gene models.

The RNA-seq reads were then aligned to the yellowhorn genome assembly with TopHat (TopHat, RRID:SCR_013035) (v2.0.10, implemented with bowtie2) [69] to identify candidate exon regions and splicing donor and acceptor sites to evaluate the gene prediction results. Infernal version 1.1 (Infernal, RRID:SCR_011809) (default parameters) [70] was used to identify non-coding ribosomal RNA (rRNA) and microRNA genes based on Rfam (version 12.1) [71] and miRbase (version 21) [72]. TRNAscan-SE (version 1.3.1) (default parameters) [73] was used to identify transfer RNA (tRNA) genes.

GenBlastA v1.0.4(-e 1e-5) was used to perform pseudogene prediction by scanning the yellowhorn genome for sequences homologous to the known protein-coding genes it contained, and premature stop codons or frame shift mutations in those sequences were identified by GeneWise version 2.4.1 (GeneWise, RRID:SCR_015054) with the parameters -both -pseudo [74, 75].

Functional annotation of the protein-coding genes was carried out by searching against the NCBI non-redundant (nr), EuKaryotic Orthologous Groups (KOG), GO, KEGG, and TrEMBL databases. Additionally, the gene models were aligned to the Pfam database using Hmmer version 3.0 (Hmmer, RRID:SCR_005305) (parameters, -E 0.00001 –domE 0.00001 –cpu 2 –noali –acc) [75–81]. Gene Ontology (GO) terms were allocated to the genes using the Blast2GO version 2.2.31 (Blast2GO, RRID:SCR_005828) pipeline [81].

In total, we predicted 24,672 protein-coding genes (Table S4) and 1,913 pseudogenes, with a mean gene length of 4,199 bp, mean intron length of 2,560 bp, and mean coding sequence length of 1,580 bp. Of these genes, 99.02% (24,429) carried ≥1 conserved functional domain (Table S5). Their functions were classified using GO terms (Fig. S4) and the KOG database (Fig. S5). For the non-coding mRNA genes, 642 tRNA, 108 microRNA, and 316 rRNA genes were predicted in the yellowhorn genome.

### Chromosome synteny between the yellowhorn and reference genomes

**Figure 4:**
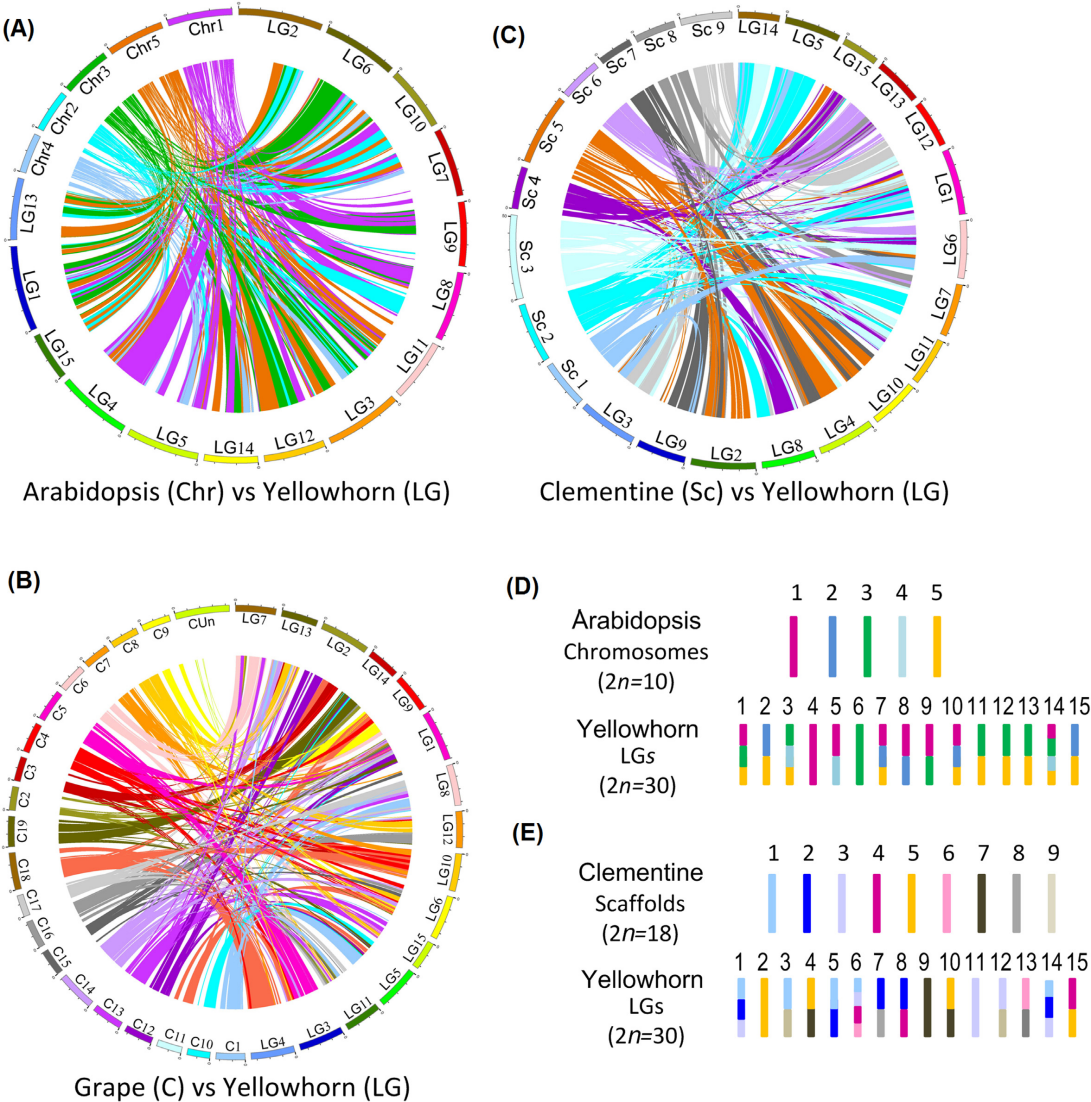
Chromosome synteny. The circularized blocks represent the chromosomes of yellowhorn and other genomes. Aligned genes identified by MCscanX are connected by lines, with their chromosome locations shown in different colours. (**A**) Chromosome alignment of yellowhorn and Arabidopsis. (**B**) Chromosome alignment of yellowhorn and grape. (**C**) Chromosome alignment of yellowhorn and clementine. Coloured ribbons connect the aligned genes. Yellowhorn linkage groups are labelled LG 1−15, Arabidopsis chromosomes labelled Chr 1−5, grape chromosomes are labelled C1−19 and CUn (chromosome location unknown), and clementine scaffolds are labelled Sc 1−9. Scale, 10 Mb. (**D**) Chromosome rearrangements between Arabidopsis and yellowhorn. (**E**) Chromosome rearrangements between clementine and yellowhorn. Arabidopsis and clementine chromosomes are represented as bars of different colours. Synteny and rearrangement of the yellowhorn chromosomes are indicated by different blocks, corresponding to the reference Arabidopsis and clementine chromosomes.

To investigate the evolution of the yellowhorn chromosomes, gene collinearity was determined by anchoring the aligned yellowhorn genes to the reference genomes of clementine, Arabidopsis, and grape using MCscan (version 0.8) [82]. The parameters of the MCscan alignment were as follows: $/MCScanX xxx.blast$-s 10 –b $2 (inter-species) blastp -query b.fa -db adb -out xyz.blast -evalue 1e-10 -num_threads 16 -outfmt 6 -num_alignments 5. A total of 367, 409, and 386 syntenic blocks were identified on the basis of the orthologous gene orders, corresponding to 28,372, 18,650, and 23,400 genes in each genome, respectively. The mean gene number per block was 77.3, 45.6, and 60.6 genes, respectively. This suggested that yellowhorn and clementine shared the highest collinearity, which was consistent with their close phylogenetic relationship as members of the Sapindales clade. The alignments of syntenic chromosomes were visualized between yellowhorn and the other genomes. The frequency of large-scale fragment rearrangements between yellowhorn and clementine, including inversions and translocations, was considerably lower than between yellowhorn and the other 2 genomes (Fig. 4). In particular, structural variation between yellowhorn and grape was so frequent that it was too difficult to speculate on the syntenic relationships among the chromosomes (Fig. 4B). The chromosome alignments between yellowhorn linkage groups (LGs) and clementine pseudomolecules revealed that most of the cross-chromosome rearrangements were different from those between yellowhorn and Arabidopsis (Fig.   4D and E). Yellowhorn LGs 2 and 11 were found to be syntenic to single clementine pseudomolecules, Scaffold 5 and 3, respectively, and LGs 3, 4, 5, 7, 8, 10, 12, 14, and 15 were each aligned to 2 reference chromosomes of clementine. Comparatively, frequency of chromosome rearrangement was a little higher between the yellowhorn LGs and Arabidopsis chromosomes. Arabidopsis Chromosome 1 was predominantly syntenic to yellowhorn LG 4, which demonstrated that the yellowhorn genome contained some conserved genome structure from its originals (Fig.   4D). Intriguingly, similar chromosomal fusion events were found among some chromosomes. Aligned fragments of Arabidopsis Chromosomes 1, 3, and 5 were fused to form yellowhorn LGs 1 and 14, similarly to clementine Scaffolds 1, 2, and 3. Yellowhorn LG 6 was aligned to clementine scaffolds 1, 3, 4, and 6 but had extensive collinearity with Arabidopsis Chromosome 3 (Fig.   4D, E). However, phylogenetic analysis suggested a distant relationship between Arabidopsis and yellowhorn. These findings suggested that Arabidopsis and yellowhorn share a chromosome of their origins, despite extensive rearrangements. Overall, these findings shed new light on the evolution of eudicot plant chromosomes.

### Identification of gene clusters and duplication

Gene clustering was conducted using OrthoMCL version 5 (OrthoMCL DB: Ortholog Groups of Protein Sequences, RRID:SCR_007839, parameters: Pep_length 10 Stop_coden 20 PercentMatchCutoff 50 EvalueExponentCutoff -5 Mcl 1.5 #1.2∼4.0) [83] among the protein sequences of 10 high-quality typical eudicot genomes representative of important families, including *D. longan* (Sapindaceae, Sapindales) [54], *Citrus clementina* (Rutaceae, Sapindales) [55], *Brassica rapa* (Brassicaceae, Brassicales), *Arabidopsis thaliana* (Brassicaceae, Brassicales) [84, 85], *Theobroma cacao* (Sterculiaceae, Malvales) [86], *Gossypium raimondii* (Malvaceae, Malvales) [87], *Quercus robur* (Fagaceae, Fagales) [88], *V. vinifera* (Vitaceae, Vitales) [56], *Cucumis sativus* (Cucurbitaceae, Cucurbitales) [89], and *Malus* × *domestica* (Rosaceae, Rosales) [90], as well as yellowhorn (Additional file: Table S6). The yellowhorn genes were clustered into a total of 14,828 families, including 169 yellowhorn-specific gene families (Additional file: Table S7). Comparison of gene copy numbers among the 11 eudicot genomes indicated that the yellowhorn genome had a similar proportion of single- and multiple-copy genes to the other analysed genomes (Fig. 3B). Intriguingly, the species-specific genes of yellowhorn were similar to those of *T. cacao*, which implied that the yellowhorn genes might have conserved the similar gene structure with their origins.

More than 300 one-to-one single-copy genes shared by all 11 genomes were identified and used to construct a phylogenetic tree using PhyML (version 3.0) (Fig. 3C) [91]. The TIM2+I+G model was used to construct the evolutionary tree as determined by jmodeltest. The software Muscle (version 3.8.31) [51, 52] was used to align the orthologs. The alignment outputs were treated with Gblocks (version 14.1) with the parameters -t = p -b5 = h -b4 = 5 -b3 = 15 -d = y -n = y [92]. Divergence times were estimated using MCMCtree (version 4.7a) [93, 94] with the following parameters: burn-in = 10 000, sample-number = 100 000, sample-frequency = 2. The TimeTree database [95], r8s (parameter: r8s -b -f r8s_in.txt > r8s_out.txt), and divergence time (Whelan and Goldman [96] and Yang et al. [97]) were used to calibrate the time. The fossil calibration times used in the evolutionary trees were as follows: (((Qrob, (Csat, Mdom)), ((Ccle, (Xsor, Dlon)), ((Tcac, Grai), (Brapa, Atha)“<30.9>20.4”))), Vvin)“<115>105”. The credibility intervals for the divergence time estimates were as follows: UTREE 1 = (((Qrob: 93.929608, (Csat: 83.608799, Mdom: 83.608799) [&95% = {67.268, 96.218}]: 10.320809) [&95% = {78.104, 105.034}]: 9.748170, ((Ccle: 64.380901, (Xsor: 33.069679, Dlon: 33.069679) [&95% = {18.376, 48.565}]: 31.311222) [&95% = {46.354, 81.164}]: 27.870851, ((Tcac: 38.243394, Grai: 38.243394) [&95% = {21.870, 56.407}]: 43.965024, (Brapa: 26.409279, Atha: 26.409279) [&95% = {20.721, 30.886}]: 55.799139) [&95% = {67.279, 94.364}]: 10.043334) [&95% = {77.382, 103.299}]: 11.426026) [&95% = {89.679, 113.000}]: 6.145826, Vvin: 109.823604) [&95% = {104.966, 114.982}]. Yellowhorn and longan in the Sapindaceae family showed the closest relationship, with the divergence time estimated at ∼33.07 mya. Using the orthologous gene pairs of yellowhorn and longan identified by gene collinearity and paralogous pairs identified by gene clustering, 4DTv (4-fold degenerate synonymous sites of the third codons) values were calculated for all of the duplicated pairs. A species divergence peak (4DTv ∼ 0.1) was observed in the yellowhorn vs longan ortholog 4DTv distribution, but no obvious peak could be seen in the yellowhorn and longan paralog curves (Fig. 3D). In a self-alignment of the chromosomes based on gene synteny, no large-scale gene duplications were found in the yellowhorn genome (Fig. S2), suggesting that the yellowhorn genome has not undergone whole-genome or large-fragment duplication.

## Availability of supporting data and materials

The raw sequence data have been deposited in NCBI under project accession No. PRJNA483857. The Biosample Number of transcriptome sequencing, Pacbio SMRT sequencing, Illumina short-read sequencing and Illumina sequencing for Hi-C were SAMN09748200, SAMN11653335, SAMN11653337 and SAMN11653336. The SRA accession No. of transcriptome sequencing, PacBio SMRT sequencing, Illumina short-read sequencing, and Illumina sequencing for Hi-C was SRR7768197, SRR7768198, SRR7768199, and SRR7768201, respectively (in SRP159119). The accession No. of *Xanthoceras sorbifolium* genome sequencing and assembly was QUWJ 00000000. All supplementary figures and tables are provided in Additional Files. Additional supporting data, including the genome assembly, annotations and phylogenetic tree files, are available via the GigaScience database GigaDB [98].

## Editor's Note

Please note, another Data Note presenting a genome assembly of *Xanthoceras sorbifolium* has been published back-to-back with this one in *GigaScience* [99].

## Additional Files

**Additional file 1:** Tables S1−S7

**Table S1:** PacBio data statistics.

**Table S2:** Genome quality assessed by the BUSCO test.

**Table S3:** Repetitive sequence content.

**Table S4:** Prediction of protein-coding genes.

**Table S5:** Function annotation of protein-coding genes.

**Table S6:** Data used in orthoMCL analysis.

**Table S7:** Annotation and locus information of 169 yellowhorn-specific gene families.

**Additional file 2:** Figures S1−S5

**Figure S1:** Length distribution of the 3 types of PacBio reads produced.

**Figure S2:** Interaction frequency distribution of Hi-C links among chromosomes.

**Figure S3**. Distribution of insertion ages of *Copia*-type and *Gypsy*-type LTR-retrotransposons.

**Figure S4:** Function classification of protein-coding genes against the GO term database.

**Figure S5:** KOG function classification of protein-coding genes.

giz070_GIGA-D-18-00337_Original_Submission

giz070_GIGA-D-18-00337_Revision_1

giz070_GIGA-D-18-00337_Revision_2

giz070_Response_to_Reviewer_Comments_Original_Submission

giz070_Response_to_Reviewer_Comments_Revision_1

giz070_Reviewer_1_Report_Original_Submission John Mackay, Ph.D. -- 10/28/2018 Reviewed

giz070_Reviewer_2_Report_Original_Submission Laura Kelly -- 11/11/2018 Reviewed

giz070_Reviewer_2_Report_Revision_1 Laura Kelly -- 4/18/2019 Reviewed

giz070_Reviewer_3_Report_Original_Submission Pedro Martinez Garcia -- 11/30/2018 Reviewed

giz070_Supplemental_Files

## Abbreviations

4DTv: 4-fold degenerate synonymous sites of the third codons; bp: base pair; BUSCO: Benchmarking Universal Single-Copy Orthologs; BWA: Burrows-Wheeler Aligner; CD-HIT: Cluster Database at High Identity with Tolerance; CEGMA: Core Eukaryotic Genes Mapping Approach; EVM: EVidenceModeler; Gb: gigabases; GCE: Genomic Character Estimator; GeMoMa: Gene Model Mapper; GO: Gene Ontology; kb: kilobases; KEGG: Kyoto Encyclopedia of Genes and Genomes; KOG: EuKaryotic Orthologous Groups; LG: linkage group; LTR: long terminal repeat; Mb: megabases; mRNA: messenger RNA; mya: million years ago; MCscan: Multiple Collinearity Scan toolkit; MITE: miniature inverted-repeat transposable element; MUSCLE: MUltiple Sequence Comparison by Log-Expectation; NCBI: National Center for Biotechnology Information; NR: non-redundant; PacBio: Pacific Biosciences; PE: paired-end; Pfam: Protein Families; PhyML: Phylogeny Maximum Likelihood; RNA-Seq: RNA sequencing; rRNA: ribosomal RNA; SMRT: Single-Molecule Real-Time; SNAP: Semi-HMM-based Nucleic Acid Parser; SRA: Sequence Read Archive; TAIR: The Arabidopsis Information Resource; tRNA: transfer RNA.

## Competing Interests

The authors declare that they have no competing interests.

## Funding

This work was financially supported by the Central Public-Interest Scientific Institution Basal Research Fund (CAFYBB2019QD001), the National “12th Five-Year” Plan for Science & Technology Support of China (2015BAD07B0106), the National Natural Science Foundation of China (31800571, 31870594, 31760213), the National Key Research and Development Plan of China (2016YFC050080506) and Key Research Development Program of Liaoning Province (2017204001).

## Authors' contributions

Q.B., H.Y., Y.Lu, C.R., and L.W. conceived and designed the study; T.C., X.L., Y.Li, S.F., X.H., G.F., Y.C., J.D., D.C., Z.Z., and Z.L. prepared materials and conducted the experiments; Q.B., Y.Z., W.D., Y.Lu, and L.G. wrote the manuscript.
