# Supplementary material for: Pseudomolecule-level assembly of the Chinese oil tree yellowhorn (Xanthoceras sorbifolium) genome
Source: Gigascience. 2019 Jun 26;8(6):giz070. doi: 10.1093/gigascience/giz070 (PMC6593361; doi:10.1093/gigascience/giz070)
Supplement: giz070_GIGA-D-18-00337_Original_Submission [file giz070_giga-d-18-00337_original_submission.pdf]

GigaScience

Reference genome of a Chinese yellowhorn Xanthoceras sorbifolium provides insights into its conservation of original chromosomes

--Manuscript Draft--

**Manuscript Number:**	GIGA-D-18-00337
**Full Title:**	Reference genome of a Chinese yellowhorn Xanthoceras sorbifolium provides insights into its conservation of original chromosomes
**Article Type:**	Data Note
**Funding Information:**	
**Abstract:**	Backgrounds: Yellowhorn (Xanthoceras sorbifolium) (NCBI Taxonomy ID: 99658) is a species of the Sapindaceae family in China. It is an oil tree that could sustain strictly cold and drought environments. As a tertiary legacy species, study of its genome will not only contribute to understand the evolution of genes and chromosomes, but also bring yellowhorn breeding into a genomic phase.<br>Findings: Here we generated 15 pseudomoleculars of the yellowhorn chromosomes, on which 97.04% of scaffolds anchored, using Illmina HiSeq, Pacifc Biosciences and Hi-C technologies. The final assembly genome of yellowhorn is 504.2 Mb with a contig N50 size of 1.04 Mb and a scaffold N50 size of 32.17 Mb. Genome annotation revealed that 68.67% of the yellowhorn genome is composed of repetitive elements. Gene modeling has predicted 24,672 protein-coding genes. Comparison of the identified orthologous genes estimated the divergence time of yellowhorn and its close sister species longan (Dimocarpus Longan) approximately at 38.79 mya. Gene clusters and chromosome synteny demonstrated that the yellowhorn genome conserved the genome structure of its ancestor in some chromosomes.<br>Conclusion: This genome assembly presented a high quality reference genome of yellowhorn. Integrated genome annotation provided a valuable data set for genetic and molecular research in this species. We did not detect the whole-genome duplication in this genome. The yellowhorn genome carried the fragment of its ancient chromosomes, reinforcing yellowhorn as a tertiary legacy species. All of these data sources will enable this genome to serve as an initial platform for breeding better yellowhorn.
**Corresponding Author:**	Libing Wang, Ph.D.<br><br>CHINA
**Corresponding Author Secondary Information:**	
**Corresponding Author's Institution:**	
**Corresponding Author's Secondary Institution:**	
**First Author:**	Quanxin Bi
**First Author Secondary Information:**	
**Order of Authors:**	Quanxin Bi
	Yang Zhao
	Wei Du
	Ying Lu
	Lang Gui
	Haiyan Yu
	Tianpeng Cui
	Deshi Cui
	Xiaojuan Liu

Powered by Editorial Manager® and ProduXion Manager® from Aries Systems Corporation

	Yingchao Li
	Siqi Fan
	Xiaoyu Hu
	Guanghui Fu
	Yifan Cui
	Jian Ding
	Chengjiang Ruan
	Libing Wang, Ph.D.
**Order of Authors Secondary Information:**	
**Additional Information:**	
**Question**	**Response**
Are you submitting this manuscript to a special series or article collection?	No
**Experimental design and statistics**<br><br>Full details of the experimental design and statistical methods used should be given in the Methods section, as detailed in our Minimum Standards Reporting Checklist. Information essential to interpreting the data presented should be made available in the figure legends.<br><br>Have you included all the information requested in your manuscript?	Yes
**Resources**<br><br>A description of all resources used, including antibodies, cell lines, animals and software tools, with enough information to allow them to be uniquely identified, should be included in the Methods section. Authors are strongly encouraged to cite Research Resource Identifiers (RRIDs) for antibodies, model organisms and tools, where possible.<br><br>Have you included the information requested as detailed in our Minimum Standards Reporting Checklist?	Yes
**Availability of data and materials**	Yes

All datasets and code on which the conclusions of the paper rely must be either included in your submission or deposited in publicly available repositories (where available and ethically appropriate), referencing such data using a unique identifier in the references and in the "Availability of Data and Materials" section of your manuscript.<br><br>Have you have met the above requirement as detailed in our Minimum Standards Reporting Checklist?	

Powered by Editorial Manager® and ProduXion Manager® from Aries Systems Corporation

Manuscript

Click here to access/download;Manuscript;MS_yellowhorn_maintext.pdf

Click here to view linked References

Reference genome of a Chinese yellowhorn *Xanthoceras sorbifolium* provides insights into its conservation of original chromosomes

Quanxin Bi[1,2,†], Yang Zhao[1,†], Wei Du[2,†], Ying Lu[3], Lang Gui[3], Haiyan Yu[1,4], Tianpeng Cui[5], Deshi Cui[5], Xiaojuan Liu[1], Yingchao Li[1], Siqi Fan[1], Xiaoyu Hu[1], Guanghui Fu[1], Yifan Cui[1], Jian Ding[2], Chengjiang Ruan[2,*], Libing Wang[1,*]

[1] State Key Laboratory of Tree Genetics and Breeding, Research Institute of Forestry, Chinese Academy of Forestry, Beijing 100091, China.

[2] Key Laboratory of Biotechnology and Bioresources Utilization, State Ethnic Affairs Commission & Ministry of Education, Dalian Minzu University, Dalian 116600, China.

[3] National Demonstration Center for Experimental Fisheries Science Education, Key Laboratory of Exploration and Utilization of Aquatic Genetic Resources (Ministry of Education) and International Research Center for Marine Biosciences (Ministry of Science and Technology), Shanghai Ocean University, Shanghai 201306, China.

[4] Beijing ABT Biotechnology Co., Ltd., Beijing 102200, China.

[5] Zhangwu Deya yellowhorn Professional Cooperatives, Zhangwu 123200, China.

*Correspondence address: Libing Wang, State Key Laboratory of Tree Genetics and Breeding, Research Institute of Forestry, Chinese Academy of Forestry, Beijing 100091, China; E-mail: wlibing@caf.ac.cn; Chengjiang Ruan, Key Laboratory of Biotechnology and Bioresources Utilization, State Ethnic Affairs Commission & Ministry of Education, Dalian Minzu University, Dalian 116600, China; E-mail: ruan@dlnu.edu.cn.

[†]These authors contributed equally to this article.

Abstract

Backgrounds: Yellowhorn (*Xanthoceras sorbifolium*) (NCBI Taxonomy ID: 99658) is a species of the Sapindaceae family in China. It is an oil tree that could sustain strictly cold and drought environments. As a tertiary legacy species, study of its genome will not only contribute to understand the evolution of genes and chromosomes, but also bring yellowhorn breeding into a genomic phase.

Findings: Here we generated 15 pseudomoleculars of the yellowhorn chromosomes, on which 97.04% of scaffolds anchored, using Illmina HiSeq, Pacifc Biosciences and Hi-C technologies. The final assembly genome of yellowhorn is 504.2 Mb with a contig N50 size of 1.04 Mb and a scaffold N50 size of 32.17 Mb. Genome annotation revealed that 68.67% of the yellowhorn genome is composed of repetitive elements. Gene modeling has predicted 24,672 protein-coding genes. Comparison of the identified orthologous genes estimated the divergence time of yellowhorn and its close sister species longan (*Dimocarpus Longan*) approximately at 38.79 mya. Gene clusters and chromosome synteny demonstrated that the yellowhorn genome conserved the genome structure of its ancestor in some chromosomes.

Conclusion: This genome assembly presented a high quality reference genome of yellowhorn. Integrated genome annotation provided a valuable data set for genetic and molecular research in this species. We did not detect the whole-genome duplication in this genome. The yellowhorn genome carried the fragment of its ancient chromosomes, reinforcing yellowhorn as a tertiary legacy species. All of these data sources will enable this genome to serve as an initial platform for breeding better yellowhorn.

Keywords

Xanthoceras sorbifolium, yellowhorn, PacBio sequencing, Genome assembly, Hi-C, Genome annotation, Conserved chromosome.

Data description

Background

Yellowhorn (*Xanthoceras sorbifolium*) was a tertiary legacy species [1], which belongs to the Sapindaceae family and the monotypic genus *Xanthoceras*. As an endemic woody oil species in Northern China, it was widely used for conserving soil and water due to the capacity to survive in arid, saline, alkaline land and in extreme temperature even below −40 °C [2, 3]. There are almost 7.5×10^5 ton yellowhorn seeds are being harvested in autumn every year [4] (**Fig.1**). The oil content of its seed kernel could be as high as 67%, of which 85%-93% is unsaturated fatty acid, including 37.1%-46.2% linoleic acid and 28.6%-37.1% oleic acid, which are essential fatty acids in diets [5]. Recently, yellowhorn as one of the major woody oil plant species has drawn government and people's attention again for the shortage of vegetable oil resources in China. Notably, an essential nutrient for brain growth and maintenance—nervonic acid, which is rarely contained in plants, reached nearly 3.04% in the seed oil of yellowhorn [6, 7]. More latest results indicate that xanthoceraside, a novel triterpenoidsaponin extracted from the husks of yellow horn, has an effect of antitumor and the potential to treat Alzheimerand [8-10]. In this study, we present the high-quality yellowhorn genome and conduct the annotation and genomic structures, evolution. The data provide a rich resource of genetic information for developing these resources and understanding the special space of *Xanthoceras* and Sapindaceae in plant evolution.

Sequenced individuals and sample collection

The yellowhorn cultivar Zhongshi 4, originated from Zhangwu of Liaoning Province, China, was developed by Research Institute of Forestry, Chinese Academy of Forestry and Zhangwu Deya Yellowhorn Professional Cooperatives for twelve years' breeding and selection. The fresh young leaves were collected from Zhongshi 4. The leaves were then frozen in liquid nitrogen and stored at -80°C until DNA extraction.

PacBio SMRT sequencing

Genomic DNA (gDNA) was extracted following ~40 kb SMRTbell™ Libraries Protocol (https://www.pacb.com/wp-content/uploads/Procedure-Checklist-Preparing-Greater-Than-30-kb-SMRTbell-Libraries-Using-Needle-Shearing-and-BluePippin-Size-Selection-on-Sequel-and-RSII-Systems.pdf). DNA was purified with Mobio PowerClean® Pro DNA Clean-Up Kit and quality was assessed by standard agarose gel electrophoresis and Thermo Fisher Scientific Qubit Fluorometry. Genomic DNA was sheared to a size range about 40 kb using g-TUBE (Covaris) and 0.45 × AMPure beads were used to enrich and purify large fragments of DNA. Damaged DNA and ends were enzymatically repaired as recommended by Pacific Biosciences. Following this procedure, hairpin adapters were ligated by blunt-end ligation reaction. The remaining damaged DNA fragments and those fragments without adapters at both ends were digested using exonuclease. Subsequently, the resulting SMRTbell templates were purified by Blue Pippin electrophoresis (Sage Sciences) and sequenced on a PacBio RS II instrument using P6-C4 sequencing chemistry. A primary filtering analysis was performed on the sequencer, and the secondary analysis was performed utilizing the SMRT analysis pipeline version 2.1.0 (Pacific Biosciences). In total, we generated a total of 66.44 Gb (roughly 122.83-fold of the yellowhorn genome) of single-molecule sequencing data (6,105,692 PacBio post-filtered reads), with an average read length of 10,882 bp (**Fig.S1; Table S1**).

Illumina short-read sequencing

DNA was extracted using DNA secure Plant Kit (TIANGEN, China) from leaf tissue of the same soil-grown seedlings of same plants (Zhongshi 4). Concentration and quality was assessed by 1% agarose gel electrophoresis and 2.0 Flurometer (Life Technologies, CA, USA). One shotgun library with an insert size of 350 bp was prepared using NEB Next® Ultra DNA Library Prep Kit (NEB, USA). Short reads were processed with Trimmomatic (version 0.33) [11,12] and Cutadapt (version 1.13) [13] to remove adapter sequences and leading and trailing bases with a quality score below 20 and reads with an average per-base-quality of 20 over a 4 bp sliding window. Reads < 70 nucleotides in length after

trimming were removed from further analysis and primary data analysis was carried out using the standard Illumina pipeline (HCS 2.0.12.0, RTA 1.17.21.3). A total of 119,550,151 reads were generated by Illumina HiSeq X Ten sequencing platform. This produced a total of 34.51 Gb (roughly 63.80-fold of the assembled genome) of raw sequencing data, with an average cleaned read length of 289 bp.

Estimation of the genome size by a K-mer analysis.

A K-mer analysis was performed to estimate the genome size, level of heterozygosity and repeat content of the sequenced genome as mentioned by Marçais [14]. All the generated PacBio and Illumina reads were filtered and corrected with Canu (version 1.5) [15], thereafter, using Jellyfish (version 2.0) [14] to assess the abundance of 17-mer to estimate the genome size of yellowhorn. The peak frequency of 17-mer was approximately 34 × depth for yellowhorn. The genome size was estimated to be approximately 536.58 Mb **(Fig.2a)** and the final cleaned data corresponded to the coverage of 63.79-fold. Repeat and error rates were estimated to be 60.21% and 0.02%, respectively, and heterozygosity rate was 0.36%, according to standard 17-mer curves of the yellowhorn genome.

Estimation of genome size through a flow cytometry analysis

The one-month-old leaves from the sequenced yellowhorn individual were put into a flow cytometry analysis to estimate genome size as mentioned by Galbraith [16]. Over 3,000 nuclei were analyzed per sample with a FACSAria flow cytometer (Becton, Dickinson and Company). A total of 16 samples were analyzed using poplar (*Populus trichocarpa*) as the standard species. The software BDFACSDiva (version 8.0.1) was used for data analysis with the coefficient variation controlled in 5%. The mean peak value of the fluorescence intensity of 16 yellowhorn samples is at round 11,558, while that of poplar is at around 10,363. Referencing the poplar genome size at 485 Mb [17], the yellowhorn genome size was estimated to be approximately 540.93 ± 11.15 Mb (**Fig.2b**) [18].

Genome assembly

After stringent filtering and correction steps using K-mer frequency-based methods [19], we assembled the yellowhorn genome using Pacbio reads. Primary assemblies generated a total length of 598.65 Mb of contigs with a N50 length of 1.11 Mb, derived from the 66.44 Gb PacBio long reads corrected with Falcon v0.7 (https://github.com/PacificBiosciences/FALCON/wiki/Manual). The software Quiver (based on pbsmrtpipe.pipelines.sa3_ds_resequencing in smrtlink_5.0.1; http://pbsmrtpipe.readthedocs.io/en/master/getting_started.html) is used to polish the consensus sequence clusters. The assemblies were corrected by the Pilon (version 1.22) (https://github.com/broadinstitute/pilon/wiki) using the Illumina short reads. When the heterozygous sequences were removed, the final assemblies (504.20 Mb, with a contig N50 of 1.39 Mb) were generated (**Table 1**).

Pseudomolecules construction and three-dimensional chromatin conformation analysis

Hi-C technology is an efficient strategy for pseudomolecule construction and enables the generation of genome-wide three-dimensional architecture of chromosomes. We constructed Hi-C fragment libraries of 350 bp and sequenced them using the Illumina Hi-seq platform (Illumina, San Diego, CA, USA) for chromosome pseudomolecule construction. Mapping of the Hi-C reads and assignment to restriction fragments were performed as described in Burton [20]. A total of 53.39 Gb of trimmed reads, accounting for around 98.70-fold coverage of the yellowhorn genome, were mapped to the assemblies with aligner BWA (version 0.7.10) [21]. Only uniquely-aligned reads with high alignment quality (>20) were selected for the construction of the pseudomolecular. Duplicate removal and quality assessment were performed with HiC-Pro (version 2.8.1) [22]. The 50.56% of Hi-C data were grouped into the valid interaction pairs. A total of 2,836 contigs (N50 length at 1.04 Mb) were generated after error correction. LACHESIS (parameters: cluster_min_re_sites=48; cluster_max_link_density=2; cluster_noninformative_ratio =2; order_min_n_res_in_trun=14; order_min_n_res_in_shreds=15) [20] was used to assign the order and orientation of each group, with a scaffold N50 of

32.17 Mb. Using the 98.70-fold coverage of Hi-C reads, 489.28 Mb (97.04%) of the assemblies were anchored onto the 15 pseudomoleculars, of which 477.59 Mb (94.76%) was ordered by frequency distribution of valid interaction pairs (**Table 2, Fig.S2**).

Transcriptome sequencing

RNA was extracted from four tissues, flowers, leaves, stems and roots using the easy spin RNA extraction kit (Sangon Biotech, Shanghai, China; No. SK8631). The concentration of each RNA sample was checked using NanoDrop (Thermo Fisher Scientific Inc., USA) and the QUBIT ® Fluorometer (Life Technologies). The RNA integrity was checked using a Bioanalyzer 2100 (Agilent Technologies). The Iso-Seq libraries were prepared according to the Isoform Sequencing protocol (Iso-Seq) using the Clontech SMARTer PCR cDNA Synthesis Kit and the BluePippin Size Selection System protocol as described by Pacific Biosciences (PN 100-092-800-03). Mixed Sample was sequenced using on the Pacific Biosciences RS II platform with one SMRT cell v3 each based on P6-C4 chemistry.

Sequence data were processed using the SMRTlink 4.0 software. Circular consensus sequences were derived from the subread BAM files with the parameters: min_length 200, max_drop_fraction 0.8, no_polish TRUE, min_zscore -999, min_passes 1, min_predicted_accuracy 0.8, max_length 18000. Separation of the full length and non-full length reads were conducted using pbclassify.py (ignorepolyA false, minSeqLength 200). Non-full length and full-length fasta files produced were then fed into the cluster step to cluster the isoforms, followed by final Arrow polishing with the parameters of hq_quiver_min_accuracy 0.99, bin_by_primer false, bin_size_kb 1, qv_trim_5p 100, qv_trim_3p 30. The LoRDEC software (version 0.3) was used to correct sequencing errors in the consensus transcripts using Illumina reads as the reference (parameters: -k 19 -s 3) [23]. The corrected consensus transcripts were clustering by CD-HIT (version 4.6.8) [24] to reduce sequences redundancy and improve the performance of other sequence analyses. A total of 142,396 transcripts were generated in the final RNA assemblies, which were used as evidence to assist the gene prediction.

Evaluation of assemble quality

The completeness of the final assemblies was evaluated using CEGMA (version 2.5) [25] (http://korflab.ucdavis.edu/dataseda/) and BUSCO (version 3.0.2) [26-27] (https://gvolante.riken.jp/analysis.html), respectively. The CEGMA outputs display a 94.76% of core eukaryotic genes (235 out of 248 core eukaryotic genes) in our assemblies. The BUSCO test, referencing the embryophyta protein set (run_BUSCO.py -i plant_species.fa -o plant_species-l embryophyta_odb9/-m proteins), exhibit that 94.7% of plant gene sets were identified as complete (1364 out of 1440 BUSCOs), including 83.2% single-copy and 11.5% duplicated genes (**Table S2**). All of these results suggested a complete and robust yellowhorn genome assembly.

Annotation of the repetitive sequences.

A *de novo* repeat database was constructed using RepeatScout (version 1.0.5) [28]. RepeatMasker (version 4.0.7) [29] was utilized with the following parameters "-nolow -no_is -norna -engine wublast" to identify repeat sequence against the *de novo* repeat library, as well as Repbase (version 19.06) [30]. The genome was also scanned using LTR-FINDER (version 1.0.5) [31], MITE-Hunter (version 1.0) [32] and PILER (version 1.0) [33]. The predicted repeats were classified using PASTEClassifier (version 1.0) [34].

The predicted repeats occupied 346.39 Mb (68.67%) of the yellowhorn genome in length, which was slightly larger than the 52.78% of longan (*D. longan*) [35] and much larger than the 20% of Clementine (*C. sinensis*) [36]. Of these repeats, two types of the LTR-retrotransposons are the most abundant, 98.68 Mb of Copia (19.56%) and 88.24 Mb of Gypsy (17.49%) **(Table S3)**. Accumulation of LTR-retrotransposons is an important contributor to genome expansion and diversity [37]. The insertion time of the LTR-retrotransposons in the genomes is estimated by calculation of sequence variance between the LTR arms of each LTR-retrotransposon, utilizing the substitution rate of 1.3×10^{-8} substitutions per site per year [38]. A comparison of the insertion ages for the LTR-retrotransposons illustrated a similar insertion profiles among the genomes of clementine, longan, grape (*V. vinifera*) and yellowhorn **(Fig. 3a)**. We observed

that the yellowhorn genome carried more young LTR-retrotransposons, which were accounted for the highest proportion with insertion ages less than 0.2 mya. This might be resulting from the rapid changes of the growing environment, such as the effect from pathogens and the interference with human activities in the recent years. Besides, the yellowhorn and grape genomes showed much more LTR-retrotransposons than the other two (clementine and longan), reflecting their higher genome sequence quality, yellowhorn sequenced by a combination of PacBio Single-Molecule Real-Time and Illumina Hiseq short-read sequencing platforms and grape sequenced by Sanger sequencing technology [39]. The genomes sequenced by pure next-generation sequencing technology might lose more LTR-retrotransposons because the sequencing similarity between LTR arms and among different LTR-retrotranpsons probably caused the assembly errors of these regions, which led to an under-estimated quantity of the LTR-retrotransposons in clementine and longan.

Prediction of RNA genes

Gene annotation in the yellowhorn genome was conducted by combining *de novo* prediction, homology information, and RNA-seq data. For the *de novo* prediction, Genscan (version 3.1) [40], Augustus (version 3.1) [41], GlimmerHMM (version 3.0.4) [42], GeneID (version 1.4) [43], SNAP (version 2006-07-28) [44] were used on the repeat masked genome with parameters trained for Arabidopsis (*A. thaliana*). For the homology-based prediction, the Uniprot protein sequences from the 3 sequenced plants, Arabidopsis, longan and grape, were used as the reference databases aligned the homolog genes in the yellowhorn genome using GeMoMa (version 1.3.1) [45]. The RNA-seq data were aligned to the reference genome by TransDecoder (version 2.0) (http://transdecoder.github.io) [46] and GeneMarkS-T (version 5.1) [47] under default parameters. Unigenes assembled from the RNA-seq data were aligned to the reference genome using PASA (version 2.0.2) [48] to annotate protein-coding genes. To finalize the gene set, all predictions were combined with EVidenceModeler (v1.1.1) [46] to produce a consensus gene set. Finally, the *ab initio* predicted transcripts were assigned to the PASA predicted transcripts from unigenes and GeMoMa predicted homologous transcripts to add the

lost genes when conducting the EVM integration.

Then the RNA-seq reads were aligned to the reference genome by TopHat (v2.0.10, implemented with bowtie2) [49] to identify candidate exon regions and splicing donor and acceptor sites to evaluate the results of gene prediction. Infernal (version 1.1) [50] were used to identify the rRNA and microRNA based on Rfam (version 12.1) [51] and miRbase (version 21) [52]. TRNAscan-SE (version 1.3.1) [53] were also used to identify the tRNA.

GenBlastA was used to pseudogene prediction by scanning the yellowhorn genome for sequences homologous to the known protein-coding genes it contained, and premature stop codons or frame shift mutations in those sequences were searched by GeneWise (version 2.4.1) [54-55].

The genes were annotated by aligning to the NR, KOG, GO, KEGG, TrEMBL databases using BLAST (version 2.2.31) with an e-value cutoff of 10^{-5} and also aligned to the Pfam database using Hmmer (version 3.0) (parameters, -E 0.00001 --domE 0.00001 --cpu 2 --noali –acc)] [55-61]. GO terms were allocated to the genes using Blast2GO pipeline [58].

In total, we annotated 24,672 protein-coding genes in the yellowhorn genome (**Table S4**) and 1,913 Pseudogenes, with average gene length of 4,199 bp, average intron length of 2,560 bp and average coding sequence length of 1,580 bp. Of these genes, 98.97% (24,429) carried at least one functional domain with the alignments to the protein database (**Table S5**). Their functions were classified by GO terms (**Fig. S3**) and KOG database (**Fig. S4**). In addition, 642 tRNA, 108 microRNA and 316 rRNA genes were predicted in the yellowhorn genome.

Identification of gene clusters and duplication

Gene clustering was conducted using OrthoMCL (version 5) [62] among the protein sequences of ten typical dicot genomes representative of Rutaceae (*Citrus clementina*) [36], Sapindaceae (*D. Longan*) [35], Cruciferous (*Brassica napus* and *Arabidopsis thaliana*) [63, 64], Sterculiaceae (*Theobroma cacao*) [65], Malvaceae (*Gossypium raimondii*) [66], Fagaceae (*Quercus robur*) [67], Vitaceae (*Vitis vinifera*) [68], Cucurbitaceae (*Cucumis sativus*) [69] and

Rosaceae (*Malus* × *domestica*) [70] families, as well as yellowhorn. The yellowhorn genes were clustered into a total of 14,667 gene families, including 172 yellowhorn-specific gene families. Comparison of copy numbers in gene clusters of these eleven dicot genomes indicated that the yellowhorn genome had the similar proportion of the single and multiple copy genes with other analyzed genomes (**Fig. 3b**), except the tetraploid *B. napus* genome [71]. The species-special genes of yellowhorn were similar to *T. cacao*, both of which were much less than other species. It is implicated that the yellowhorn genes might keep more structural characters of their ancestors, which suggested that yellowhorn is a relic species of the Tertiary.

A total of 3,367 single-copy gene families were identified and chosen to construct the phylogenetic tree using PHYML (version 3.0) (**Fig. 3c**) [72]. The Software Muscle (version 3.8.31) (https://www.ebi.ac.uk/Tools/msa/muscle/) [73] was used to align the orthologs. Alignment outputs were treated with Gblocks (version 14.1) with the parameters of -t = p -b5 = h -b4 = 5 -b3 = 15 -d = y -n= y [74]. The divergence time was estimated by MCMCtree (version 4.7a) [75]. As two species of Sapindaceae Family, yellowhorn and longan are indicative of the closest relationship, with the divergence time estimated at approximately 38.79 mya. Using the orthologous gene pairs of yellowhorn and longan identified by gene collinearity and paralogous pairs identified by gene cluster, the 4DTv (four-fold degenerate synonymous sites of the third codons) were calculated for all of these duplicated pairs. A species divergence peak (4DTv~0.1) was exhibited in yellowhorn vs. longan ortholog 4DTv distribution but no obvious peak could be seen in the yellowhorn paralog curse and longan paralog curse (**Fig. 3d**). The self-alignment of the chromosomes based on the identified gene synteny, no large-scale gene duplication can be found in the yellowhorn genome (**Fig. S2**). Correspondingly, the yellowhorn genome did not undergo the whole-genome and large-fragment duplication.

Chromosome synteny between yellowhorn and reference genomes.

To investigate evolution of the yellowhorn chromosomes, yellowhorn vs. arabidopsis, yellowhorn vs. clementine and

yellowhorn vs. grape chromosome syntenies were constructed according to the gene collinearity using aligner MCscan (version 0.8) [76], respectively. A total of 367, 409 and 386 syntenic blocks were identified on the basis of the orthologous gene orders, corresponding to 28,372, 18,650 and 23,400 genes in each genome, respectively. Correspondingly, average gene number per each block was 77.3, 45.6 and 60.6 genes, respectively. This suggested the highest collinearity between the genomes of yellowhorn and clementine, which was consistent to their Sapindale clade of the phylogenetic relationship. Alignments of syntenic chromosomes were visualized between yellowhorn and the other three genomes. Frequency of the large-scale fragment rearrangements, including inversions and translocations, between yellowhorn and clementine displayed considerably lower than the other two (**Fig. 4**). Especially, structural variation between yellowhorn and grape was so frequent that it is too difficult to speculate the syntenic relationship among the chromosomes (**Fig. 4b**). The concluded chromosome alignments between yellowhorn linkage groups and clementine pseudomolecular revealed that most of cross-chromosome rearrangements were different from that between yellowhorn and Arabidopsis (**Fig. 4d, 4e**). It was found that yellowhorn Linkage group 2 and 11 are syntenic to a single clementine pseudomolecular, Scaffold 5 and 3, respectively, and the Linkage groups 3, 4, 5, 7, 8, 10, 12, 14 and 15 were aligned to two reference chromosomes of clementine. Comparatively, frequency of chromosome rearrangement was a little higher between yellowhorn linkage groups and Arabidopsis chromosomes. The Arabidopsis Chromosome 1 is predominantly systemic to yellowhorn Linkage group 4, which demonstrated that the yellowhorn genomes conserved some genome structure of its originals (**Fig. 4d**). Intriguingly, the similar chromosomal fusion events were found among some chromosomes. Aligned fragments of Arabidopsis Chromosomes 1, 3 and 5 fused to form yellowhorn linkage groups 1 and 14, which was the same as clementine Scaffolds 1, 2 and 3. Yellowhorn Linkage group 6 was aligned to clementine scaffolds 1, 3, 4 and 6, but had extensive collineartiy with Arabidopsis Chromosome 3 (**Fig. 4d, 4e**). These findings suggested that the same ancestors were shared among these chromosomes, despite of the extensive rearrangements. In general, the yellowhorn genome carried the fragment of its ancient chromosomes, as mentioned above analysis of genes structural characters, reinforcing yellowhorn as a tertiary legacy species.

Abbreviations:

bp: base pair; BUSCO: Benchmarking Universal Single-Copy Ortholog; CDS: coding sequence; GO: Gene Ontology; kb: kilobases; KEGG: Kyoto Encyclopedia of Genes and Genomes; LTR: long terminal repeat; Mb: megabases; Mya: million years ago; NCBI: National Center for Biotechnology Information; PE: paired-end; RNA-Seq: RNAsequencing; SMRT: Single-Molecule Real-Time; SRA: Sequence Read Archive.

Additional File

Additional file 1: Tables S1 to S5

Table S1: Statistics of PacBio data.

Table S2: Genome quality assessed by the BUSCO test.

Table S3: Content of repetitive sequences.

Table S4: Prediction of protein-coding genes.

Table S5: Function annotation of the protein-coding genes.

Additional file 2: Figures S1 to S4

Figure S1: Length distribution of three types of the produced PacBio reads.

Figure S2: Interaction frequency distribution of Hi-C links among chromosomes.

Figure S3: Function classification of the protein-coding genes against the GO term database.

Figure S4: KOG function classification of the protein-coding genes.

Funding

This work was financially supported by the Central Public-Interest Scientific Institution Basal Research Fund (CAFYBB2017QB001), the National “12th Five-Year” Plan for Science & Technology Support of China

(2015BAD07B0106), the National Natural Science Foundation of China (31800571, 31870594, 31760213), the National Key Research and Development Plan of China (2016YFC050080506), the Key research and development plan of Liaoning Province (2017204001) and the Beijing ABT Biotechnology Co., Ltd.

Availability of supporting data

The raw sequence data have been deposited in NCBI under project accession number PRJNA483857, the Short Read Archive (SRA) accession number was SRP159119 (SRR7768197, SRR7768198, SRR7768199, SRR7768201), The accession number of *Xanthoceras sorbifolium* Genome sequencing and assembly was QUWJ 00000000. All supplementary figures and tables are provided in Additional Files.

Conflict of Interest

The authors declare that they have no competing financial interests.

Author Contributions

QXB, HYY, YL, CJR and LBW conceived and designed the study; TPC, XJL,YCL, SQF, XYH, GHF, YFC, JD and DSC prepared materials and conducted the experiments; QXB, YZ, WD, YL and LG wrote the manuscript.

Legends

Fig.1 Images of the yellowhorn plants. (**a**)The yellowhorn tree in artificial forest. (**b**)The mellow fruit, will dehisce in three parts by carpel. (**c**) A harvest scene of yellowhorn in northern China. (**d**) The seed in the mellow fruit, which number is 18-24 in one fruit.

Fig.2 Estimation of the genome size. (**a**) Distribution of 17-mer frequency. Values for K-mers are plotted against the frequency (y axis) of their occurrence (x axis). The leftmost truncated peak at low occurrence (1-2) was mainly due to random base errors in the raw sequencing reads. (**b**) Test results of the yellowhorn and poplar samples using flow cytometry.

Fig.3 Genome evolution. (**a**) Distribution of insertion ages of LTR-retrotransposons. The *x*-axis represents the estimated insertion age (mya) of the LTR-retrotransposons. The *y*-axis represents the number of intact LTR-retrotransposons. (**b**) Comparison of copy numbers in gene clusters of analyzed dicot genomes. According to the identified gene clusters, the genes are grouped into single-copy, multiple-copy and species-specific (specific). (**c**) Constructed phylogenetic tree and divergence time estimation. The numbers represent estimated divergence times (mya) which are measured by a bar of 20 million years. The cucumber (*C. sativus*) is used as outgroup. (**d**) Genome duplication in dicot genomes as revealed through 4DTv analyses. The 4DTv of the orthologous pairs (Y vs. L) between yellowhorn (Y) and longan (L) and paralogous gene pairs within the yellowhorn (Y vs. Y) and longan genome (L vs. L) are plotted against their calculated 4DTv values.

Fig.4 Chromosome synteny. (**a**) Chromosome alignment of yellowhorn and Arabidopsis. (**b**) Chromosome alignment of yellowhorn and grape. (**c**) Chromosome alignment of yellowhorn and clementine. Colored ribbons connect the aligned genes. yellowhorn linkage groups are labeled as LG 1 to 15, Arabidopsis chromosomes labeled as Chr 1 to 5, grape chromosomes labeled as C1 to 19 and CUn (location of the chromosomes are unknown) and clementine labeled as Sc 1 to 9. Scale, 10Mb. (**d**) Chromosome rearrangement between Arabidopsis and yellowhorn. (**e**) Chromosome rearrangement between clementine and yellowhorn. Arabidopsis

and clementine chromosomes are represented as the bars filled with different colors. Synteny and rearrangement of the yellowhorn chromosomes are indicated by different blocks, corresponding to referenced Arabidopsis and clementine chromosomes.

References

[1] Wang Q, Yang L, Ranjitkar S et al. Distribution and in situ conservation of a relic Chinese oil woody species yellowhorn *Xanthoceras sorbifolium* Bunge. Can J For Res 2017; 47: 1450-6.

[2] Board E. Flora of China vol.47. 1985; 47(1): 72.

[3] Yu H, Fan S, Bi Q et al. Seed morphology, oil content and fatty acid composition variability assessment in yellow horn (*Xanthoceras sorbifolium* Bunge) germplasm for optimum biodiesel production. Ind Crop Prod 2017; 97: 425-30.

[4] Yao ZY, Qi JH, Yin LM, et al. Biodiesel production from *Xanthoceras sorbifolia* in China: Opportunities and challenges. Renew Sust Energy Rev 2013; 24: 57-65.

[5] Venegas-Calerón M, Ruíz-Méndez MV, Martínez-Force E et al. Characterization of *Xanthoceras sorbifolium* Bunge seeds: Lipids, proteins and saponins content. Ind Crop Prod 2017; 109(1): 192-8.

[6] Krishnan H. Modification of seed composition to promote health and nutrition. Crop Sci Soc Amer 2009: 263-71.

[7] Taylor DC, Guo Y, Katavic V et al. New Seed Oils for Improved Human and Animal Health: Genetic Manipulation of the Brassicaceae for Oils Enriched in Nervonic Acid. Modification of Seed Composition to Promote Health and Nutrition 2009: 51.

[8] Qi Y, Ji XF, Chi TY et al. Xanthoceraside attenuates amyloid β peptide 1-42 -induced memory impairments by reducing neuroinflammatory responses in mice. Eur J Pharmacol 2017; 820: 18-30.

[9] Ji XF, Chi TY, Liu P et al. The total triterpenoid saponins of *Xanthoceras sorbifolia* improve learning and memory impairments through against oxidative stress and synaptic damage. Phytomedicine 2017; 25: 15-24.

[10] Zhang Y, Xiao LU, Xiao B et al. Research progress and application prospect of *Xanthoceras sorbifolia* for treating Alzheimer's disease. Drug Eval Res 2018; 25(05): 912-7.

[11] Alberto CM, Sanso AM, Xifreda CC et al. Chromosomal studies in species of Salvia (Lamiaceae) from Argentina. Bot J Linn Soc 2015; 141(4): 483-90.

[12] Bolger AM, Lohse M, Usadel B et al. Trimmomatic: a flexible trimmer for Illumina sequence data. Bioinformatics 2014; 30(15): 2114-20.

[13] Martin M. Cutadapt removes adapter sequences from high-throughput sequencing reads. Embnet J 2011; 17(1): 10-2.

[14] Marçais G, Kingsford C. A fast, lock-free approach for efficient parallel counting of occurrences of k-mers. Bioinformatics 2011; 27(6): 764-70.

[15] Koren S, Walenz BP, Berlin K et al. Canu: scalable and accurate long-read assembly via adaptive k-mer weighting and repeat separation. Genome Res 2017; 27(5): 722-36.

[16] Galbraith DW, Harkins KR, Maddox JM et al. Rapid flow cytometric analysis of the cell cycle in intact plant tissues. Science 1983; 220(4601): 1049-51.

[17] Tuskan GA, Difazio S, Jansson S, et al. The genome of black cottonwood, *Populus trichocarpa* (Torr. & Gray). Science 2016; 313(5793): 1596-604.

[18] Dolezel J, Greilhuber, J, Suda, J, et al. Estimation of nuclear DNA content in plants using flow cytometry. Nat Protoc 2007; 2(9): 2233-44.

[19] Li R, Fan W, Tian G et al. The sequence and de novo assembly of the giant panda genome. Nature 2010; 463(7279): 311-29.

[20] Burton J N, Adey A, Patwardhan RP et al. Chromosome-scale scaffolding of de novo genome assemblies based on chromatin interactions. Nat Biotechnol 2013; 31(12): 1119-25.

[21] Li H, Durbin R et al. Fast and accurate short read alignment with BurrowsZ Wheeler transform. Bioinformatics, 2009; 25(14): 1754-60

[22] Servant N, Varoquaux N, Lajoie BR et al. HiC-Pro: an optimized and flexible pipeline for Hi-C data processing. Genome Biol 2015; 16(1): 259-70.

[23] Salmela L, Rivals E. LoRDEC: accurate and efficient long read error correction. Bioinformatics 2014; 30(24):

3506-14.

[24] Fu L, Niu B, Zhu Z et al. CD-HIT: accelerated for clustering the next-generation sequencing data. Bioinformatics 2012; 28(23): 3150-2.

[25] Parra G, Bradnam K, Korf I et al. CEGMA: a pipeline to accurately annotate core genes in eukaryotic genomes. Bioinformatics 2007; 23(9): 1061-7.

[26] Simao FA, Waterhouse RM, Ioannidis P et al. BUSCO: assessing genome assembly and annotation completeness with single-copy orthologs. Bioinformatics 2015; 31(19): 3210-2.

[27] Nishimura O, Hara Y, Kuraku S. Volante for standardizing completeness assessment of genome and transcriptome assemblies. Bioinformatics 2017;33(22): 3635-3637.

[28] Price AL, Jones NC, Pevzner PA et al. De novo identification of repeat families in large genomes. Bioinformatics 2005; 21 (suppl_1): i351.

[29] Tarailo-Graovac M, Chen N. Using RepeatMasker to identify repetitive elements in genomic sequences. Curr Protoc Bioinformatics 2004; Chapter 4(Unit 4): 4-10.

[30] Jurka J, Kapitonov VV, Pavlicek A et al. Repbase Update, a database of eukaryotic repetitive elements. Cytogenet Genome Res 2005; 110(1-4): 462-7.

[31] Xu Z, Wang H. LTR_FINDER: an efficient tool for the prediction of full-length LTR retrotransposons. Nucleic Acids Res 2007; 35(Web Server issue): 265-8.

[32] Han Y, Wessler SR. MITE-Hunter: a program for discovering miniature inverted-repeat transposable elements from genomic sequences. Nucleic Acids Res 2010; 38(22): e199.

[33] Edgar RC, Myers EW et al. PILER: identification and classification of genomic repeats. Bioinformatics 2005; 21 (suppl_1): i152.

[34] Wicker T, Sabot F, Hua-Van A et al. A unified classification system for eukaryotic transposbale elments. Nat rev Genet 2007; 8(12): 973-82.

[35] Lin Y, Min J, Lai R et al. Genome-wide sequencing of longan (*Dimocarpus longan* Lour.) provides insights into molecular basis of its polyphenol-rich characteristics. Gigascience 2017; 6(5): 1-14.

[36] Wu GA, Prochnik S, Jenkins J et al. Sequencing of diverse mandarin, pummelo and orange genomes reveals complex history of admixture during citrus domestication. Nat Biotechnol 2014; 32(7): 656-62.

[37] Kidwell MG, Lisch D. Transposable elements as sources of variation in animals and plants. Proc Natl Acad Sci U S A. 1997; 94(15): 7704-11.

[38] Zuccolo A, Sebastian S, Yu Y et al. Assessing the Extent of Substitution Rate Variation of Retrotransposon Long Terminal Repeat Sequences in *Oryza sativa* and *Oryza glaberrima*. Rice, 2010; 3 (4): 242-50.

[39] Jaillon O, Aury JM, Noel B et al. The grapevine genome sequence suggests ancestral hexaploidization in major angiosperm phyla. Nature 2007; 449(7161): 463-7.

[40] Burge C, Karlin S et al. Prediction of complete gene structures in human genomic DNA. J mol boil 1997; 268(1): 78-94.

[41] Stanke M, Waack S et al. Gene prediction with a hidden Markov model and a new intron submodel. Bioinformatics 2003; 19(suppl_2): 215-25.

[42] Majoros WH, Pertea M, Salzberg S L et al. Tigr Scan and Glimmer HMM: two open source ab initio eukaryotic gene-finders. Bioinformatics 2004; 20(16): 2878-9.

[43] Blanco E, Parra G, Guigó R et al. Using geneid to identify genes. Cur Protoc Bioinformatics 2007; 18(1): 3-4.

[44] Korf I et al. Gene finding in novel genomes. BMC Bioinformatics 2004; 5(1): 59-68.

[45] Jens K, Michael W, Erickson J L et al. Using intron position conservation for homology-based gene prediction. Nucleic Acids Res 2016; 44(9): e89.

[46] Haas BJ, Papanicolaou A, Yassour M et al. De novo transcript sequence reconstruction from RNA-seq using the Trinity platform for reference generation and analysis. Nat Protoc 2013; 8(8): 1494-512.

[47] Tang S, Lomsadze A, Borodovsky M et al. Identification of protein coding regions in RNA transcripts. Nucleic

Acids Res 2014; 43(12): 58-68.

[48] Campbell MA, Haas BJ, Hamilton JP et al. Comprehensive analysis of alternative splicing in rice and comparative analyses with Arabidopsis. BMC Genomics 2006; 7(1): 327-43.

[49] Trapnell C, Pachter L, Salzberg S L et al. TopHat: discovering splice junctions with RNA-Seq. Bioinformatics 2009; 25(9): 1105-11.

[50] Nawrocki EP, Eddy SR et al. Infernal 1.1: 100-fold faster RNA homology searches. Bioinformatics 2013; 29(22): 2933-5.

[51] Griffiths-Jones S, Moxon S, Marshall M et al. Rfam: annotating non-coding RNAs in complete genomes. Nucleic Acids Res 2005; 33(Database issue): 121-4.

[52] Griffithsjones S, Grocock RJ, Van DS et al. miRBase: microRNA sequences, targets and gene nomenclature. Nucleic Acids Res 2006; 34(Database issue): 140-4.

[53] Lowe TM, Eddy SR et al. tRNAscan-SE: a program for improved detection of transfer RNA genes in genomic sequence. Nucleic Acids Res 1997; 25(5): 955-64.

[54] She R, Chu JK, Pei J et al. GenBlastA: enabling BLAST to identify homologous gene sequences. Genome Res 2009; 19(1):143-9.

[55] Birney E, Clamp M, Durbin R et al. GeneWise and Genomewise. Genome Res 2004; 14(5): 988-96.

[56] Marchlerbauer A, Lu S, Anderson JB et al. CDD: a Conserved Domain Database for the functional annotation of proteins. Nucleic Acids Res 2011; 39(Database issue): 225-9.

[57] Tatusov RL, Natale DA, Garkavtsev IV et al. The COG database: new developments in phylogenetic classification of proteins from complete genomes. Nucleic Acids Res 2001; 29(1): 22-8.

[58] Dimmer EC, Huntley RP, Alamfaruque Y et al. The UniProt-GO Annotation database in 2011. Nucleic Acids Res 2012; 40(Database issue): 565-70.

[59] Marisa L, De Reynies A, Duval A et al. KEGG: Kyoto encyclopedia of genes and genomes. 2013.

[60] Boeckmann B, Bairoch A, Apweiler R et al. The Swiss-Prot knowledgebase and its supplement TREMBL in 2003. Nucleic Acids Res 2003; 31(1): 365-70.

[61] Altschul S, Gish W, Miller W et al. Basic local alignment search tool. J Mol Biol 1990; 215:403-10

[62] Li L, Jr SC, Roos DS et al. OrthoMCL: identification of ortholog groups for eukaryotic genomes. Genome Res 2003; 13(9): 2178-89.

[63] Chalhoub B, Denoeud F, Liu S et al. Early allopolyploid evolution in the post-Neolithic *Brassica napus* oilseed genome. Science 2016; 345(6199): 950-3.

[64] Theologis A, Ecker JR, Palm CJ et al. Sequence and analysis of chromosome 1 of the plant *Arabidopsis thaliana*. Nature 2000; 408(6814): 816-20.

[65] Argout X, Salse J, Aury JM et al. The genome of *Theobroma cacao*. Nat Genet 2013; 43(2):101-8.

[66] Wang K, Wang Z, Li F et al. The draft genome of a diploid cotton *Gossypium raimondii*. Nat Genet 2012; 44(10):1098-103.

[67] Plomion C, Aury JM, Amselem J et al. Oak genome reveals facets of long lifespan. Nat Plants 2017; 4: 440-52.

[68] Jaillon O, Aury JM, Noel B et al. The grapevine genome sequence suggests ancestral hexaploidization in major angiosperm phyla. Nature 2007; 449(7161): 463-7.

[69] Huang S, Li R, Zhang Z et al. The genome of the cucumber, *Cucumis sativus* L. Nat Genet 2009; 41(12): 1275-81.

[70] Velasco R, Zharkikh A, Affourtit J et al. The genome of the domesticated apple (*Malus* × *domestica* Borkh.). Nat Genet 2010; 42(10): 833-9.

[71] Cheung F, Trick M, Drou N et al. Comparative analysis between homoeologous genome segments of *Brassica napus* and its progenitor species reveals extensive sequence-level divergence. Plant Cell 2009; 21(7): 1912-28.

[72] Guindon S, Dufayard JF, Lefort V et al. New algorithms and methods to estimate maximum-likelihood phylogenies: assessing the performance of PhyML 3.0. Syst Biol 2010; 59(3): 307-21.

[73] Edgar RC. MUSCLE: Multiple sequence alignment with high accuracy and high throughput. Nucleic Acids Res

2004; 32: 1792-7.

[74] Talavera G, Castresana J et al. Improvement of phylogenies after removing divergent and ambiguously aligned blocks from protein sequence alignments. Syst Biol 2007; 56(4): 564-77.

[75] Battistuzzi FU, Billingross P, Paliwal A et al. Fast and slow implementations of relaxed-clock methods show similar patterns of accuracy in estimating divergence times. Mol Biol Evol 2011; 28(9): 2439-42.

[76] Tang HB, Bowers JE, Wang XY, et al. Perspective- Synteny and collinearity in plant genomes. Science 2008; 320(5875): 486-8.

Table1 Overview of assembly and annotation for the yellowhorn genome.

Total length	504,196,643 bp
Length of unclosed gaps	73,800 bp
N50 length (initial contigs)	1,044,891 bp
N50 length (scaffolds)	32,173,403 bp
N90 length (scaffolds)	25,069,408 bp
Quantity of scaffolds (＞N90 length)	21
Largest scaffold	40,097,451 bp
GC content	36.95%
Quantity of predicted protein-coding genes	24,672
Quantity of predicted noncoding RNA genes	1,066
Content of repetitive sequences	68.67%
Length of genome anchored on linkage groups	489,286,946 bp (97.04%)

 Click here to access/download;Table;Table 2.xlsx

Table 2. Quantity of the contigs anchored with Hi-C.

Group	Quatity of anchored contigs	Sequence Length (bp)
Lachesis Group 1	68	40,738,791
Lachesis Group 2	92	40,039,835
Lachesis Group 3	38	37,159,809
Lachesis Group 4	112	35,552,403
Lachesis Group 5	84	35,291,867
Lachesis Group 6	62	35,706,508
Lachesis Group 7	66	33,002,525
Lachesis Group 8	46	32,947,898
Lachesis Group 9	66	30,804,552
Lachesis Group 10	62	30,699,318
Lachesis Group 11	68	29,306,026
Lachesis Group 12	56	29,390,540
Lachesis Group 13	47	29,816,145
Lachesis Group 14	71	25,601,946
Lachesis Group 15	72	23,228,783
Total (Ratio %)	1,010 (35.61)	489,286,946 (97.04)

Figure 1

Click here to access/download;Figure;Fig.1.tif

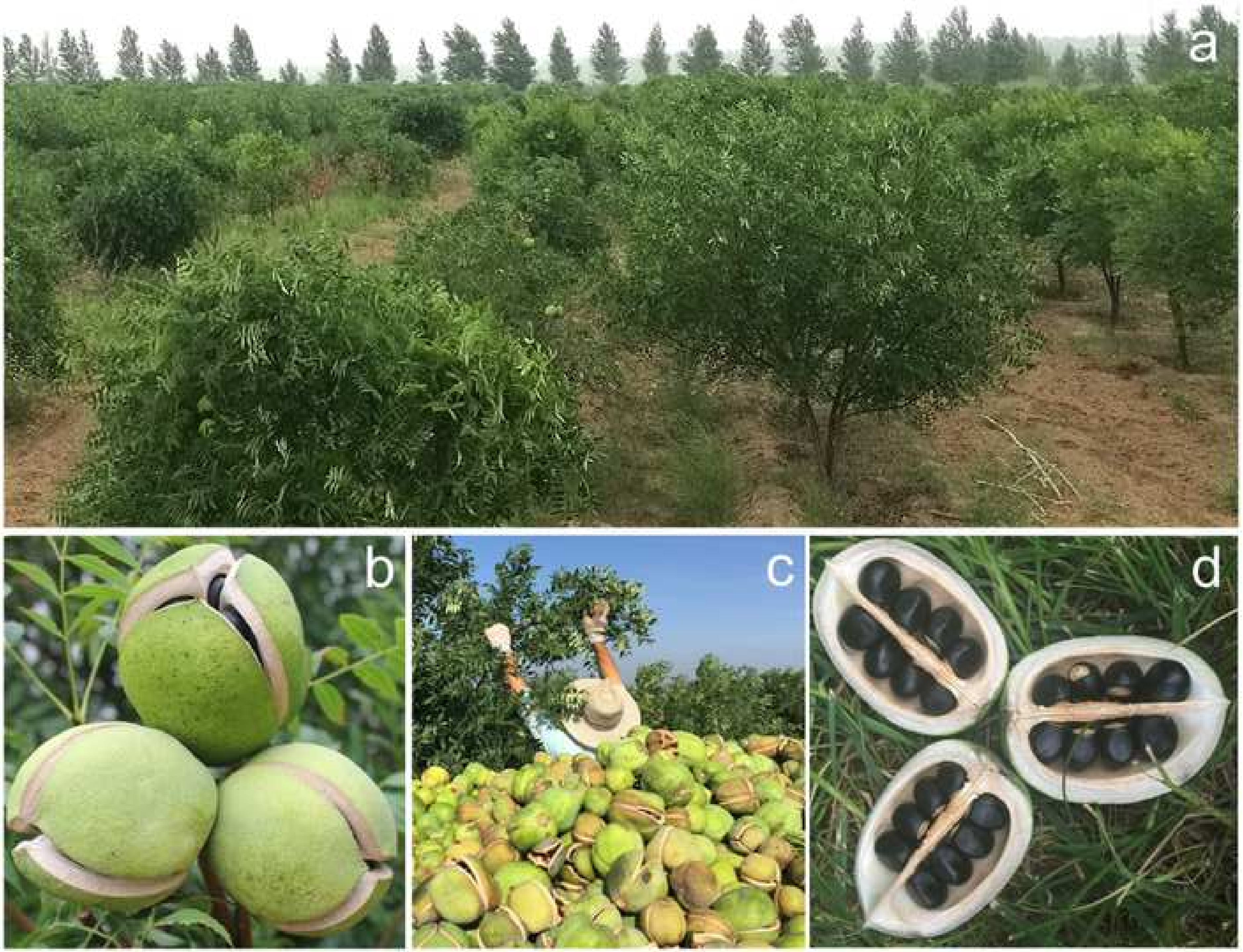

Figure 2

Click here to access/download;Figure;Fig.2.tif

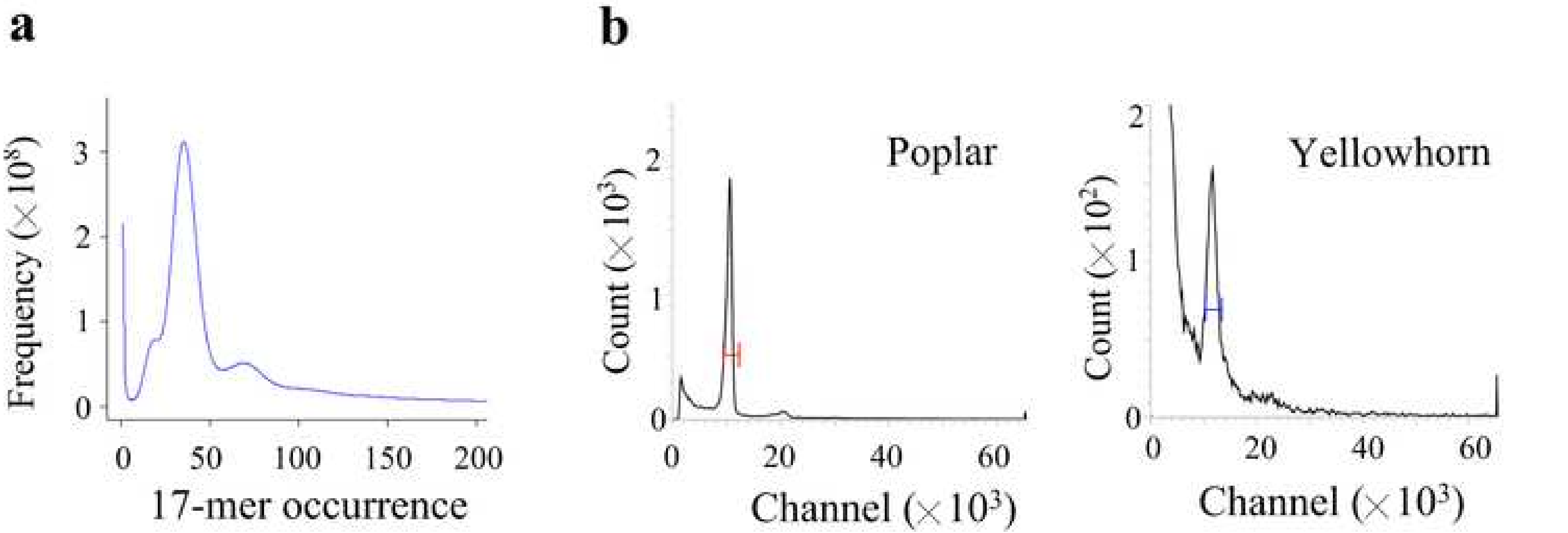

Figure 3 Click here to access/download;Figure;Fig.3.tif

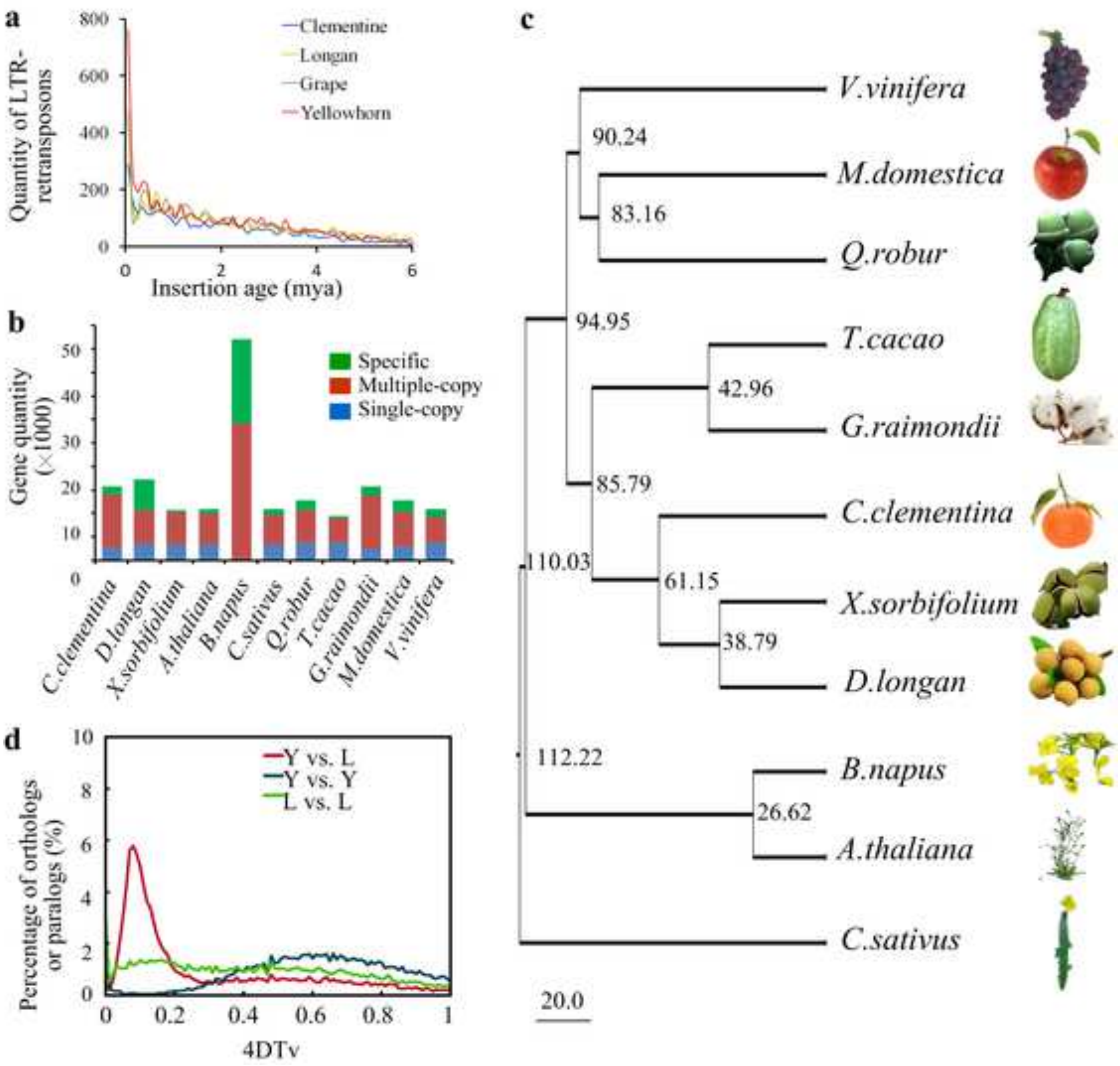

Figure 4 Click here to access/download;Figure;Fig.4.tif

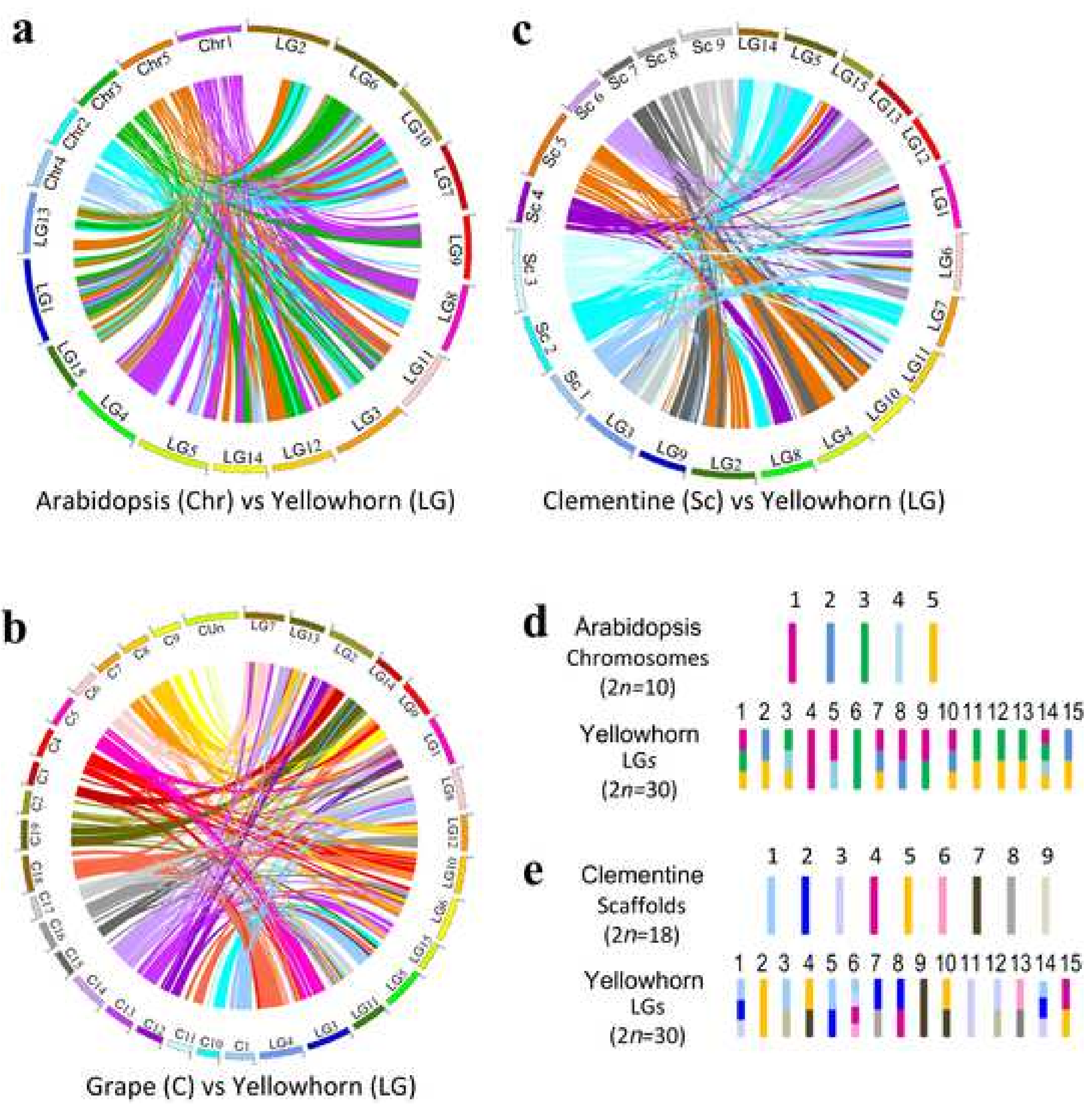

Supplementary table S1-5

Click here to access/download
Supplementary Material
Tables_AdditionalFiles_1.docx

Supplementary fig. S1-4

Click here to access/download
Supplementary Material
Figures_AdditionalFiles_2.docx

Supplementary supporting data

Click here to access/download
Supplementary Material
Figures_supporting_data.xlsx

Personal Cover Click here to access/download;Personal Cover;Response.pdf

Dear editors,

Thank you for consideration of our manuscript "Reference genome of a Chinese yellowhorn *Xanthoceras sorbifolium* provides insights into its conservation of original chromosomes". We have revised the manuscript according to the comments and completed the submission of the manuscript (GIGA-D-18-00337). The revisions are listed below.

Comment 1: Please provide more methodological detail as these sections are short and lack detail. E.g. what Hiseq did you use? What version of BUSCO did you use (should be v3 to be up to date). We recommend putting the protocols into to protocols.io, an, and have a list of protocols that may be relevant or can be easily adapted:https://www.protocols.io/groups/gigascience-journal.

Response: We have checked the methodological detail and confirmed the version of all software and database we used. The added informationhave highlighted in red and listed as follows: Jellyfish, BDFACSDiva in p5, LoRDEC, CD-HIT in p7, CEGMA, BUSCO in p8, Rfam, miRbase, GeneWise, OrthoMCL in p10, PHYML, Muscle, Gblocks, MCMCtree, MCscan in p11. We have added one reference in p8 as the 27th reference and revised the divergence time of yellowhorn and its close sister species longan (*Dimocarpus Longan*) in p2 and p11.

The used Illumina platform is Hiseq X Ten. The BUSCO test is performed using the website serverat https://gvolante.riken.jp/analysis.html.The corresponding information has been added into the section of genome sequencing and assembly in the maintext (Table S2 of additional file 1).

Comment 2: Please double check the accuracy of the results, particularly the phylogenies. This seems to lack an outgroup when reconstructed and the display of time tree was wrong, The age should also be marked on node, rather than the relative age of a branch.

Response: We have checked the results and reconstruct the phylogenetic tree using the grape as an outgroup, on which divergence times are marked on nodes (figure 3C).

Comment3: We require all the data to be available for scrutiny by the referees. The raw data needs to be in the SRA (or have referee access), both for the genomic and transcriptomic data (inc. the Hi-C). Processed data inc. assemblies, annotations, results and custom code should also be copied to our FTP servers and I ccour curators to help you copy this over.

Response: The raw sequence data have been deposited in NCBI under project accession number PRJNA483857(https://www.ncbi.nlm.nih.gov/search/?term=PRJNA483857).The Short Read Archive (SRA) accession number was SRP159119 (SRR7768197, SRR7768198, SRR7768199, SRR7768201), The accession number of *Xanthoceras sorbifolium* Genome sequencing and assembly was QUWJ 00000000. All the data are available for download.

If you have any question about this paper, please don't hesitate to let us know.

Sincerely yours

Libing Wang
