# Supplementary material for: Pseudomolecule-level assembly of the Chinese oil tree yellowhorn (Xanthoceras sorbifolium) genome
Source: Gigascience. 2019 Jun 26;8(6):giz070. doi: 10.1093/gigascience/giz070 (PMC6593361; doi:10.1093/gigascience/giz070)
Supplement: giz070_GIGA-D-18-00337_Revision_1 [file giz070_giga-d-18-00337_revision_1.pdf]

GigaScience

Pseudomolecules-level assembly of a Chinese oil tree yellowhorn (Xanthoceras sorbifolium) genome

--Manuscript Draft--

**Manuscript Number:**	GIGA-D-18-00337R1
**Full Title:**	Pseudomolecules-level assembly of a Chinese oil tree yellowhorn (Xanthoceras sorbifolium) genome
**Article Type:**	Data Note
**Funding Information:**	the Central Public-Interest Scientific Institution Basal Research Fund (CAFYBB2017QB001, CAFYBB2019QD001) — Dr. Libing Wang<br>the National "12th Five-Year" Plan for Science & Technology Support of China (2015BAD07B0106) — Dr. Chengjiang Ruan<br>Major Research Plan (31800571, 31870594, 31760213) — Dr. Libing Wang<br>National Key Research and Development Plan of China (2016YFC050080506) — Dr. Libing Wang
**Abstract:**	Backgrounds: Yellowhorn (Xanthoceras sorbifolium) (NCBI Taxonomy ID: 99658) is a species of the Sapindaceae family in China. It is an oil tree that can withstand cold and drought conditions. Pseudomolecules-level genome assembly will not only contribute to understanding the evolution of genes and chromosomes, but also bring yellowhorn breeding into a genomic era.<br>Findings: Here we generated 15 pseudomolecules of the yellowhorn chromosomes, on which 97.04% of scaffolds anchored, using the combined technologies of Illmina HiSeq, Pacbio sequel and Hi-C. Length of the final genome assemblies of yellowhorn is 504.2 Mb with a contig N50 size of 1.04 Mb and a scaffold N50 size of 32.17 Mb. Genome annotation revealed that 68.67% of the yellowhorn genome was composed of repetitive elements. Gene modeling predicted 24,672 protein-coding genes. Comparison of the identified orthologous genes estimated the divergence time of yellowhorn and its close sister species longan (Dimocarpus Longan) approximately at 33.07 million year ago (mya). Gene clusters and chromosome synteny demonstrated that the yellowhorn genome conserved the genome structure of its ancestor in some chromosomes.<br>Conclusion: This genome assembly presented a high quality reference genome of yellowhorn. Integrated genome annotations provided a valuable data set for genetic and molecular research in this species. We did not detect whole-genome duplication in this genome. The yellowhorn genome carried the syntenic blocks of its ancient chromosomes. All of these data sources will enable this genome to serve as an initial platform for breeding better yellowhorn.
**Corresponding Author:**	Libing Wang, Ph.D.<br><br>CHINA
**Corresponding Author Secondary Information:**	
**Corresponding Author's Institution:**	
**Corresponding Author's Secondary Institution:**	
**First Author:**	Quanxin Bi
**First Author Secondary Information:**	
**Order of Authors:**	Quanxin Bi<br>Yang Zhao

	Wei Du
	Ying Lu
	Lang Gui
	Zhimin Zheng
	Haiyan Yu
	Yifan Cui
	Zhi liu
	Tianpeng Cui
	Deshi Cui
	Xiaojuan Liu
	Yingchao Li
	Siqi Fan
	Xiaoyu Hu
	Guanghui Fu
	Jian Ding
	Chengjiang Ruan
	Libing Wang, Ph.D.
**Order of Authors Secondary Information:**	
**Response to Reviewers:**	See the personal cover letter in the last section of the GIGA-D-18-00337_R1
**Additional Information:**	
**Question**	**Response**
Are you submitting this manuscript to a special series or article collection?	No
**Experimental design and statistics**<br><br>Full details of the experimental design and statistical methods used should be given in the Methods section, as detailed in our Minimum Standards Reporting Checklist. Information essential to interpreting the data presented should be made available in the figure legends.<br><br>Have you included all the information requested in your manuscript?	Yes
**Resources**<br><br>A description of all resources used, including antibodies, cell lines, animals and software tools, with enough	Yes

Powered by Editorial Manager® and ProduXion Manager® from Aries Systems Corporation

information to allow them to be uniquely identified, should be included in the Methods section. Authors are strongly encouraged to cite Research Resource Identifiers (RRIDs) for antibodies, model organisms and tools, where possible.<br><br>Have you included the information requested as detailed in our Minimum Standards Reporting Checklist?	
**Availability of data and materials**<br><br>All datasets and code on which the conclusions of the paper rely must be either included in your submission or deposited in publicly available repositories (where available and ethically appropriate), referencing such data using a unique identifier in the references and in the “Availability of Data and Materials” section of your manuscript.<br><br>Have you have met the above requirement as detailed in our Minimum Standards Reporting Checklist?	Yes

Powered by Editorial Manager® and ProduXion Manager® from Aries Systems Corporation

Revisied Manuscript
Click here to view linked References
Click here to access/download;Manuscript;revision-MS_yellowhorn_maintext20190302.docx

Pseudomolecules-level assembly of a Chinese oil tree yellowhorn (*Xanthoceras sorbifolium*) genome

Quanxin Bi[1,2,†], Yang Zhao[1,†], Wei Du[2,†], Ying Lu[3], Lang Gui[3], Zhimin Zheng[4,5], Haiyan Yu[1,6], Yifan Cui[1], Zhi Liu[4,5],Tianpeng Cui[7], Deshi Cui[7], Xiaojuan Liu[1], Yingchao Li[1], Siqi Fan[1], Xiaoyu Hu[1], Guanghui Fu[1], Jian Ding[2], Chengjiang Ruan[2,]*, Libing Wang[1,]*

[1] State Key Laboratory of Tree Genetics and Breeding, Research Institute of Forestry, Chinese Academy of Forestry, Beijing 100091, China.

[2] Key Laboratory of Biotechnology and Bioresources Utilization, State Ethnic Affairs Commission & Ministry of Education, Dalian Minzu University, Dalian 116600, China.

[3] National Demonstration Center for Experimental Fisheries Science Education, Key Laboratory of Exploration and Utilization of Aquatic Genetic Resources (Ministry of Education) and International Research Center for Marine Biosciences (Ministry of Science and Technology), Shanghai Ocean University, Shanghai 201306, China.

[4] State Key Laboratory of Tree Genetics and Breeding, Northeast Forestry University, Harbin 150040, China.

[5] Key Laboratory of Saline-alkali Vegetation Ecology Restoration (SAVER), Ministry of Education, Alkali Soil Natural Environmental Science Center (ASNESC), Northeast Forestry University, Harbin, China

[6] Beijing ABT Biotechnology Co., Ltd., Beijing 102200, China.

[7] Zhangwu Deya yellowhorn Professional Cooperatives, Zhangwu 123200, China.

*Correspondence address: Libing Wang, State Key Laboratory of Tree Genetics and Breeding, Research Institute of Forestry, Chinese Academy of Forestry, Beijing 100091, China; E-mail: wlibing@caf.ac.cn; Chengjiang Ruan, Key Laboratory of Biotechnology and Bioresources Utilization, State Ethnic Affairs Commission & Ministry of Education, Dalian Minzu University, Dalian 116600, China; E-mail: ruan@dlnu.edu.cn.

[†]These authors contributed equally to this article.

Abstract

Backgrounds: Yellowhorn (*Xanthoceras sorbifolium*) (NCBI Taxonomy ID: 99658) is a species of the Sapindaceae family in China. It is an oil tree that can withstand cold and drought conditions. Pseudomolecules-level genome assembly will not only contribute to understanding the evolution of genes and chromosomes, but also bring yellowhorn breeding into a genomic era.

Findings: Here we generated 15 pseudomolecules of the yellowhorn chromosomes, on which 97.04% of scaffolds anchored, using the combined technologies of Illmina HiSeq, Pacbio sequel and Hi-C. Length of the final genome assemblies of yellowhorn is 504.2 Mb with a contig N50 size of 1.04 Mb and a scaffold N50 size of 32.17 Mb. Genome annotation revealed that 68.67% of the yellowhorn genome was composed of repetitive elements. Gene modeling predicted 24,672 protein-coding genes. Comparison of the identified orthologous genes estimated the divergence time of yellowhorn and its close sister species longan (*Dimocarpus Longan*) approximately at 33.07 million year ago (mya). Gene clusters and chromosome synteny demonstrated that the yellowhorn genome conserved the genome structure of its ancestor in some chromosomes.

Conclusion: This genome assembly presented a high quality reference genome of yellowhorn. Integrated genome annotations provided a valuable data set for genetic and molecular research in this species. We did not detect whole-genome duplication in this genome. The yellowhorn genome carried the syntenic blocks of its ancient chromosomes. All of these data sources will enable this genome to serve as an initial platform for breeding better yellowhorn.

Keywords

Xanthoceras sorbifolium, yellowhorn, PacBio sequencing, Genome assembly, Hi-C, Genome annotation, Conserved chromosome.

Data description

Background

Yellowhorn (*Xanthoceras sorbifolium*) is a woody oil-species [1], which belongs to the Sapindaceae family and the monotypic genus *Xanthoceras*. As an endemic and economic species in Northern China, it is widely used for conserving soil and water due to the capacity to survive in arid, saline, alkaline land and in extreme temperature even below −40 ℃ [2, 3]. There are almost 7.5×10^5 ton yellowhorn seeds are being harvested in autumn every year [4] (Fig.1). The oil content of its seed kernel could be as high as 67%, of which 85%-93% is unsaturated fatty acid, including 37.1%-46.2% linoleic acid and 28.6%-37.1% oleic acid, which are essential fatty acids in diets [5]. Recently, yellowhorn as one of the major woody oil plant species has drawn government and people's attention again for the shortage of vegetable oil resources in China. Notably, an essential nutrient for brain growth and maintenance—nervonic acid, which is rarely contained in plants, reach nearly 3.04% in the seed oil of yellowhorn [6, 7]. More latest results indicated that xanthoceraside, a novel triterpenoidsaponin extracted from the husks of yellow horn, had an effect of antitumor and the potential to treat Alzheimer's [8-10]. In this study, we present the high-quality yellowhorn genome and conduct the annotation and genomic structures, evolution. The data provide a rich resource of genetic information for developing these resources and understanding the special space of *Xanthoceras* and Sapindaceae in plant evolution.

Sequenced individual and sample collection

Tender leaves were collected from an individual *X. sorbifolium* cv. Zhongshi 4, that is a new variety issued by National Forestry and Grassland Administration (Variety rights No. 20180121) in Zhangwu, Liaoning, China. This tree was produced via clone of a plus tree from natural population in Tongliao, Inner Mongolia, China .The leaves were then frozen in liquid nitrogen and stored at -80℃ until DNA extraction.

Estimation of genome size through a flow cytometry analysis

The one-month-old leaves from the sequenced yellowhorn individual were put on a flow cytometry analysis to estimate genome size as mentioned by Galbraith [11]. The *Glycine max* Var. William 82 (2C genome size=2.28 pg) [12-13] and *Populus trichocarpa* Var. Nisqually 1 (2C genome size= 0.99 pg)[14] were used as a standard reference. The soybean and yellowhorn samples were co-chopped with an internal standard using a razor blade and stained nuclei with propidium iodide. To avoid the peaks too close to be distinguished when run simultaneously, the poplar and yellowhorn samples run separately. Each sample is measured three times on the flow cytometer. Over 3,000 nuclei were analyzed per sample with a FACSAria flow cytometer (Becton, Dickinson and Company). A total of 16 samples were analyzed using soybean and poplar as the standard species. The software BDFACSDiva (version 8.0.1) was used for data analysis with the coefficient variation controlled in 5%. Compared with the soybean internal standard (peak at 25, 413) and poplar reference (peak at 10,363) respectively, the peak values of the 16 yellowhorn samples' fluorescence intensity were 11,968 and 11,558. Referencing the soybean genome size (1,115 Mb) and poplar genome size (485±10 Mb) [13-15] , the yellowhorn genome size was estimated to be approximately 525.94 Mb and 540.93 Mb, which were relatively closed (**Fig.2a**).

Illumina short-read sequencing

DNA was extracted from the leaves of the same individual using DNA secure Plant Kit (TIANGEN, China). Concentration and quality was assessed by 1% agarose gel electrophoresis and 2.0 Flurometer (Life Technologies, CA, USA). One shotgun library with an insert size of 350 bp was prepared using NEB Next® Ultra DNA Library Prep Kit (NEB, USA). A total of 34.51 Gb raw sequencing data were generated by Illumina HiSeq X Ten sequencing platform, around 63.80-fold of the assembled genome. Primary data analysis was carried out using standard Illumina pipeline [16]. Short reads were processed with Trimmomatic (version 0.33) [17,18] and Cutadapt (version 1.13) [19] to remove adapter, leading and trailing bases with a quality score below 20, and reads with an average per-base-quality of 20 over

a 4 bp sliding window. The trimmed reads with the length less than 70 nucleotides were discarded. Finallly, 34.40 Gb clean reads were used for the following genomic survey and error collection of Pacbio reads.

Estimation of the genome size by a *K*-mer analysis.

A *K*-mer analysis was performed to estimate the genome size as mentioned by Marçais [20]. All the generated Illumina reads were applied to 17-mer counting using Jellyfish (v2.1.1) with the parameters -m 17 -t 10 –s 550M. The *K*-mer depth of coverage follows the Poisson distribution [21], and the mean *K* -mer depth should be equal to the peak value of the *K*-mer depth distribution. The *K*-mer analysis indicated quantity of the total *K* -mers (*K* = 17) at 18,458, 632, 032 and frequency of 17-mers at 34 × depth **(Fig.2b)**. As mentioned by Varsheny [22], using the following formula: Genome size = *K* -mernum / Peak depth, the genome size was estimated to be approximately 542.90 Mb **(Fig.2b)**. Revised genome size was 536.58 Mb that generally agreed with the estimates with the flow cytometry.

PacBio SMRT sequencing

Genomic DNA (gDNA) was extracted following ~40 kb SMRTbell™ Libraries Protocol (https://www.pacb.com/wp-content/uploads/Procedure-Checklist-Preparing-Greater-Than-30-kb-SMRTbell-Libraries-Using-Needle-Shearing-and-BluePippin-Size-Selection-on-Sequel-and-RSII-Systems.pdf). DNA was purified with Mobio PowerClean® Pro DNA Clean-Up Kit and quality was assessed by standard agarose gel electrophoresis and Thermo Fisher Scientific Qubit Fluorometry. Genomic DNA was sheared to a size range about 40 kb using g-TUBE (Covaris) and 0.45 × AMPure beads were used to enrich and purify large fragments of DNA. Damaged DNA and ends were enzymatically repaired as recommended by Pacific Biosciences. Following this procedure, hairpin adapters were ligated by blunt-end ligation reaction. The remaining damaged DNA fragments and those fragments without adapters at both ends were digested using exonuclease. Subsequently, the resulting SMRTbell templates were purified by Blue Pippin electrophoresis (Sage Sciences) and sequenced on a PacBio RS II instrument using P6-C4 sequencing chemistry. A

primary filtering analysis was performed on the sequencer, and the secondary analysis was performed utilizing the SMRT analysis pipeline version 2.1.0 (Pacific Biosciences). In total, we generated 66.44 Gb (roughly 122.83-fold of the yellowhorn genome) of single-molecule sequencing data (6,105,692 PacBio post-filtered reads), with an average read length of 10,882 bp (**Fig.S1; Table S1**).

Genome assembly

After stringent filtering and correction steps using K-mer frequency-based methods [23], we assembled the contigs using the Pacbio reads. Preliminary assemblies with assembler Falcon v0.7 (https://github.com/PacificBiosciences/FALCON/wiki/Manual) (falcon_sense_option = --output_multi --min_idt 0.70 --min_cov 4 --max_n_read 300 --n_core 8 overlap_filtering_setting = --max_diff 100 --max_cov 100 --min_cov 2 --n_core 12 --bestn 10) generated a total length of 598.65 Mb of contigs with a N50 length of 1.11 Mb, using the 66.44 Gb PacBio long reads. The software Quiver (based on pbsmrtpipe.pipelines.sa3_ds_resequencing in smrtlink_5.0.1; http://pbsmrtpipe.readthedocs.io/en/master/getting_started.html) is used to polish the Pacbio consensus sequence clusters. The assemblies were corrected by the Pilon (version 1.22) (https://github.com/broadinstitute/pilon/wiki) using the Illumina short reads. Finally, the heterozygous sequences were identified and removed basing Purge Haplotigs pepline, with parameters -a 75 (https://bitbucket.org/mroachawri/purge_haplotigs) [24]. Also, contigs from organelle DNA sources can be identified and filtered when the processing with Purge Haplotigs. After the heterozygous sequences were removed, the final assemblies from Pacbio reads (504.20 Mb) were generated (**Table 1**).

Pseudomolecules construction and three-dimensional chromatin conformation analysis

Hi-C technology is an efficient strategy for pseudomolecule construction and enables the generation of genome-wide three-dimensional architecture of chromosomes. We constructed Hi-C fragment libraries of 350 bp and sequenced them using the Illumina Hi-seq platform (Illumina, San Diego, CA, USA) for chromosome pseudomolecule construction.

Mapping of the Hi-C reads and assignment to restriction fragments were performed as described in Burton [26]. A total of 53.39 Gb of trimmed reads, accounting for around 98.70-fold coverage of the yellowhorn genome, were mapped to the assemblies with aligner BWA (version 0.7.10) (parameters: bwa index -a bwtsw fasta bwa aln -M 3 -O 11 -E 4 -t 2 fq1 bwa aln -M 3 -O 11 -E 4 -t 2 fq2) [27]. Only uniquely-aligned reads with high alignment quality (>20) were selected for the construction of the pseudomolecules. Duplicate removal and quality assessment were performed using HiC-Pro (version 2.8.1) with parameters: mapped_2hic_fragments.py -v -S -s 100 -l 1000 -a -f -r -o [28]. The 50.56% of Hi-C data were grouped into the valid interaction pairs. A total of 2,836 contigs (N50 length at 1.04 Mb) were assembled after error correction. LACHESIS (parameters: cluster_min_re_sites=48; cluster_max_link_density=2; cluster_noninformative_ratio =2; order_min_n_res_in_trun=14; order_min_n_res_in_shreds=15) [26] was used to assign the order and orientation of each group, with a scaffold N50 of 32.17 Mb.

Using the 98.70-fold coverage of Hi-C reads, 489.28 Mb (97.04%) of the assemblies were anchored onto the 15 pseudomolecules, of which are in agreement with Karyotype (2n=30) of yellowhorn identified by Li [25]. The assemblies (477.59 Mb, 94.76%) were ordered by frequency distribution of valid interaction pairs (**Table 2, Fig.S2**). And the coverage of assembly reached 93.96% and ratio of unclosed gap was 0.15‰(Table1). The assemblies have a high quality to be used as a reference for study of yellowhorn biology and plant genomics.

SNP calling

SNP calling were used to estimate the heterozygosity rates. The raw Illumina reads were aligned to the reference genome using Bowtie 2.2.5 with default parameters. The alignments were converted to BAM format and duplicated reads were removed with samtools. SNPs were called with Gatk 2.8.1 protocol to produce a VCF file. And there were 1,499,418 heterozygosity SNPs (Additional file Table S8) were identified. The heterozygosity rates were calculated as: heterozygosity SNPs/ genome_size, which is 0.30%.

Transcriptome sequencing

RNA was extracted from four tissues, flowers, leaves, stems and roots of the same individuals as the DNA sequencing using the easy spin RNA extraction kit (Sangon Biotech, Shanghai, China; No. SK8631). The concentration of each RNA sample was checked using NanoDrop (Thermo Fisher Scientific Inc., USA) and the QUBIT ® Fluorometer (Life Technologies). The RNA integrity was checked using a Bioanalyzer 2100 (Agilent Technologies). The Iso-Seq libraries were prepared according to the Isoform Sequencing protocol (Iso-Seq) using the Clontech SMARTer PCR cDNA Synthesis Kit and the BluePippin Size Selection System protocol as described by Pacific Biosciences (PN 100-092-800-03). Mixed Sample was sequenced using on the Pacific Biosciences RS II platform with one SMRT cell v3 each based on P6-C4 chemistry.

Sequence data were processed using the SMRTlink 4.0 software. Circular consensus sequences were derived from the subread BAM files with the parameters: min_length 200, max_drop_fraction 0.8, no_polish TRUE, min_zscore -999, min_passes 1, min_predicted_accuracy 0.8, max_length 18000. Separation of the full length and non-full length reads were conducted using pbclassify.py (ignorepolyA false, minSeqLength 200). Non-full length and full-length fasta files produced were then fed into the cluster step to cluster the isoforms, followed by final Arrow polishing with the parameters of hq_quiver_min_accuracy 0.99, bin_by_primer false, bin_size_kb 1, qv_trim_5p 100, qv_trim_3p 30. The LoRDEC software (version 0.3) was used to correct sequencing errors in the consensus transcripts using Illumina reads as the reference (parameters: -k 19 -s 3) [29]. The corrected consensus transcripts were clustered using CD-HIT (version 4.6.8) (-c 0.99 -T 6 -G 0 -aL 0.90 -AL 100 -aS 0.99 -AS 30) [30] to reduce sequences redundancy and improve the performance of other sequence analyses.

A total of 110,584 non-redundant unigenes were generated from 142,396 transcripts in the final RNA assemblies, which were used as evidence to assist the gene prediction. Among the 110,584 non-redundant transcripts, 8,466 (7.66%) are non-coding mRNAs. And each gene has average 2-7 transcripts, of which the longest transcript representing that gene is kept in the final gene model set.

Evaluation of assembly quality

Completeness of the final assemblies was evaluated using CEGMA (version 2.5) [31] (http://korflab.ucdavis.edu/dataseda/) and BUSCO (version 3.0.2) [32-33] (https://gvolante.riken.jp/analysis.html), respectively. The CEGMA outputs display a 94.76% of core eukaryotic genes (235 out of 248 core eukaryotic genes) in our assemblies. The BUSCO test, referencing the embryophyta protein set (run_BUSCO.py -i plant_species.fa -o plant_species-l embryophyta_odb9/-m proteins), exhibit that 94.7% of plant gene sets were identified to be complete (1364 out of 1440 BUSCOs), including 89.0% single-copy and 5.7% duplicated genes (**Table S2**). All of these results support the high assemble quality of the yellowhorn genome.

Annotation of the repetitive sequences

A *de novo* repeat database was constructed using RepeatScout (version 1.0.5) [34], LTR-FINDER (version 1.0.7) [35], MITE-Hunter (version 1.0) [36] and PILER (version 1.0) with default parameters [37]. The predicted repeats were classified using PASTEClassifier (version 1.0) with default parameters [38-39]. Then, RepeatMasker (version 4.0.7) [40] was utilized with following parameters "-nolow -no_is -norna -engine wublast -qq -frag 20000" to identify repeat sequences by aligning them against the known gene and genome sequences, basing on Repbase (version 19.06) [41] and *de novo* repeat database.

The predicted repeats occupied 346.39 Mb (68.67%) of the yellowhorn genome assemblies. Of these repeats, two types of the LTR-retrotransposons are the most abundant, 98.68 Mb of *Copia*-type (19.57%) and 88.24 Mb of *Gypsy*-type (17.50%) (**Table S3**). Accumulation of LTR-retrotransposons is an important contributor to genome expansion and diversity [42]. The insertion time of the LTR-retrotransposons in the genomes is estimated by calculation of sequence variance between the LTR arms of each LTR-retrotransposon, utilizing the substitution rate of 1.3×10^{-8} substitutions per site per year [43].To calculate the insertion age of each LTR retrotransposons, 5'and 3' LTRs of the each element were aligned with MUSCLE (version 3.8.31) with default setting parameters [44]

(https://www.ebi.ac.uk/Tools/msa/muscle/). Distmat (with default parameters) was used to estimate the DNA divergence between the LTR sequences with the Kimura-2-parameter base substitution Model [45] and DNA divergence was converted to divergence time. A comparison of the insertion ages for the LTR-retrotransposons illustrated a similar insertion profiles among the genomes of clementine [46] (annotation version 1.0), longan [47] (annotation version 1.0), grape [48] (*V. vinifera*, annotation version GenomeScope.12X) and yellowhorn **(Fig. 3a)**. We observed that the yellowhorn genome carried more young LTR-retrotransposons, which were accounted for the highest proportion with insertion ages less than 0.2 mya. This might be resulted from the rapid changes of the growing environment, such as the effect from pathogens and the interference with human activities in the recent years. The genomes sequenced by pure next-generation sequencing technology might lose more LTR-retrotransposons because the sequencing similarity between LTR arms and among different LTR-retrotransposons probably caused the assembly errors of these regions, which may have led to an under-estimation of the LTR-retrotransposons in clementine and longan. Comparison of the insertion ages suggested a similar insertion age between *Copia*-type and *Gypsy*-type LTR-retrotransposons (Fig.S5).

Prediction of protein-coding genes

Annotation of protein-coding genes in the yellowhorn genome was conducted by combining *de novo* prediction, homology information, and RNA-seq data. For the *de novo* prediction, Genscan (version 3.1) [49], Augustus (version 3.1) [50], GlimmerHMM (version 3.0.4) [51], GeneID (version 1.4) [52], SNAP (version 2006-07-28) [53] were used on the repeat masked genome with default parameters. For the similarity-based prediction, the Uniprot protein sequences from the three sequenced plants, Arabidopsis (TAIR 10, http://brassicadb.org/brad/datasets/pub/BrassicaceaeGenome/ Arabidopsis_thaliana/), longan (V1.0, http://gigadb.org/dataset/100276) and grape (Genomescope 12×, https://www.ncbi.nlm.nih.gov/genome/?term=Vitis+vinifera+genome), were aligned against the *ab initio* gene models in the yellowhorn genome using GeMoMa (version 1.3.1) [54]. When the multiple transcripts predicted at the same

location, the best GeMoMa scoring transcript was chosen as the optimal model [55]. The RNA-seq data were aligned to the reference genome with PASA (version 2.0.2) [56] under default parameters. All predictions from the three methods were combined with EVidenceModeler (v1.1.1) (Mode:STANDARD S-ratio: 1.13 score>1000) [57] to produce a consensus gene set. During the EVM integration, higher weights were assigned to the predicted PASA and GeMoMa models than the *ab initio* models. The PASA was used to modify the final gene model.

The RNA-seq reads were then aligned to the yellowhorn genome assemblies with TopHat (v2.0.10, implemented with bowtie2) [58] to identify candidate exon regions and splicing donor and acceptor sites to evaluate the results of gene prediction. Infernal (version 1.1) (default parameters) [59] was used to identify the non-coding mRNA genes of rRNA and microRNA based on Rfam (version 12.1) [60] and miRbase (version 21) [61]. TRNAscan-SE (version 1.3.1) (default parameters) [62] was used to identify the tRNA genes.

GenBlastA v1.0.4(-e 1e-5) was used to perform pseudogene prediction by scanning the yellowhorn genome for sequences homologous to the known protein-coding genes it contained, and premature stop codons or frame shift mutations in those sequences were searched by GeneWise (version 2.4.1) with parameters: -both -pseudo [63-64].

Functional annotation of the protein-coding genes was carried out by the alignment of the NR, KOG, GO, KEGG, TrEMBL database. Besides, the gene models were aligned to Pfam database using Hmmer (version 3.0) (parameters, -E 0.00001 --domE 0.00001 --cpu 2 --noali –acc)] [64-70]. GO terms were allocated to the genes using Blast2GO (version2.2.31) pipeline [70].

In total, we predicted 24,672 protein-coding genes (**Table S4**) and 1,913 Pseudogenes, with the average gene length of 4,199 bp, average intron length of 2,560 bp and average coding sequence length of 1,580 bp. Of these genes, 99.02% (24,429) carried at least one conserved functional domain (**Table S5**). Their functions were classified by GO terms (**Fig. S3**) and KOG database (**Fig. S4**). For the non-coding mRNA genes, 642 tRNA, 108 microRNA and 316 rRNA genes were predicted in the yellowhorn genome.

Chromosome synteny between yellowhorn and the reference genomes

To investigate evolution of the yellowhorn chromosomes, the gene collinearity is constructed by anchoring the aligned yellowhorn genes on the reference genomes, clementine, Arabidopsis and grape, respectively, using the Mutilple Collinearity Scan toolkit (MCscan) (version 0.8) [71]. The parameter of MCscan alignment was as follows: $/MCScanX xxx.blast$—s 10 -—b $2（inter-species）blastp -query b.fa -db adb -out xyz.blast -evalue 1e-10 -num_threads 16 -outfmt 6 -num_alignments 5. A total of 367, 409 and 386 syntenic blocks were identified on the basis of the orthologous gene orders, corresponding to 28,372, 18,650 and 23,400 genes in each genome, respectively. Correspondingly, average gene number per each block was 77.3, 45.6 and 60.6 genes, respectively. This suggested the highest collinearity between yellowhorn and clementine, which was consistent to their Sapindale clade of the phylogenetic relationship. Alignments of syntenic chromosomes were visualized between yellowhorn and other genomes. Frequency of the large-scale fragment rearrangements between yellowhorn and clementine, including inversions and translocations, displayed considerably lower than the other two (**Fig. 4**). Especially, structural variation between yellowhorn and grape was so frequent that it is too difficult to speculate the syntenic relationship among the chromosomes (**Fig. 4b**). The concluded chromosome alignments between yellowhorn linkage groups and clementine pseudomolecules revealed that most of cross-chromosome rearrangements were different from that between yellowhorn and Arabidopsis (**Fig. 4d, 4e**). It was found that yellowhorn Linkage group 2 and 11 are syntenic to a single clementine pseudomolecules, Scaffold 5 and 3, respectively, and the Linkage groups 3, 4, 5, 7, 8, 10, 12, 14 and 15 were aligned to two reference chromosomes of clementine. Comparatively, frequency of chromosome rearrangement was a little higher between yellowhorn linkage groups and Arabidopsis chromosomes. The Arabidopsis Chromosome 1 is predominantly syntenic to yellowhorn Linkage group 4, which demonstrated that the yellowhorn genomes conserved some genome structure of its originals (**Fig. 4d**). Intriguingly, the similar chromosomal fusion events were found among some chromosomes. Aligned fragments of Arabidopsis Chromosomes 1, 3 and 5 fused to form yellowhorn linkage groups 1 and 14, which was the same as clementine Scaffolds 1, 2 and 3. Yellowhorn Linkage group 6 was aligned to clementine scaffolds 1, 3, 4 and 6,

but had extensive collinearity with Arabidopsis Chromosome 3 (**Fig. 4d, 4e**). However, phylogenetic analysis suggested a distant relationship between Arabidopsis and yellowhorn. These findings speculated that Arabidopsis and yellowhorn share a chromosome of their origins, despite of the extensive rearrangements. In general, these findings shed new light on the evolution of endicot plant chromosome.

Identification of gene clusters and duplication

Gene clustering was conducted using OrthoMCL (version 5, parameters: Pep_length 10 Stop_coden 20 PercentMatchCutoff 50 EvalueExponentCutoff -5 Mcl 1.5 #1.2~4.0) [72] among the protein sequences of ten high quality typical endicot genomes representative of *D. Longan* (Sapindaceae, Sapindales) [46], *Citrus clementina* (Rutaceae, Sapindales) [47], *Brassica rapa* (Brassicaceae, Brassicales), *Arabidopsis thaliana* (Brassicaceae, Brassicales) [73-74], *Theobroma cacao* (Sterculiaceae, Malvales) [75], *Gossypium raimondii* (Malvaceae, Malvales) [76], *Quercus robur* (Fagaceae, Fagales) [77], *Vitis vinifera* (Vitaceae, Vitales) [78], *Cucumis sativus* (Cucurbitaceae, Cucurbitales) [79] and *Malus* × *domestica* (Rosaceae, Rosales) [80] families, as well as yellowhorn (Additional file: Table S6). The yellowhorn genes were clustered into a total of 14,828 families, including 169 yellowhorn-specific gene families (Additional file: Table S7). Comparison of gene copy numbers among eleven endicot genomes indicated that the yellowhorn genome had the similar proportion of the single and multiple copy genes with other analyzed genomes (**Fig. 3b**). Intriguingly, the species-specific genes of yellowhorn were similar to *T. cacao*, which implicated that the yellowhorn genes might conserve the similar gene structure with their origins.

A total of 300+ one-to-one single-copy gene shared by all of the used eleven genomes were identified to construct the phylogenetic tree using PHYML (version 3.0) (**Fig. 3c**) [81]. The model of TIM2+I+G determined by the jmodeltest was used to construct the evolution tree. The Software Muscle (version 3.8.31) (https://www.ebi.ac.uk/Tools/msa/muscle/) [44] was used to align the orthologs. Alignment outputs were treated with Gblocks (version 14.1) with the parameters of -t = p -b5 = h -b4 = 5 -b3 = 15 -d = y -n= y [82]. The divergence times

were estimated using MCMCtree (version 4.7a) (http://abacus.gene.ucl.ac.uk/software/paml.html) [83]with the parameters: burn-in=10,000，sample-number=100,000，sample-frequency=2. The TimeTree database (http://www.timetree.org/), r8s (parameter: r8s -b -f r8s_in.txt > r8s_out.txt) and divergence time (Whelan [84] and Yang [85]) were used for calibrating the time. Calibration time of fossils used in evolutionary trees is as follows: (((Qrob,(Csat,Mdom)),((Ccle,(Xsor,Dlon)),((Tcac,Grai),(Brapa,Atha)'<30.9>20.4'))),Vvin)'<115>105' . The credibility intervals for the divergence time estimates was as follows: UTREE 1 = (((Qrob: 93.929608, (Csat: 83.608799, Mdom: 83.608799) [&95%={67.268, 96.218}]: 10.320809) [&95%={78.104, 105.034}]: 9.748170, ((Ccle: 64.380901, (Xsor: 33.069679, Dlon: 33.069679) [&95%={18.376, 48.565}]: 31.311222) [&95%={46.354, 81.164}]: 27.870851, ((Tcac: 38.243394, Grai: 38.243394) [&95%={21.870, 56.407}]: 43.965024, (Brapa: 26.409279, Atha: 26.409279) [&95%={20.721, 30.886}]: 55.799139) [&95%={67.279, 94.364}]: 10.043334) [&95%={77.382, 103.299}]: 11.426026) [&95%={89.679, 113.000}]: 6.145826, Vvin: 109.823604) [&95%={104.966, 114.982}]. In Sapindaceae family, yellowhorn and longan are indicative of the closest relationship, with the divergence time estimated at approximately 33.07 mya. Using the orthologous gene pairs of yellowhorn and longan identified by gene collinearity and paralogous pairs identified by gene cluster, the 4DTv (four-fold degenerate synonymous sites of the third codons) were calculated for all of these duplicated pairs. A species divergence peak (4DTv~0.1) was exhibited in yellowhorn vs. longan ortholog 4DTv distribution but no obvious peak could be seen in the yellowhorn paralog curve and longan paralog curve (**Fig. 3d**). The self-alignment of the chromosomes based on the identified gene synteny, no large-scale gene duplications can be found in the yellowhorn genome (**Fig. S2**), suggesting that the yellowhorn genome did not undergo the whole-genome and large-fragment duplication.

Abbreviations:

bp: base pair; BUSCO: Benchmarking Universal Single-Copy Ortholog; CDS: coding sequence; GO: Gene Ontology; kb: kilobases; KEGG: Kyoto Encyclopedia of Genes and Genomes; LTR: long terminal repeat; Mb: megabases; Mya: million years ago; NCBI: National Center for Biotechnology Information; PE: paired-end; RNA-Seq: RNAsequencing; SMRT: Single-Molecule Real-Time; SRA: Sequence Read Archive.

Additional File

Additional file 1: Tables S1 to S5

Table S1: Statistics of PacBio data.

Table S2: Genome quality assessed by the BUSCO test.

Table S3: Content of repetitive sequences.

Table S4: Prediction of protein-coding genes.

Table S5: Function annotation of the protein-coding genes.

Table S6: Data used in the orthoMCL analysis.

Table S7: Annotation and locus information of 172 yellowhorn-specific gene families.

Table S8: Results of SNP calling.

Table S9. The locus information of LTR-retrotransposons and yellowhorn-specific gene families.

Additional file 2: Figures S1 to S4

Figure S1: Length distribution of three types of the produced PacBio reads.

Figure S2: Interaction frequency distribution of Hi-C links among chromosomes.

Figure S3: Function classification of the protein-coding genes against the GO term database.

Figure S4: KOG function classification of the protein-coding genes.

Figure S5. Distribution of insertion ages of *Copia*-type and *Gypsy*-type LTR-retrotransposons.

Funding

This work was financially supported by the Central Public-Interest Scientific Institution Basal Research Fund (CAFYBB2017QB001, CAFYBB2019QD001), the National “12th Five-Year” Plan for Science & Technology Support of China (2015BAD07B0106), the National Natural Science Foundation of China (31800571, 31870594, 31760213), the National Key Research and Development Plan of China (2016YFC050080506) and the Key research and development plan of Liaoning Province.

Availability of supporting data

The raw sequence data have been deposited in NCBI under project accession number PRJNA483857, the Short Read Archive (SRA) accession number was SRP159119 (SRR7768197, SRR7768198, SRR7768199, SRR7768201), The accession number of *Xanthoceras sorbifolium* Genome sequencing and assembly was QUWJ 00000000. All supplementary figures and tables are provided in Additional Files.

Conflict of Interest

The authors declare that they have no competing financial interests.

Author Contributions

QXB, HYY, YL, CJR and LBW conceived and designed the study; TPC, XJL,YCL, SQF, XYH, GHF, YFC, JD, DSC, ZMZ and ZL prepared materials and conducted the experiments; QXB, YZ, WD, YL and LG wrote the manuscript.

Legend

Fig.1 Images of the yellowhorn plants. (**a**)The yellowhorn tree in artificial forest. (**b**)The ripe fruit, will dehisce in three parts by carpel. (**c**) A harvest scene of yellowhorn in northern China. (**d**) The seeds in the ripe fruits, which number is 18-24 in one fruit.

Fig.2 Estimation of the genome size. (**a**) Distribution of 17-mer frequency. Values for K-mers are plotted against the frequency (y axis) of their occurrence (x axis). The leftmost truncated peak at low occurrence (1-2) was mainly due to random base errors in the raw sequencing reads. (**b**) Test results of yellowhorn, poplar and yellowhorn & soybean samples using flow cytometry.

Fig.3 Genome evolution. (**a**) Distribution of insertion ages of LTR-retrotransposons. The *x*-axis represents the estimated insertion age (mya) of the LTR-retrotransposons. The *y*-axis represents the number of intact LTR-retrotransposons. (**b**) Comparison of copy numbers in gene clusters of analyzed endicot genomes. According to the identified gene clusters, the genes are grouped into single-copy, multiple-copy and species-specific (specific). (**c**) Constructed phylogenetic tree and divergence time estimation. The black numbers represent estimated divergence times (mya) which are measured by a bar of 20 million years, and green numbers represent bootstrap values. The grape (*V. vinifera*) is used as outgroup. (**d**) Genome duplication in endicot genomes as revealed through 4DTv analyses. The 4DTv of the orthologous pairs (Y vs. L) between yellowhorn (Y) and longan (L) and paralogous gene pairs within the yellowhorn (Y vs. Y) and longan genome (L vs. L) are plotted against their calculated 4DTv values.

Fig.4 Chromosome synteny. The circularized blocks represent the chromosomes of yellowhorn and the other genome. Aligned genes identified by the MCscanX are connected by the lines, of which the located chromosomes are shown in different colors. (**a**) Chromosome alignment of yellowhorn and Arabidopsis. (**b**) Chromosome alignment of yellowhorn and grape. (**c**) Chromosome alignment of yellowhorn and clementine. Colored ribbons connect the aligned genes. yellowhorn linkage groups are labeled as LG 1 to 15, Arabidopsis chromosomes labeled as Chr 1 to 5, grape chromosomes labeled as C1 to 19 and CUn (location of the chromosomes are unknown) and clementine labeled as Sc 1

to 9. Scale, 10Mb. (**d**) Chromosome rearrangement between Arabidopsis and yellowhorn. (**e**) Chromosome rearrangement between clementine and yellowhorn. Arabidopsis and clementine chromosomes are represented as the bars filled with different colors. Synteny and rearrangement of the yellowhorn chromosomes are indicated by different blocks, corresponding to referenced Arabidopsis and clementine chromosomes.

References

[1] Wang Q, Yang L, Ranjitkar S et al. Distribution and in situ conservation of a relic Chinese oil woody species yellowhorn *Xanthoceras sorbifolium* Bunge. Can J For Res 2017; 47: 1450-6.

[2] Board E. Flora of China vol.47. 1985; 47: 72.

[3] Yu HY, Fan SQ, Bi QX et al. Seed morphology, oil content and fatty acid composition variability assessment in yellow horn (*Xanthoceras sorbifolium* Bunge) germplasm for optimum biodiesel production. Ind Crop Prod 2017; 97: 425-30.

[4] Yao ZY, Qi JH, Yin LM, et al. Biodiesel production from *Xanthoceras sorbifolia* in China: Opportunities and challenges. Renew Sust Energy Rev 2013; 24: 57-65.

[5] Venegas-Calerón M, Ruíz-Méndez MV, Martínez-Force E et al. Characterization of *Xanthoceras sorbifolium* Bunge seeds: Lipids, proteins and saponins content. Ind Crop Prod 2017; 109: 192-8.

[6] Krishnan H. Modification of seed composition to promote health and nutrition. Crop Sci Soc Amer 2009: 263-71.

[7] Taylor DC, Guo Y, Katavic V et al. New Seed Oils for Improved Human and Animal Health: Genetic Manipulation of the Brassicaceae for Oils Enriched in Nervonic Acid. Modification of Seed Composition to Promote Health and Nutrition 2009: 51.

[8] Qi Y, Ji XF, Chi TY et al. Xanthoceraside attenuates amyloid β peptide 1-42 -induced memory impairments by reducing neuroinflammatory responses in mice. Eur J Pharmacol 2017; 820: 18-30.

[9] Ji XF, Chi TY, Liu P et al. The total triterpenoid saponins of *Xanthoceras sorbifolia* improve learning and memory impairments through against oxidative stress and synaptic damage. Phytomedicine 2017; 25: 15-24.

[10] Zhang Y, Xiao LU, Xiao B et al. Research progress and application prospect of *Xanthoceras sorbifolia* for treating Alzheimer's disease. Drug Eval Res 2018; 25: 912-7.

[11] Galbraith DW, Harkins KR, Maddox JM et al. Rapid flow cytometric analysis of the cell cycle in intact plant tissues. Science 1983; 220: 1049-51.

[12] Pellicer J and Leitch I J. The Application of Flow Cytometry for Estimating Genome Size and Ploidy Level in Plants. Methods mol biolo (Clifton, N.J.) 1115:279-307.

[13] Schmutz J, Cannon SB, Schlueter J, et al. Genome sequence of the palaeopolyploid soybean. Nature 2010; 463:178-183.

[14] Tuskan GA, Difazio S, Jansson S, et al. The genome of black cottonwood, *Populus trichocarpa* (Torr. & Gray). Science 2016; 313: 1596-604.

[15] Dolezel J, Greilhuber, J, Suda, J, et al. Estimation of nuclear DNA content in plants using flow cytometry. Nat Protoc 2007; 2: 2233-44.

[16] Toh H, Shirane K, Miura F, et al. Software updates in the Illumina HiSeq platform affect whole-genome bisulfite sequencing. BMC Genomics 2017; 18: 31.

[17] Alberto CM, Sanso AM, Xifreda CC et al. Chromosomal studies in species of Salvia (Lamiaceae) from Argentina. Bot J Linn Soc 2015; 141: 483-90.

[18] Bolger AM, Lohse M, Usadel B et al. Trimmomatic: a flexible trimmer for Illumina sequence data. Bioinformatics 2014; 30: 2114-20.

[19] Martin M. Cutadapt removes adapter sequences from high-throughput sequencing reads. Embnet J 2011; 17: 10-2.

[20] Marçais G, Kingsford C. A fast, lock-free approach for efficient parallel counting of occurrences of k-mers. Bioinformatics 2011; 27: 764-70.

[21] Li X and Waterman M S. Estimating the Repeat Structure and Length of DNA Sequences Using L-Tuples. Genome Res 2003; 13: 1916-22.

[22] Varshney RK, Chen W, Li Y et al. Draft genome sequence of pigeonpea (*Cajanus cajan*), an orphan legume crop of resource-poor farmers. Nat Biotechnol 2012; 30: 83-89.

[23] Li R, Fan W, Tian G et al. The sequence and de novo assembly of the giant panda genome. Nature 2010; 463: 311-29.

[24] Roach M J, Schmidt S and Borneman A R. Purge Haplotigs: allelic contig reassignment for third-gen diploid genome assemblies. BMC Bioinformatics 2018; 19:460-75.

[25] Li MX. karyotyoe analysis of some oil plants. Acta Botanica Boreali-Occidentalia Sinica 1987; 7: 246-51.

[26] Burton J N, Adey A, Patwardhan RP et al. Chromosome-scale scaffolding of de novo genome assemblies based on chromatin interactions. Nat Biotechnol 2013; 31: 1119-25.

[27] Li H and Durbin R. Fast and accurate short read alignment with BurrowsZ Wheeler transform. Bioinformatics, 2009; 25: 1754-60.

[28] Servant N, Varoquaux N, Lajoie BR et al. HiC-Pro: an optimized and flexible pipeline for Hi-C data processing. Genome Biol 2015; 16: 259-70.

[29] Salmela L, Rivals E. LoRDEC: accurate and efficient long read error correction. Bioinformatics 2014; 30: 3506-14.

[30] Fu L, Niu B, Zhu Z et al. CD-HIT: accelerated for clustering the next-generation sequencing data. Bioinformatics 2012; 28: 3150-2.

[31] Parra G, Bradnam K, Korf I et al. CEGMA: a pipeline to accurately annotate core genes in eukaryotic genomes. Bioinformatics 2007; 23: 1061-7.

[32] Simao FA, Waterhouse RM, Ioannidis P et al. BUSCO: assessing genome assembly and annotation completeness with single-copy orthologs. Bioinformatics 2015; 31: 3210-2.

[33] Nishimura O, Hara Y, Kuraku S. Volante for standardizing completeness assessment of genome and transcriptome assemblies. Bioinformatics 2017; 33: 3635-37.

[34] Price AL, Jones NC, Pevzner PA et al. De novo identification of repeat families in large genomes. Bioinformatics 2005; 21: i351-8.

[35] Xu Z, Wang H. LTR_FINDER: an efficient tool for the prediction of full-length LTR retrotransposons. Nucleic Acids Res 2007; 35: 265-8.

[36] Han Y, Wessler SR. MITE-Hunter: a program for discovering miniature inverted-repeat transposable elements from

genomic sequences. Nucleic Acids Res 2010; 38: e199.

[37] Edgar RC, Myers EW et al. PILER: identification and classification of genomic repeats. Bioinformatics 2005; 21: i152-8.

[38] Wicker T, Sabot F, Hua-Van A et al. A unified classification system for eukaryotic transposbale elments. Nat rev Genet 2007; 8: 973-82.

[39] Hoede C, Arnoux S, Moisset M, et al. PASTEC: An Automatic Transposable Element Classification Tool. Plos one 2014; 9: e91929.

[40] Tarailo-Graovac M, Chen N. Using RepeatMasker to identify repetitive elements in genomic sequences. Curr Protoc Bioinformatics 2004, p.4-10.

[41] Jurka J, Kapitonov VV, Pavlicek A et al. Repbase Update, a database of eukaryotic repetitive elements. Cytogenet Genome Res 2005; 110: 462-7.

[42] Kidwell MG, Lisch D. Transposable elements as sources of variation in animals and plants. Proc Natl Acad Sci U S A. 1997; 94: 7704-11.

[43] Zuccolo A, Sebastian S, Yu Y et al. Assessing the Extent of Substitution Rate Variation of Retrotransposon Long Terminal Repeat Sequences in *Oryza sativa* and *Oryza glaberrima*. Rice, 2010; 3: 242-50.

[44] Edgar RC. MUSCLE: multiple sequence alignment with high accuracy and high throughput. Nucleic acids research 2004, 32: 1792-7.

[45] Kimura M. A simple method for estimating evolutionary rates of base substitutions through comparative studies of nucleotide sequences. J Mol Evol 1980; 16: 111-20.

[46] Lin Y, Min J, Lai R et al. Genome-wide sequencing of longan (*Dimocarpus longan* Lour.) provides insights into molecular basis of its polyphenol-rich characteristics. Gigascience 2017; 6: 1-14.

[47] Wu GA, Prochnik S, Jenkins J et al. Sequencing of diverse mandarin, pummelo and orange genomes reveals complex history of admixture during citrus domestication. Nat Biotechnol 2014; 32: 656-62.

[48] Jaillon O, Aury JM, Noel B et al. The grapevine genome sequence suggests ancestral hexaploidization in major angiosperm phyla. Nature 2007; 449: 463-7.

[49] Burge C, Karlin S et al. Prediction of complete gene structures in human genomic DNA. J mol boil 1997; 268: 78-94.

[50] Stanke M, Waack S et al. Gene prediction with a hidden Markov model and a new intron submodel. Bioinformatics 2003; 19: 215-25.

[51] Majoros WH, Pertea M, Salzberg S L et al. Tigr Scan and Glimmer HMM: two open source ab initio eukaryotic gene-finders. Bioinformatics 2004; 20: 2878-9.

[52] Blanco E, Parra G, Guigó R et al. Using geneid to identify genes. Cur Protoc Bioinformatics 2007; 18: 3-4.

[53] Korf I. Gene finding in novel genomes. BMC Bioinformatics 2004; 5: 59-68.

[54] Jens K, Michael W, Erickson J L et al. Using intron position conservation for homology-based gene prediction. Nucleic Acids Res 2016; 44: e89.

[55] Campbell MA, Haas BJ, Hamilton JP et al. Comprehensive analysis of alternative splicing in rice and comparative analyses with Arabidopsis. BMC Genomics 2006; 7: 327-43.

[56] Tang S, Lomsadze A, Borodovsky M et al. Identification of protein coding regions in RNA transcripts. Nucleic Acids Res 2014; 43: 58-68.

[57] Haas BJ, Papanicolaou A, Yassour M et al. De novo transcript sequence reconstruction from RNA-seq using the Trinity platform for reference generation and analysis. Nat Protoc 2013; 8: 1494-512.

[58] Trapnell C, Pachter L, Salzberg S L et al. TopHat: discovering splice junctions with RNA-Seq. Bioinformatics 2009; 25: 1105-11.

[59] Nawrocki EP, Eddy SR et al. Infernal 1.1: 100-fold faster RNA homology searches. Bioinformatics 2013; 29: 2933-5.

[60] Griffiths-Jones S, Moxon S, Marshall M et al. Rfam: annotating non-coding RNAs in complete genomes. Nucleic

Acids Res 2005; 33: 121-4.

[61] Griffithsjones S, Grocock RJ, Van DS et al. miRBase: microRNA sequences, targets and gene nomenclature. Nucleic Acids Res 2006; 34: 140-4.

[62] Lowe TM, Eddy SR et al. tRNAscan-SE: a program for improved detection of transfer RNA genes in genomic sequence. Nucleic Acids Res 1997; 25: 955-64.

[63] She R, Chu JK, Pei J et al. GenBlastA: enabling BLAST to identify homologous gene sequences. Genome Res 2009; 19:143-9.

[64] Birney E, Clamp M, Durbin R et al. GeneWise and Genomewise. Genome Res 2004; 14: 988-96.

[65] Marchlerbauer A, Lu S, Anderson JB et al. CDD: a Conserved Domain Database for the functional annotation of proteins. Nucleic Acids Res 2011; 39: 225-9.

[66] Tatusov RL, Natale DA, Garkavtsev IV et al. The COG database: new developments in phylogenetic classification of proteins from complete genomes. Nucleic Acids Res 2001; 29: 22-8.

[67] Dimmer EC, Huntley RP, Alamfaruque Y et al. The UniProt-GO Annotation database in 2011. Nucleic Acids Res 2012; 40: 565-70.

[68] Du JL; Yuan ZF; Ma ZW et al. KEGG-PATH: Kyoto encyclopedia of genes and genomes-based pathway analysis using a path analysis model. Mol Biosystems 2014;10: 2141-7.

[69] Boeckmann B, Bairoch A, Apweiler R et al. The Swiss-Prot knowledgebase and its supplement TREMBL in 2003. Nucleic Acids Res 2003; 31: 365-70.

[70] Altschul S, Gish W, Miller W et al. Basic local alignment search tool. J Mol Biol 1990; 215: 403-10.

[71] Tang HB, Bowers JE, Wang XY, et al. Perspective- Synteny and collinearity in plant genomes. Science 2008; 320: 486-8.

[72] Li L, Jr SC, Roos DS et al. OrthoMCL: identification of ortholog groups for eukaryotic genomes. Genome Res 2003; 13: 2178-89.

[73] Wang XW, Wang HZ, Wang J et al. The genome of the mesopolyploid crop species *Brassica rapa*. Nature Genet 2011; 43: 1035-40.

[74] Theologis A, Ecker JR, Palm CJ et al. Sequence and analysis of chromosome 1 of the plant *Arabidopsis thaliana*. Nature 2000; 408: 816-20.

[75] Argout X, Salse J, Aury JM et al. The genome of *Theobroma cacao*. Nat Genet 2013; 43:101-8.

[76] Wang K, Wang Z, Li F et al. The draft genome of a diploid cotton *Gossypium raimondii*. Nat Genet 2012; 44:1098-103.

[77] Plomion C, Aury JM, Amselem J et al. Oak genome reveals facets of long lifespan. Nat Plants 2017; 4: 440-52.

[78] Jaillon O, Aury JM, Noel B et al. The grapevine genome sequence suggests ancestral hexaploidization in major angiosperm phyla. Nature 2007; 449: 463-7.

[79] Huang S, Li R, Zhang Z et al. The genome of the cucumber, *Cucumis sativus* L. Nat Genet 2009; 41: 1275-81.

[80] Velasco R, Zharkikh A, Affourtit J et al. The genome of the domesticated apple (*Malus* × *domestica* Borkh.). Nat Genet 2010; 42: 833-9.

[81] Guindon S, Dufayard JF, Lefort V et al. New algorithms and methods to estimate maximum-likelihood phylogenies: assessing the performance of PhyML 3.0. Syst Biol 2010; 59: 307-21.

[82] Talavera G, Castresana J et al. Improvement of phylogenies after removing divergent and ambiguously aligned blocks from protein sequence alignments. Syst Biol 2007; 56: 564-77.

[83] Battistuzzi FU, Billingross P, Paliwal A et al. Fast and slow implementations of relaxed-clock methods show similar patterns of accuracy in estimating divergence times. Mol Biol Evol 2011; 28: 2439-42.

[84] Whelan S, Goldman N. A general empirical model of protein evolution derived from multiple protein families using a maximum likelihood approach. Mol Biol Evol 2001; 18: 691-9.

[85] Yang Z, Nielsen R, Hasegawa M. Models of amino acid substitution and applications to mitochondrial protein evolution. Mol Biol Evol 1998; 15:1600-11.

Click here to access/download;Table;Table 1.xls

Table1 Overview of assembly and annotation for the yellowhorn genome.

Total length	504,196,643 bp
Length of unclosed gaps	73,800 bp
N50 length (initial contigs)	1,044,891 bp
N50 length (scaffolds)	32,173,403 bp
N90 length (scaffolds)	25,069,408 bp
Number of scaffolds (＞N90 length)	21
Largest scaffold	40,097,451 bp
GC content	36.95%
Number of predicted protein-coding genes	24,672
Number of predicted noncoding RNA genes	1,066
Content of repetitive sequences	68.67%
Length of genome anchored on linkage groups	489,286,946 bp (97.04%)

Table 2. Quantity of the contigs anchored with Hi-C.

Group	Number of anchored contigs	Sequence Length (bp)
Lachesis Group 1	68	40,738,791
Lachesis Group 2	92	40,039,835
Lachesis Group 3	38	37,159,809
Lachesis Group 4	112	35,552,403
Lachesis Group 5	84	35,291,867
Lachesis Group 6	62	35,706,508
Lachesis Group 7	66	33,002,525
Lachesis Group 8	46	32,947,898
Lachesis Group 9	66	30,804,552
Lachesis Group 10	62	30,699,318
Lachesis Group 11	68	29,306,026
Lachesis Group 12	56	29,390,540
Lachesis Group 13	47	29,816,145
Lachesis Group 14	71	25,601,946
Lachesis Group 15	72	23,228,783
Total (Ratio %)	1,010 (35.61)	489,286,946 (97.04)

Revisied Figure 2

Click here to access/download;Figure;Fig.2.tif

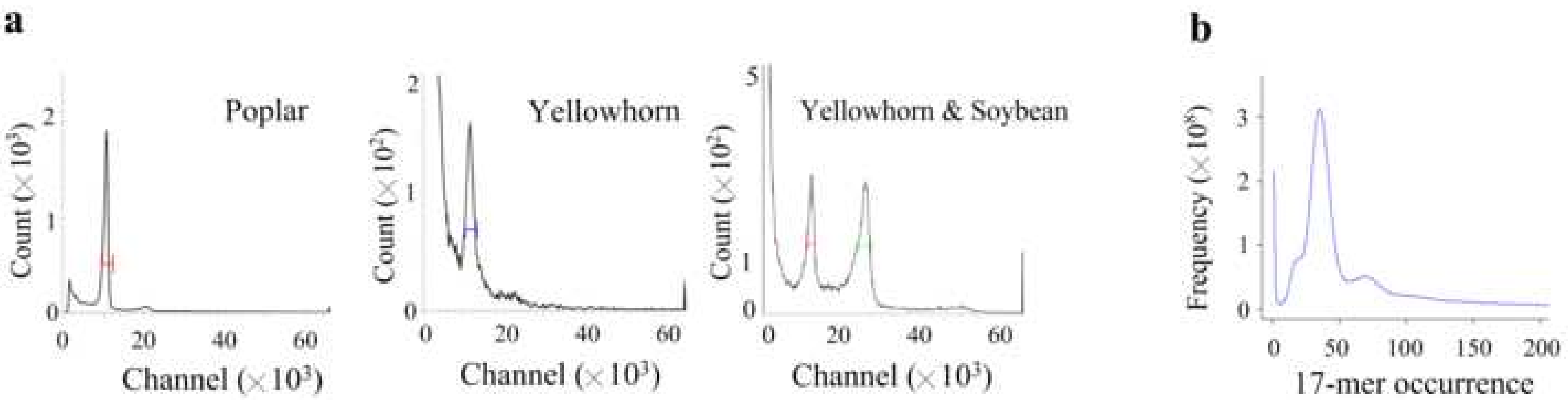

Revisied Figure 3

Click here to access/download;Figure;Fig.3.tif

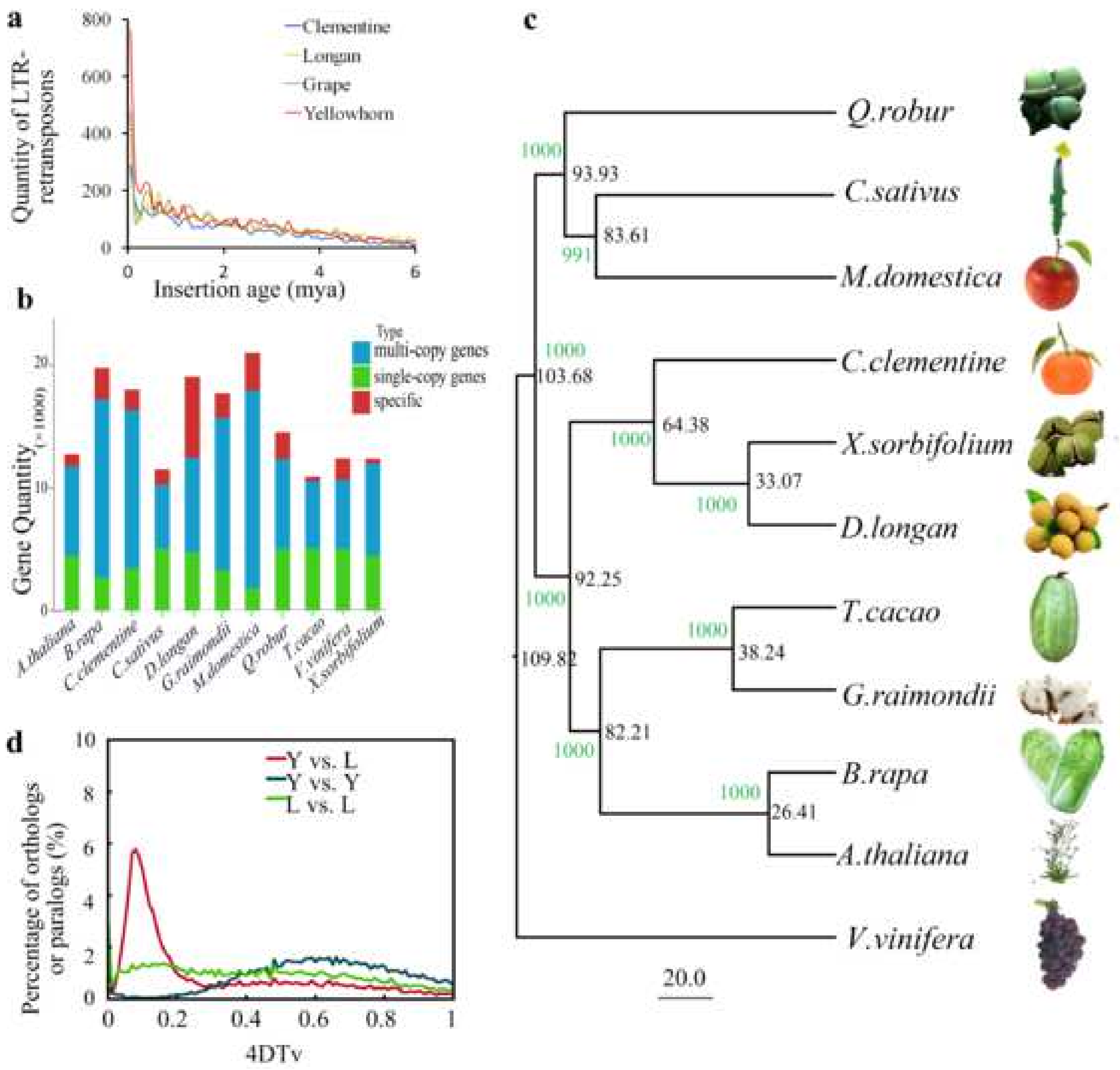

Click here to access/download;Figure;Fig.1.tif

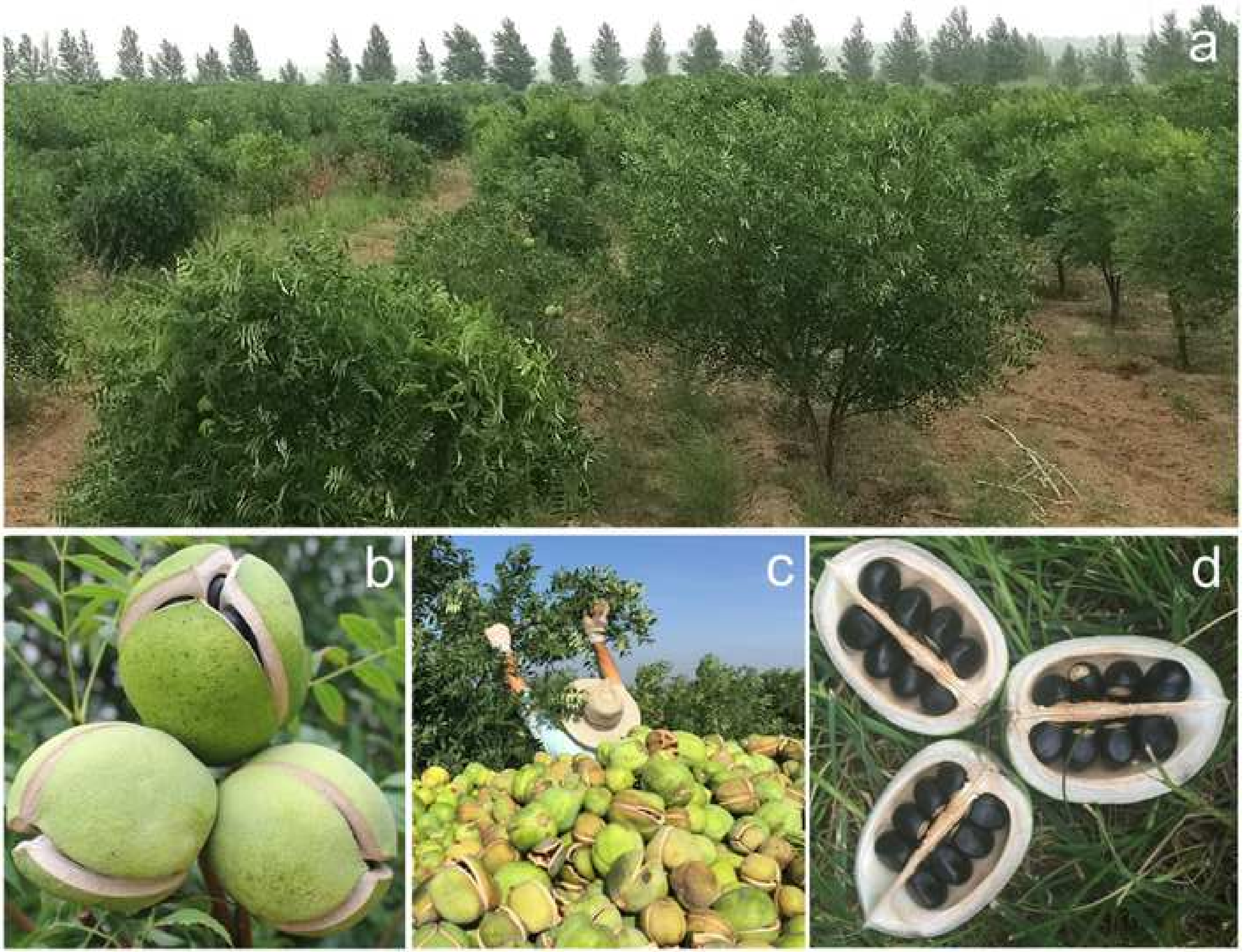

R Figure 4 Click here to access/download;Figure;Fig.4.tif

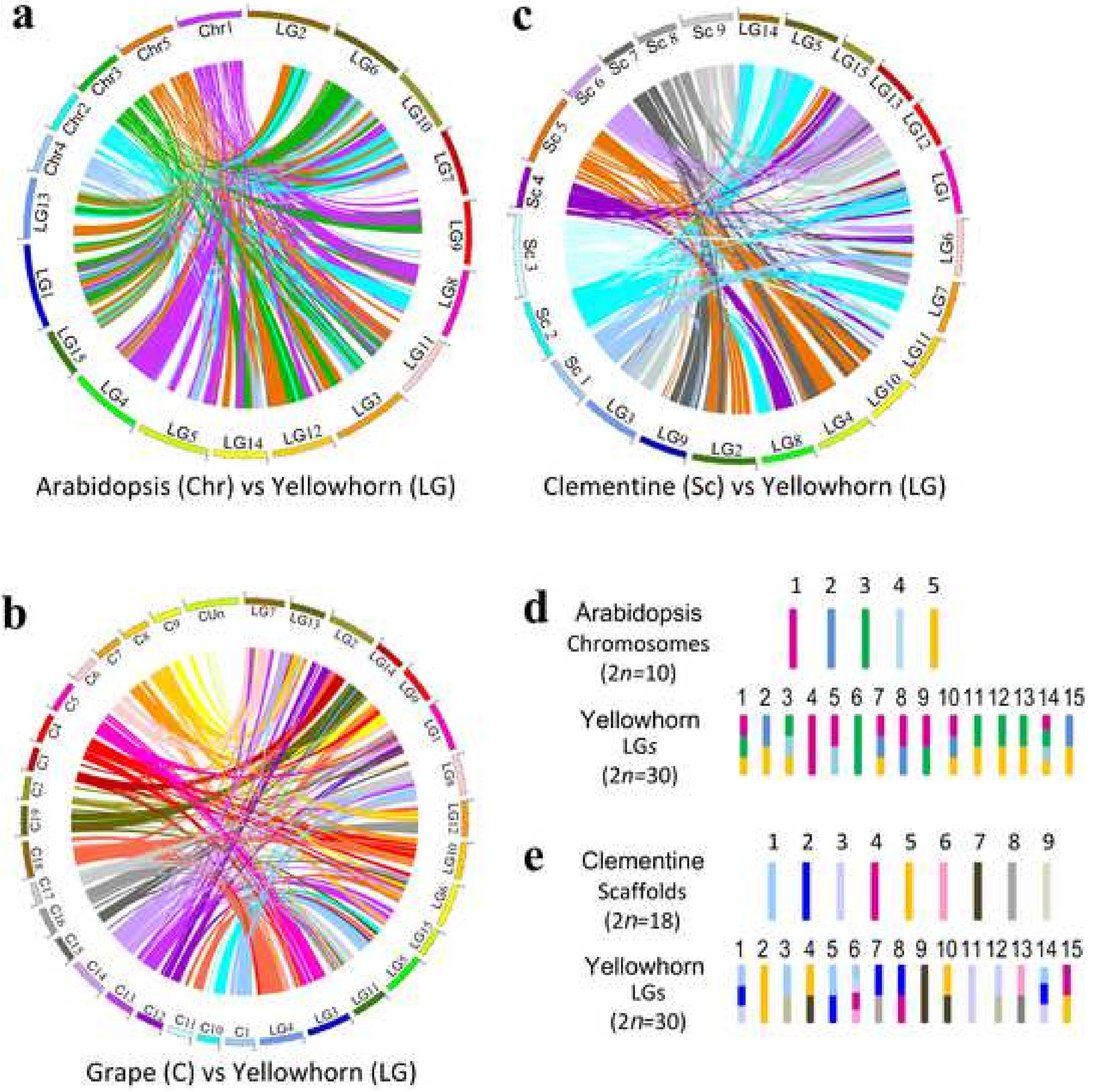

Click here to access/download
Supplementary Material
Figures_AdditionalFiles_2.docx

Click here to access/download
Supplementary Material
Figures_supporting_data.xlsx

Click here to access/download
Supplementary Material
Tables_AdditionalFiles_1.docx

Personal Cover
Click here to access/download;Personal Cover;Response.1.docx

GIGA-D-18-00337

Dear GigaScience editors:

Thank you so much for your thorough review and constructive suggestions. We also thanks three reviewers' professional suggestions and we have responded to the reviewers' comments point-to-point and made corresponding revisions to the manuscript entitled "Pseudomolecules-level assembly of a Chinese oil tree yellowhorn (*Xanthoceras sorbifolium*) genome". The important revisions are outlined as below:

1. In the section of Sequenced individual and sample collection, we described the sequenced individuals more detailedly.
2. In the section of Estimation of genome size through a flow cytometry analysis, we improved the experiment of flow cytometery.
3. In the section of Estimation of the genome size by a *K*-mer analysis, the method was described more detailedly.
4. Calling of heterozygous SNPs was added in the section of SNP calling.
5. In the section of Prediction of protein-coding genes, gene modeling was described more detailedly.
6. In the section of Identification of gene clusters and duplication, Calibration time of fossils is described more detailedly and accurately.
7. We have updated the data of "07.Gene_families_Clusted/" and "10.phylogenetic_tree/" in the website ftp://user15@parrot.genomics.cn.

In addition, we have added two authors Zheng Zhimin and Liu Zhi for their contribution on the data dealing and revise suggestion in the process of manuscript revision.

We think that these revisions have addressed the reviewers' concerns. We look forward to a favorable decision from you.

Thanks and Best wishes!

Yours sincerely

Libing Wang

Response to the reviewers

Reviewer #1:

This manuscript on the genome of Chinese yellowhorn by Bi et al. describes the development and analysis of the assembly of 15 chromosome level pseudo-molecules from a single genotype. The work combines a few different sequencing methods that produced short and long reads and physical map information, along with long RNA-Seq and flow cytometry to independently evaluate the genome size. The combination of methods and bioinformatic steps have apparently produced quite a robust assembly although use of the term "reference genome" may be premature.

The major strength of the paper is the successful integration of the different sequencing methods to produce the assembly that was shown to be complete and of a size that is concordant with the determination by flow cytometry. The evolutionary analysis based on synteny analyses with other angiosperm reference genomes is also well done. The use of PacBio sequencing was advantageous to obtain long RNA transcript sequences. However I found that the determination of the number of genes requires further explanation .

The transcriptome assemply contained over 142k sequences but the authors conclude that there are 24,672 genes in the genome; it needs to be explained how the later was obtained and why there is a difference between the two - is it a technical issue or is there a biological explination?

Response: Thanks for your comments. There were 110,584 non-redundant transcripts in 142,396 transcripts. And among the non-redundant transcripts, 8466 (7.66%) are non-coding mRNAs. Each gene has 2-7 transcripts, of which the largest transcript representing that gene is kept in the final gene model set. This is the reason that 142k transcriptome sequences are corresponding to 24,672 genes. The description is not clear in previous manuscript and we state more clearly in the last paragraph of "Transcriptome sequencing" section in revision (Page 8).

Furthermore, the authors report that there are 172 gene families specific to yellowhorn but do not explain what these genes may encode.

Response: The yellowhorn-specific gene clusters are identified using the OrthoMCL. To identify the

orthologs more correctly, we used diploid *B.rapa* with more single-copy genes (2704), instead of tetraploid *B.napus* (176 single-copy genes). There are 169 gene families specific to yellowhorn. The Annotation information of these gene clusters are listed in the Additional file: Table S7, cited in the Section Identification of gene clusters and duplication. We add related information in section "Identification of gene clusters and duplication" (Page 13).

The manuscript is generally clearly and concisely written but I noted several typos that need correction (especially in the summary) and recommend the manuscript be carefully edited for language. Here are a few significant ones:

Page 2 Line 5: should read "can withstand very cold and drought conditions"

Response: Thanks, it is corrected in the revision in "Backgrounds" section (Page 2).

Page 2 Line 5: I not sure what is meant by "tertiary legacy"

Response: The "Tertiary legacy" was an ancient species that survived from tertiary period. Yellowhorn has mentioned as "tertiary legacy" in Wang (2017). After we checked our data, we found our data was insufficient to reinforcing yellowhorn as a tertiary legacy species. So we delete the discussion of "tertiary legacy" in revision.

Wang Q, Yang L, Ranjitkar S et al. Distribution and in situ conservation of a relic Chinese oil woody species yellowhorn Xanthoceras sorbifolium Bunge. Can J For Res 2017; 47: 1450-6.

Page 2 Line 8: ... understanding...

Response: Sorry for the mistake, it is corrected in "Backgrounds" in Abstract section (Page 2).

Page 2 Line 10: ... genomic era.

Response: Thanks. We correct it in "Backgrounds" in Abstract section (Page 2).

Page 2 Line 13: replace pseudomoleculars with pseudomolecules

Response: Sorry for the mistake. It is corrected in revision.

Page 2 Line 16: The final genome assembly

Response: Thanks, it is corrected in“Findings” section (Page 2).

Page 2 Line 35: The first sentence needs revising. On what basis is it a "reference genome" it is not discussed in the paper as such.

Response: Thanks for your suggestion. We combined different sequence technologies, Illumina, Pacbio and Hi-C, which assembled so far the highest quality genome. The genome coverage is over 94% and the unclosed gap less than 0.15‰ (Table 1). We think that the assemblies are qualified to be used as a reference for study of yellowhorn biology and plant genomics. We added the description of the reference genome in the Section “Pseudomolecules construction and three-dimensional chromatin conformation analysis” of revision (Page 7).

Page 2 Line 38: We did not detect any whole-genome...

Response: The reviewer 2 also found the same error and present recommendations. According to two reviewer’s suggestion, we correct the sentence as "We did not detect evidence of a whole-genome duplication" in “Conclusion” section (Page 2).

Page 2 Line 41: What is meant by "fragment"? Signature?

Response: Fragment here means the syntenic blocks. To avoid confusion, we rewrite the sentence as " The yellowhorn genome carried the syntenic blocks of its ancient chromosomes "in section of “Conclusion” (Page 2).

Page 7 Line 52: replace clustering by clustered

Response: We replace “clustering” by “clustered” in second paragraph of “Transcriptome sequencing” section.

Page 10 Line 52: what is meant by "typical dicot"? Please be more explicit in describing how the species were selected.

Response: Thanks for your suggestion. As far as selection of the typical dicot is concerned, *C. clementina*, *D. Longan* and Yellowhorn are of the Sapindales. *Theobroma cacao* and *Gossypium*

Raimondi, *Quercus robur, Vitis vinifera*, *Cucumis sativus* and *Malus* × *domestica*, *Arabidopsis thaliana* and *Brassica rapa* are the representative species of Malvales, Fagales, Vitales, Cucurbitales, Rosales and Brassicales, respectively. And these species were all belong to Rosids in evolution of species. Besides, most of these species have the high quality genome, which ensures the precision of the gene clustering. The corresponding information is added in Line 3 of first paragraph in section "Identification of gene clusters and duplication".

Page 11 Line 44: What is the meaning of "Paralog curse"?

Response: Sorry for the typo. It should be "Paralog curve". It is corrected in the third to last line in section "Identification of gene clusters and duplication".

Reviewer #2:

This paper reports the whole genome sequence of Xanthoceras sorbifolium (yellowhorn), a tree species whose uses include oil production. Details of the genes and repeats annotated in the X. sorbifolium are reported, as well as the results of some comparative genomic analyses incorporating data from other published plant genome sequences. Hi-C data are used to join X. sorbifolium scaffolds into pseudomolecules, and the final assembly approaches chromosomal level.

However, there is insufficient detail provided for several of the analyses and some other aspects of the manuscript require improvements or clarification, details of which I outline below.

Title

Comment: I recommend rewording the title to remove reference to "conservation of original chromosomes" because the inferences made regarding the conservation of ancestral characteristics within the X. sorbifolium genome are not well supported by the data presented; see further comments on this point below.

Response: Thanks for your suggestion. As we stated in "Chromosome synteny between yellowhorn and reference genomes", we identified a large-scale chromosome synteny bewteen Arabidopsis and yellowhorn. Especially, Arabidopsis Chromosome 1 and yellowhorn Chromosome 4 exhibited the gene collinearity on whole chromosome. However, phylogenetic analysis suggested a distant relationship between Arabidopsis and yellowhorn. So we speculate that they share a chromosome of their origins. Based on this speculation, we named the article: Reference genome of a Chinese yellowhorn *Xanthoceras sorbifolium* provides insights into its conservation of original chromosomes. According to your suggestion, we checked carefully our data and found our data was insufficient to support the previous title. So we changed the title as "Chromosomal-level assembly of a Chinese oil tree yellowhorn (*Xanthoceras sorbifolium*) genome" in revision.

Abstract

Comment: Lines 13/14: Change "pseudomoleculars" to "pseudomolecules"; also needs correcting at

some other places in the text.

Response: Thanks. We correct it in revision and check the manuscript for the typos (Page 2).

Line 16: Change "The final assembly genome" to "The final genome assembly".

Response: Thanks. We correct it in “Findings” section (Page 2).

Lines 38-41: Change "We did not detect the whole-genome duplication" to "We did not detect evidence of a whole-genome duplication".

Response: Thanks. We correct it as "We did not detect evidence of a whole-genome duplication" in “Conclusion” section (Page 2).

Background

Comment: There are various small edits that could be made to improve the clarity of the language used.

Line 32-33: I do not recognise the word "Alzheimerand". I assume this is a typo; should it read "Alzheimer's"?

Response: Thanks. "Alzheimerand" should be "Alzheimer's", we correct it in Background section (Page 3).

Sequenced individuals and sample collection

Please clarify if the DNA used for whole genome sequencing comes a single individual or multiple individuals, as suggested by the title of this section; the subsequent section "Illumina short read sequencing" also indicates that multiple individuals were sequenced because it states that DNA was extracted from leaf tissue from "seedlings".

Response: The DNA used for whole genome sequencing was isolated from a single individual (*X. sorbifolium* cv. Zhongshi 4). To avoid confusion, we correct the sentence as “The fresh young leaves were collected from a single yellowhorn individual” in section “Sequenced individual and sample collection” and “Illumina short-read sequencing” of revision (Page 3).

Also, was a voucher specimen of the sequenced individual(s) made? If so, please provide details of the

specimen (e.g. collector's number) and state the herbarium or other collection in which it is lodged.

Response: Tender leaves were collected from an individual *X. sorbifolium* cv. Zhongshi 4, that is a new variety issued by National Forestry and Grassland Administration (Variety rights No. 20180121) in Zhangwu, Liaoning, China. This tree was produced via clone of a plus tree from natural population in Tongliao, Inner Mongolia, China. We added the description of Sequenced materials in the section" Sequenced individual and sample collection" in revision (Page 3).

Illumina short read sequencing

Page 4, lines 46-49: "leaf tissues of the same soil-grown seedlings of same plants". I am not clear what these samples are supposed to be the same as. Is it the same seedlings as sampled for the PacBio libraries? Also, as mentioned above, clarification is needed regarding how many individuals were used for the whole genome sequencing. If multiple individuals were used, the Authors need to state how many individuals were sampled and whether they were grown from seeds from a single mother tree, or seeds taken from multiple trees of the Zhongshi 4 cultivar.

Response: The tissues sequenced by both Illumina and Pacbio are collected from a single individual (Zhongshi 4). The related information is added in the Section" Illumina short-read sequencing" of revision (Page 4).

Page 5, lines 1-2: Please clarify what is meant by "HCS 2.0.12.0, RTA 1.17.21.3" in relation to "the standard Illumina pipeline".

Response: Image analysis and base calling were performed with HCS 2.0.12.0/RTA 1.17.21.3 to get fastq file raw reads when performed primary data analysis using the standard Illumina pipeline (Toh et al.2017). To avoid confusion, we delete the redundant description in the section "Illumina short read sequencing" of revision (Page 4).

Hidehiro T, Kenjiro S, Fumihito M et al., Software updates in the Illumina HiSeq platform affect whole-genome bisulfite sequencing. BMC Genomics (2017) 18:31-39.

Estimation of the genome size by a K-mer analysis

Page 5, line 16: The Authors state that "level of heterozygosity" was estimated. However, as it is not

entirely clear whether the genome sequence data represents a single or multiple individuals, I'm not sure if heterozygosity is being estimated, or whether it is in fact polymorphism.

Response: As above mentioned, sequenced materials come from a single individual. *K*-mer analysis should estimate a heterozygous rate in this study, not polymorphism.

Page 5, line 21-22: Please specify the details of parameter settings used for Canu; if the default settings were used this should be stated. Also, other than the k-mer size, were any other parameters settings modified for Jellyfish?

Response: In this study, only Pacbio and Hi-C reads were used for genome assembly, the Illumina reads were mapped to genome to correct the sequence error. All the generated PacBio reads were filtered and assembled with and Falcon. We did not use Canu in our study and made mistakes. The Jellyfish parameters were -m 17 -t 10 –s 550M. We add the parameters in section of "Estimation of the genome size by a K-mer analysis" (Page 5).

The reported "heterozygosity" level of 0.36% does not make sense to me given the fact that there is a very prominent peak for heterozygous positions in the k-mer plot in Fig. 2a. In my experience, the type of k-mer frequency profile shown in Fig. 2a suggests a level of heterozygosity far in excess of the value reported; moreover, the fact that it is later reported that the initial genome assembly was significantly larger than the estimated genome size also suggests the actual level of allelic variation within the sequencing data is higher than 0.36%. I have found that with high levels of heterozygosity (e.g. >5%) some k-mer analysis software may fail to properly detect the peaks for heterozygous and homozygous positions. From Fig. 2a, the "hetero" peak seems to be at c. 32x and the "homo" peak at c. 64x. There is also a small shoulder at c. 15x; if this had been erroneously detected as the hetero peak and the c. 32x peak as the homo peak then it would lead to a severe underestimation of the level of heterozygosity in the sequence data, which could explain the mismatch between the value reported and what can be seen in Fig. 2a. The Authors need to provide more details of exactly how the k-mers were used to calculate % heterozygosity, genome size (GS), etc., and also confirm that the hetero and homo peaks were correctly identified during the analysis.

Response: Thanks for your comments. In the previous work, a genome survey was performed and estimated the heterozygosity rates of yellowhorn, which makes mistakes. In maintext, the raw reads

were aligned to the genome assembly using Bowtie 2.2.5 to calculate the heterozygosity rates of genome. The *K*-mer analysis were used to estimate the genome size of yellowhorn. The previous description about how to calculate the heterozygosity rates is not very clear and now it is clearer in revision. To avoid confusion, we delete the preceding previous statements in maintext and rewrite the relevant parts of genome size and heterozygosity rates in the section "Estimation of the genome size by a *K*-mer analysis" and "SNP calling" in revision (Page 5 and Page 7). Meanwhile, we estimate GS via flow cytometry, the result is consistent with *K*-mer analysis. These results indicated the validity of identify of homozygous position.

Estimation of genome size through a flow cytometry analysis

I think it would made sense to move the this section so that it is before the sections on sequencing, seeing as depth of genome coverage is reported in the sequencing sections and at that point the genome size of X. sorbifolium had not been given .

Response: Thanks for your suggestion. The section of flow cytometry analysis moves to the front of the section of sequencing in the revision (Page 4).

It is good to see that the Authors have attempted to estimate GS via flow cytometry (FC) rather than just relying on the estimate from the k-mer analysis. However, there are some issues with the FC analysis. The choice of Populus trichocarpa as a standard for flow cytometry is an unusual one, as this species is not among those that are routinely used for GS estimation by FC in plants (e.g. see Table 1 in Pellicer and Leitch, 2014; DOI 10.1007/978-1-62703-767-9_14). Could the Authors explain why they chose to use P. trichocarpa? Also, no details of the source of the P. trichocarpa material are given (i.e. ex situ collection or original provenance), nor do the Authors specify whether they used the same genotype (Nisqually 1) as used for estimating the reference value for this species . If a different genotype was used, then its GS might differ to that of Nisqually 1, which would in turn create error in the GS estimate for X. sorbifolium. Moreover, the approach used by the Authors does not follow best practice for GS estimation by FC, because the standard and test samples were run separately. Because the exact position of the 2C peak on the flow histogram for a given sample can differ between runs, in order to obtain an accurate estimate of GS, it is important that the standard and test sample are analysed

simultaneously so that the relative position of the peaks can be measured. Can the Authors explain why the P. trichocarpa and X. sorbifolium samples were run separately on the flow cytometer? If the reason was that the peaks were too close together to be easily distinguished when run simultaneously (due to the relatively similar GS of the two species) then the Authors should select an alternative standard (see Pellicer and Leitch, 2014 for details of recommended protocols). Furthermore, please clarify if the 16 samples from P. trichocarpa were from a single individual, or each from a separate individual, and whether each sample was only run once on the flow cytometer, or multiple times. It would also be useful if other specific details, such as the fluorochrome and isolation buffer used, were provided.

Response: Thanks for your comments. The genome size of *P. trichocarpa* is known (Tuskan et,al. 2006 science). And the genome size of *P. trichocarpa* is close to yellowhorn. Also, it is used as standard in terms of stablity of genome size and availability of plant material. Besides, *P. trichocarpa* and yellowhorn both are woody dicotyledons and poplar has been developed as a model in forestry genetic engineering. The *P. trichocarpa* individual, Nisqually 1 genotype, were used as standard reference in this study. In 2018, the tender materials were collected from individual in Chinese academic of forestry. Each sample run three times on the flow cytometer. As you say, when run simultaneously, the peaks were too close together to be easily distinguished due to the relatively similar genome size of yellowhorn and *P. trichocarpa* (Response Fig. 1).

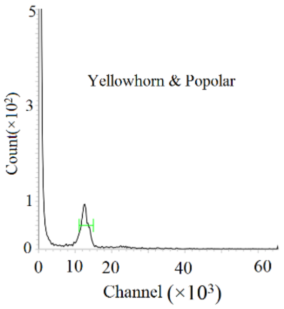

Response Fig.1. Test results of the yellowhorn and internal standards samples using flow cytometry.

According to your comments, we enrich the flow cytometry analysis using the *Glycine max* Var. William 82 (2C genome size=2.28 pg) as reference standards. Three preparations were made: *Arabidopsis* thaliana (2C genome size=0.25 pg), *Glycine max* Var. William 82 (2C genome size=2.28 pg) (Schmutz et al., 2010) and Zea mays L. "CE-777"(2C genome size=5.42 pg) (Pellicer and Leitch 2014). The leaf tissue co-chopped with an internal standard using a razor blade and stained nuclei with

propidium iodide. Each preparation was measured six times, with the relative fluorescence of over 5,000 particles per replicate recorded on a FACSAria flow cytometer (Becton, Dickinson and Company) fitted with a 100-mW green solid state laser (Cobolt Samba; Cobolt, Sweden). The software BDFACSDiva (version 8.0.1) was used for data analysis with the coefficient variation controlled in 5%. The measurement with the soybean internal standard was used as the best estimate of genome size, because the soybean genome size is closest in three internal standards to that of yellowhorn, yielding a more accurate result. The mean peak value of the fluorescence intensity of 16 yellowhorn samples is at round 11,968 while that of soybean is at around 25, 413, the yellowhorn genome size was estimated to be approximately 525.94 Mb (Fig.2b). We add related description in section of "Estimation of genome size through a flow cytometry analysis".

The Arabidopsis Genome Initiative. Analysis of the genome sequence of the flowering plant *Arabidopsis thaliana*. Nature (2000) 408: 796–815.

Jeremy Schmutz, Steven B. Cannon, Jessica Schlueter, Jianxin Ma, Therese Mitros, William Nelson et al. Genome sequence of the palaeopolyploid soybean. Nature(2010) 463:178-183.

Jaume Pellicer and Ilia J. Leitch. The Application of Flow Cytometry for Estimating Genome Size and Ploidy Level in Plants. Methods in molecular biology (Clifton, N.J.) 1115:279-307.

Genome assembly

Please provide details of specific parameter settings used for each piece of software mentioned in this section. In particular, please provide more details of how heterozygous sequences were identified and removed. For example, what criteria were used when deciding which haplotypes to discard and which to keep?

Response: We present more details of software mentioned in this section. The parameters of Falon v0.7:

falcon_sense_option = --output_multi --min_idt 0.70 --min_cov 4 --max_n_read 300 --n_core 8 overlap_filtering_setting = --max_diff 100 --max_cov 100 --min_cov 2 --n_core 12 --bestn 10.

And the heterozygous sequences were identified and removed basing purge_haplotigs pepline, with parameters -a 75 (https://bitbucket.org/mroachawri/purge_haplotigs) (Roach et al. 2018).

In purge_haplotigs pepline, the read-depth analysis is initially performed based on BEDtools (Quinlan et al. 2010) and read-depth histogram is produced for the assembly. We choose three cutoff (depth5, 35, 85) to capture the duplicated regions and properly haplotype-fused regions. Contigs with a high

proportion of bases within the “duplicated” range for read-depth are flagged for possible heterozygous contigs. All flagged contigs were analysised by Sequence alignment to identify synteny with its allelic companion contig. Purge Haplotigs calculates alignment score (the total portion of the flagged contig that aligns at least once) to determine if each flagged contig should be reassigned as a haplotig (-a 75, sequence similarity >75%) and remove the shorter sequence, which is heterozygous sequence.

Quinlan AR, Hall IM. 2010. BEDTools: a flexible suite of utilities for comparing genomic features. Bioinformatics, 26: 841-2.

Roach M J, Schmidt S and Borneman A R. 2018. Purge Haplotigs: allelic contig reassignment for third-gen diploid genome assemblies. BMC Bioinformatics, 19:460-75.

Also, no mention is made of exclusion of organellar sequences; was this performed during the assembly steps, or were these excluded during the preparation of the sequencing libraries?

Response: The organellar sequences are removed using software purge_haplotigs, which identifies contigs with abnormally low or high coverage read-depth with the assumption that they are artefactual. The organelle contigs have a much higher read-depth than the nuclear genome, which are discarded at the beginning steps of assembly (Roach et al., 2018). The related information is added in the Section ”genome assembly” of revision.

Roach M J, Schmidt S and Borneman A R. 2018. Purge Haplotigs: allelic contig reassignment for third-gen diploid genome assemblies. BMC Bioinformatics, 19:460-75.

Pseudomolecules construction and three-dimensional chromatin conformation analysis

Please give details of any specific parameter settings used for the BWA and HiC-Pro software.

Response: Thanks. We add the parameter setting for BWA and HiC-Pro software in “Pseudomolecules construction and three-dimensional chromatin conformation analysis” section (Page 6).

I don't think the actual chromosome number of *X. sorbifolium* (2n = 30) is mentioned anywhere in the paper; it would make sense to include reference to the chromosome number in this section.

Response: Karyotype of yellowhorn was identified in Lang (1980) and Li (1987). The chromosome

number and karyotype of yellowhorn was 2n=30. The related information was added in the Section" Pseudomolecules construction and three-dimensional chromatin conformation analysis" of revision (Page 7).

Li MX. 1987. karyotype analysis of some oil plants. Acta Botanica Boreali-Occidentalia Sinica 7(4):246-251.

Lang GX, Liu WL, Ma LL et al. 1980. Chromosome numbers and karyotype of yellowhorn (Xanthoceras sorbifolium). Forest science and technology. 10(6):9-10.

Transcriptome sequencing

Please clarify if the tissues used from RNA extraction were from a single or multiple individuals, and whether the same plant(s) was sampled as for the DNA extractions.

Response: Genomic DNA was isolated from a single individual. Total RNAs are isolated from the same individuals as that for the DNA extraction. We describe clearer in section "Transcriptome sequencing".

Page 7, lines 51/52: Please specify any parameter settings used with the CD-HIT software.

Response: We add the paraments setting of CD-HIT (-c 0.99 -T 6 -G 0 -aL 0.90 -AL 100 -aS 0.99 -AS 30.) in second paragraph in section "Transcriptome sequencing" (Page 9).

Evaluation of assemble quality

Correct the title of this section to "Evaluation of assembly quality".

Response: We correct the title of this section as "Evaluation of assembly quality" in the revision.

Page 8, lines 16: "including 83.2% single-copy and 11.5% duplicated genes"; these values don't match those in Table S2, please double-check.

Response: Sorry for the mistake. We correct the two numbers "including 89.0% single-copy and 5.7% duplicated genes" in this section, where the errors occurred when citing the numbers form Table S2.

Annotation of the repetitive sequences

Please provide details of specific parameter settings used for each piece of software mentioned in this section; currently they are only given for RepeatMasker.

Response: Thanks. We add the parameters of software we used in section of Annotation of the repetitive sequences (Page 9).

Page 8, line 40/41: Change "of the yellowhorn genome in length" to of the yellowhorn genome assembly".

Response: Thanks. We rewrite the sentence as "of the yellowhorn genome assembly" in first line of second paragraph in "Annotation of the repetitive sequences" section.

Page 8, line 43/44: Please clarify why the results from X. sorbifolium are compared with Citrus sinensis in particular; is this the next most closely related species with a whole genome assembly available after longan? Also, if all the percentages of repeats quoted are expressed in terms of percentage of the assembly size then the comparison may not be very informative if the assemblies for the other species are less complete.

Response: Thanks for your comments. Phylogenetic analysis suggested that a close relationship between Longan and yellowhorn, and *Citrus sinensis* is the next most closely related species with a whole genome assembly available after longan, all belonging Sapindales. So we compared the percentages of repeats of these species. We combined more sequence technologies (Illumina, Pacbio and Hi-C) while longan and *C.sinenis* were pure NGS (Next Generation Sequencing), which assembled higher quality genome than other two species. As you say, the comparison may not be very informative when the assemblies for the other species are less complete. So we deleted the discussion of the comparison of repeats percentages.

Page 8, line 49: Please double-check all of the percentage values in Table S3, as some appear to be slightly wrong.

Response: Sorry for the mistake. We double-checked the percentage values in Table S3 and corrected the errors (Table S3).

Page 8, line 57/58: Please provide details of any software used for the calculation of LTR insertion times; if custom scripts were used, these should be provided.

Response: LTR_FINDER（Version : 1.07,with default setting parameters）was used to scanning the LTR sequences. Insertion times for the LTR-retrotransposons were estimated using the DNA divergence between the pair of LTR sequences (SanMiguel et al., 1998).To calculate the insertion age of each LTR retrotransposons, 5' and 3' LTRs of the same element were aligned with MUSCLE (version 3.8.31) (with default setting parameters) (Edgar 2004). Distmat (with default parameters) was used to estimate the DNA divergence between the LTR sequences with the Kimura-2-parameter base substitution Model (Kimura, 1980). We add related information in "Annotation of the repetitive sequences" section (Page 9, 10).

SanMiguel P, Gaut BS, Tikhonov A et al. 1998. The paleontology of intergene retrotransposons of maize. Nat Genet. 20:43–45.

Kimura M. A simple method for estimating evolutionary rates of base substitutions through comparative studies of nucleotide sequences. J Mol Evol. 1980 Dec;16(2):111-20.

Edgar RC: MUSCLE: multiple sequence alignment with high accuracy and high throughput. Nucleic acids research 2004, 32:1792-1797

Page 8, line 60: Please provide details of the source of data used for clementine, longan and grape (either here or in a Table); please specify which versions of genome assemblies and annotations were used.

Response: We add the related information in section of "Annotation of the repetitive sequences" (Page 10).

Page 9, lines 2-5: The Authors make some very broad suggestions about why X. sorbifolium may have a larger number of young LTR insertions compared with the other species, but later they indicate that the differences between species could largely be due to differences in assembly quality, which makes the preceding text somewhat redundant.

Response: Thanks for your comments. We observed that the yellowhorn genome carried more young LTR-retrotransposons, so we speculate that the transposon in yellowhorn genome frequently exchanged horizontally or vertically, self-replication and self-splicing to adapt to environmental changes. In addition, the yellowhorn genome may have higher quality assembly, which also have led to an slight

under-estimation of the LTR-retrotransposons in clementine and longan. To avoid confusion, we delete the discussion of “the differences between species could largely be due to differences in assembly quality” in revision.

Page 9, line 18/19: Correct "LTR-retrotranpsons" to "LTR-retrotransposons".

Response: We correct the typo in revision.

Page 9, line 21/22: Change "which led to an under-estimated quantity of the" to "which may have led to an under-estimation of the".

Response: We rewrite the sentence as "which may have led to an under-estimation of the" in last line in "Annotation of the repetitive sequences" section.

Also, Table S3 lists "PotentialHostGene" among the types of repeats, but these are not mentioned in the text. This category of "repeats" makes up c. 5% of the genome assembly; could these be protein-coding genes that have been masked erroneously? Was any pre-masking of repeat libraries done for captured gene fragments present within repeats, that might cause host genes to be masked as repeats by mistake? Also, were high-copy number genes, such as rDNA genes, accounted for? Or will these also have been masked as repeats? These points need clarifying; if protein-coding genes have been masked as repeats by mistake this would lead to an inflated estimate of the proportion of repetitive DNA and an underestimation of the number of genes within the X. sorbifolium genome.

Response: The Potential Repeat Host Genes, as well as the rDNA, SSRs, and other repetitive elements can be automatically classified by PASTEC (Hoede et al. 2013). The Potential Repeat Host Genes are not the real host genes but the repeat host genes. As mentioned by Hoede in 2013, they should be grouped into the repetitive items. We used standard repeat prediction pipeline (RepeatModeler + RepeatMasker) to predict the repetitive sequences. When we used the repeat-masked genome to perform the EVM gene modeling, highest weights were assigned to evidence-based prediction, such as RNA-Seq data and homologs. If a high-copy gene was masked as a potential repeat host gene, it can be called back by the gene prediction pipelines when it has the support of transcriptome data or homologs. The high-copy protein-coding genes do not include any non-coding mRNA genes, such as the rDNA genes.

Hoede C, Arnoux S, Moisset M et al. PASTEC: An Automatic Transposable Element Classification Tool. Plos one 2014, 9(5):e91929

Prediction of RNA genes

Where not given, please provide details of specific parameter settings used for each piece of software mentioned in this section. In particular, please give more details of the filtering thresholds used with EVidenceModeler.

Response: We add related information in first paragraph of "Prediction of protein-coding RNA genes" section. When conducting the EVM integration (Mode:STANDARD S-ratio: 1.13 score>1000) , weights assignment was as follows:

PROTEIN	OTHER	50
PROTEIN	GeMoMa	50
TRANSCRIPT	assembler-PASA	50
TRANSCRIPT	Stringtie	20
ABINITIO_PREDICTION	genscan	0.3
ABINITIO_PREDICTION	AUGUSTUS	0.3
ABINITIO_PREDICTION	GlimmerHMM	0.3
ABINITIO_PREDICTION	SNAP	0.3
ABINITIO_PREDICTION	geneID	0.3
ABINITIO_PREDICTION	GeMoMa	0.3
OTHER_PREDICTION	OTHER	100

Page 9, line 40/41: Please specify which version of the A. thaliana annotation was used.

Response: We add the *A. thaliana* annotation version (TAIR 10) in first paragraph of "Prediction of protein-coding RNA genes" section.

Also "homology-based prediction" should really read "similarity-based prediction ".

Response: We correct it in first paragraph of "Prediction of protein-coding RNA genes" section (Page 10).

Page 9, lines 43/47: Please reword "were used as the reference databases aligned the homolog genes in the yellowhorn genome". As currently written, I am not sure what this is supposed to mean.

Response: Thanks. We rewrite the sentence as" During the EVM integration, higher weights were assigned to the predicted PASA and GeMoMa models than the ab initio models. The PASA was used to modify the final gene model." (Page 11).

Page 9, line 57/59: Sentence starting "Finally, the ab initio predicted transcripts", please explain more clearly what is actually been done in this final step.

Response: After conducting the EVM integration, the PASA was used to modify the final gene model. To avoid confusion, we reword this sentence more clearly in section of "Prediction of protein-coding RNA genes" (Page 11).

Also, please clarify if any filtering of the GeMoMa gene predictions was done prior to this point.

Response: When the multiple transcripts predicted at the same location, the best GeMoMa scoring transcript was chosen as the optimal model. We add related description in section of "Prediction of protein-coding RNA genes" (Page 10, 11).

Page 10, line 13: Change "was used to pseudogene prediction" to "was used to perform pseudogene prediction".

Response: Thanks, we corrected it in the first line of third paragraph in "Prediction of protein-coding RNA genes" section (Page 11).

Page 10, line 21/22: Change "The genes were annotated" to "The genes were annotated functionally".

Response: Thanks, we corrected it in the first line of fourth paragraph in "Prediction of protein-coding RNA genes" section (Page 11).

Page 10, line 26/27: Please specify which version of BLAST2GO was used.

Response: Version 2.2.31 of BLAST2GO was specified in fourth paragraph of "Prediction of protein-coding RNA genes" section (Page 11).

Page 10, line 38: 24,429 is not 98.97% of 24,672; please double-check these values.

Response: Sorry for the mistake. After checking the values, we annotate 24,429 carried at least one

functional domain with the alignments to the protein database, which is 99.02% of 24672 protein-coding genes. We correct the mistake in last paragraph “Prediction of protein-coding RNA genes” section (Page 11).

Identification of gene clusters and duplication

Page 10, line 51/52: Please specify any parameters settings used for OrthoMCL and state whether any filtering of the input sequences was performed: e.g., to remove multiple splice variants, organellar sequences or very short sequences.

Response: Thanks for your comments. We add the parameters of OrthoMCL in revision. We select the longest transcripts represents a gene to perform OrthoMCL analysis (Page 13).

Also, change "dicot" to "eudicot"; this needs correcting in several other places as well.

Response: Sorry for the mistakes. We check the whole manuscript and correct them in revision (Page 13).

Page 10: For the ten species included in the OrthoMCL analysis, as well as citing the original publications for the genome sequencing, please specify the versions of the genome assemblies and annotations used for each taxon and state where the data were obtained from (e.g. TAIR, Phytozome, etc.).

Response: We add related information in Additional file Table S6 and in section “Identification of gene clusters and duplication” of revision (Page 13).

Page 10, line 54/55: Change "Cruciferous" to "Brassicaceae".

Response: We correct it in first paragraph of “Identification of gene clusters and duplication” section (Page 13).

Page 11, line 7/8: Change "species-special" to "species-specific".

Response: We correct it in first paragraph of “Identification of gene clusters and duplication” section (Page 13).

Page 11, lines 10-16: It cannot be concluded that X. sorbifolium genes "might keep more structural characters of their ancestors" simply because this species appears to have relatively few genes that are specific to itself alone.

Response: Thanks for your comments. Evidence of gene clusters might be not sufficient to draw this conclusion but to be an indicative gene conservation between yellowhorn with their ancestors. To make a clear description, we rewrite the sentence as “The yellowhorn genes might conserve the similar gene structure with their origins.” at the end of the first paragraph in “Identification of gene clusters and duplication” section.

Page 11, lines 21-22: Please provide further details of how the phylogenetic analysis was done with PHYML ; e.g., were DNA or protein sequences analysed? Which model of sequence evolution was used? How was support assessed? Etc. Also, the tree in Fig. 3c is rooted on Cucumis sativus, whereas the appropriate outgroup for the set of taxa included in the analysis would be Vitis vinifera. However, even if the tree was rerooted on v. vinifera, the topology is incongruent with the results obtained by previous studies (see for example the summary in Figure 1 of THE ANGIOSPERM PHYLOGENY GROUP 2016, Botanical Journal of the Linnean Society, 181: 1-20). What is the explanation for this? One possibility is that not all gene families (i.e. OrthoMCL clusters) analysed are comprised of solely orthologous sequences; just because the gene clusters/families are single copy doesn't mean all of their members are orthologous, and inclusion of paralogous sequences could confound phylogenetic inference of species relationships. Also, support values (e.g. bootstrap percentages) need to be added to Fig. 3c.

Response: We described the methods and parameters more detailedly in the revision.

The protein sequences of common single copy genes were used to phylogeneitc analysis. Then the model of TIM2+I+G was used to construction the evolution tree. The model was selected by the jmodeltest output. And the jmodeltest output was as follows:

Best Models:

Model f(a) f(c) f(g) f(t) kappa titv Ra Rb Rc Rd Re Rf pInv gamma

----

BIC TIM2+I+G 0.25 0.23 0.25 0.27 0.00 0.00 1.504 3.664 1.504 1.000 4.373 1.000 0.32

1.44

We add the bootstarp in Fig. 3c.

According the reviewers' comment, we rebuild the phylogenetic tree using grape as the outgroup. We also make improvement in constructing the tree. To identify the orthologs more correctly, we used diploid *B.rapa* instead of tetraploid *B.napus*. Thus, the topology of *A.thaliana*, *B.rapa*, *T.cacao*, *G.raimondii*, *X.sorbifolium*, *D.longan*, *C.clementina* is consistent with the previous report (The Angiosperm Phylogeny Group 2016, Botanical Journal of the Linnean Society, 181: 1-20). As far as the slightly different topology of *Malus_x_domestica*, *C.sativus* and *Q.robur* is concerned, it might be owing to our selection of more nuclear genes while that reported tree was constructed by only rbcL, atpB and 18S rDNA genes.

An ordinal classification for the families of flowering plants. The Angiosperm Phylogeny Group. Annals of the Missouri Botanical Garden 1998; 85(4):531–553.

Page 11, line 24: Change "the orthologs" to "the putative orthologs".

Response: We change "the orthologs" to "the putative orthologs" in "Identification of gene clusters and duplication" section (Page 15).

Page 11, line 27: Details of how the divergence time estimation was carried out with MCMCtree are lacking. The Authors need to report the parameter settings used, including which molecular clock model was used, and provide details of any fossils used for calibrating the tree. Also, there are no credibility intervals reported for the divergence time estimates in Fig. 3c and the main text; these need to be added.

Response: Thanks your suggestion. Common single-copy gene families were identified and chosen to estimate the divergence time using MCMCtree. The setting parameters of MCMCtree is as follows: burn-in=10,000, sample-number=100,000, sample-frequency=2.

The TimeTree database (http://www.timetree.org/), r8s (parameter: r8s -b -f r8s_in.txt > r8s_out.txt) and divergence time mentioned by Whelan (2001) and Yang (1998) were used for calibrating the time. Calibration time of fossils used in evolutionary trees is as follows:

(((Qrob,(Csat,Mdom)),((Ccle,(Xsor,Dlon)),((Tcac,Grai),(Brapa,Atha)'<30.9>20.4'))),Vvin)'<115>105' .

The credibility intervals for the divergence time estimates was as follows:

UTREE 1 = (((Qrob: 93.929608, (Csat: 83.608799, Mdom: 83.608799) [&95%={67.268, 96.218}]: 10.320809) [&95%={78.104, 105.034}]: 9.748170, ((Ccle: 64.380901, (Xsor: 33.069679, Dlon: 33.069679) [&95%={18.376, 48.565}]: 31.311222) [&95%={46.354, 81.164}]: 27.870851, ((Tcac: 38.243394, Grai: 38.243394) [&95%={21.870, 56.407}]: 43.965024, (Brapa: 26.409279, Atha: 26.409279) [&95%={20.721, 30.886}]: 55.799139) [&95%={67.279, 94.364}]: 10.043334) [&95%={77.382, 103.299}]: 11.426026) [&95%={89.679, 113.000}]: 6.145826, Vvin: 109.823604) [&95%={104.966, 114.982}].

We add the related information in second paragraph of "Identification of gene clusters and duplication" (Page 15).

Whelan, S. and N. Goldman. 2001. A general empirical model of protein evolution derived from multiple protein families using a maximum likelihood approach. Molecular Biology and Evolution 18, 691-699.

Yang, Z., Nielsen R and Hasegawa M. 1998. Models of amino acid substitution and applications to mitochondrial protein evolution. Mol. Biol. Evol. 15:1600-1611.

Page 11, line 29/30: Sentence starting "As two species" needs rewording to improve clarity.

Response: To improve clarity, the sentence has been changed to "In Sapindaceae Family, yellowhorn and longan are indicative of the closest relationship" in second paragraph of "Identification of gene clusters and duplication" (Page 15).

Page 11, line 43/44: Change both instances of "curse" to "curve".

Response: We change "curse" to "curve" in this section (Page 16).

Chromosome synteny between yellowhorn and reference genomes

It might make more sense to move this section to before the section "Identification of gene clusters and duplication" as the results of the synteny analysis are mentioned in that section.

Response: As you advised, we moved this section to before the section "Identification of gene clusters and duplication" in the revised version.

Please specify any parameter settings used for MCscan.

Response: We add the parameter for MCscan in section of "Chromosome synteny between yellowhorn and reference genomes" (Page 12).

Page 12, line 40/41: Correct "systemic" to "syntenic".

Response: We change "systemic" to "syntenic" in last line in page 12.

Page 12, line 40/41: Correct "collineartiy" to "collinearity".

Response: We correct "collineartiy" to "collinearity" in first paragraph of section “Chromosome synteny between yellowhorn and reference genomes”.

The arguments made in this section relating to evidence for conservation of "ancient" chromosomes and support for the "tertiary legacy" status of X. sorbifolium are not convincing to me and I find the text quite confusing and hard to follow. Further clarification is required if this part of the manuscript is to be retained.

Response: Thanks for your comments. As we stated in “Chromosome synteny between yellowhorn and reference genomes”, we identified a large-scale chromosome synteny bewteen Arabidopsis and yellowhorn. Especially, Arabidopsis Chromosome 1 and yellowhorn Chromosome 4, exhibited the gene collinearity on whole chromosome. However, phylogenetic analysis suggested a distant relationship between Arabidopsis and yellowhorn, implicating they share a chromosome of their origins. That is not an adequate explanation for conservation of "ancient" chromosomes and support for the "tertiary legacy" status. So we deleted the discussion of "tertiary legacy" and "ancient" chromosomes. The related information is added in section“Chromosome synteny between yellowhorn and reference genomes” of revision.

Legends

When referring to "mellow fruit", do the Authors mean "ripe fruit"?

Response: Yes, to clarity clearly, we correct "mellow fruit" to " ripe fruit " in legends.

Table1 & 2

I suggest replacing "quantity" with "number". E.g. "Number of scaffolds" rather than "Quantity of scaffolds".

Response: We replace" number" to "quantity" in Table 1 & 2.

Reviewer #3:

Thank you for the opportunity to review this Data Note for GigaScience. The MS GICA-D-18-00337 entitled "Reference genome of a Chinese yellowhorn Xanthoceras sorbifolium provides insights into its conservation of original chromosomes" reports on a study aiming to present a high quality genome and to determine the evolutionary history of this species. The presence of whole genome duplication is not detected and the genome structure has received a detailed explanation.

That said, I would like to ask if does the accumulation of LTR-retrotransposons enriched with specific protein coding genes? Also, I would like to know (if it is possible, I think so) which category is younger? Copia or Gypsy?

Response: Thanks for your comments. When we compare the loci between LTR-retrotransposons and protein-coding coding genes, we did not find the correlationship between them. Distribution of the insertion ages for *Copia*-type and *Gypsy*-type are plotted in Fig.S5. No significant difference is observed between *Copia*-type and *Gypsy*-type LTR-retrotransposons in the yellowhorn genome.

I think a better description about K-mer analysis must be provided, in addition, to cite the paper Marçais et al. Also some minor changes must be addressed before this MS can be accepted.

Response: Thanks for your comments. We cite the paper Marçais et al (2011) and reword this section in revision (Page 5).

Some minor edits

Page 3 Line 33: change the word Alzheimerand

Response: We revised the typo "Alzheimerand" with "Alzheimer's" in section of Background.

Page 6 Line 24: please check the N50 number: 1.39Mb or 1.04Mb, please clarify it.

Response: Thanks for your suggestion. After genome assembly using Pacbio reads, we generated contig with N50 1.39M. In section of “Pseudomolecules construction and three-dimensional chromatin conformation analysis”, effective Hi-C Reads were aligned to preliminary assembled sequences and

corrected the error of preliminary draft Genome. After corrected, the contig N50 was 1.04M, which was the final N50 number. To avoid confusion, we deleted the confusing N50 number (1.39M) in revised version.

Page 12, Line1: please add a short comment about MCScan's representation, circular shape or circle plot.

Response: Thanks for your suggestion. The gene collinearity was constructed by anchored the aligned yellowhorn genes on the reference genomes, clementine, Arabidopsis and grape, respectively, using the Mutilple Collinearity Scan toolkit (MCscan). We add the description about MCScan's representation, circular shape or circle plot. In Legends Fig.4, the circularized blocks represent the chromosomes of yellowhorn and the other genome. Aligned genes identified by the MCscanX are connected by the lines, of which the located chromosomes are shown in different colors.

Page 12 Line 57, please improve the sentence "as mentioned above analysis of genes …,", something as: "as mentioned before,"

Response: Thanks. We delete related discussion of “yellowhorn as a tertiary legacy species” in revision.
