# Supplementary material for: Pseudomolecule-level assembly of the Chinese oil tree yellowhorn (Xanthoceras sorbifolium) genome
Source: Gigascience. 2019 Jun 26;8(6):giz070. doi: 10.1093/gigascience/giz070 (PMC6593361; doi:10.1093/gigascience/giz070)
Supplement: giz070_GIGA-D-18-00337_Revision_2 [file giz070_giga-d-18-00337_revision_2.pdf]

GigaScience

Pseudomolecule-level assembly of the Chinese oil tree yellowhorn (Xanthoceras sorbifolium) genome

--Manuscript Draft--

**Manuscript Number:**	GIGA-D-18-00337R2
**Full Title:**	Pseudomolecule-level assembly of the Chinese oil tree yellowhorn (Xanthoceras sorbifolium) genome
**Article Type:**	Data Note
**Funding Information:**	the Central Public-Interest Scientific Institution Basal Research Fund (CAFYBB2017QB001, CAFYBB2019QD001) — Dr. Libing Wang<br>the National "12th Five-Year" Plan for Science & Technology Support of China (2015BAD07B0106) — Dr. Chengjiang Ruan<br>Major Research Plan (31800571, 31870594, 31760213) — Dr. Libing Wang<br>National Key Research and Development Plan of China (2016YFC050080506) — Dr. Libing Wang
**Abstract:**	Background: Yellowhorn (Xanthoceras sorbifolium) (NCBI Taxonomy ID: 99658) is a species of the Sapindaceae family native to China and is an oil tree that can withstand cold and drought conditions. A pseudomolecule-level genome assembly for this species will not only contribute to understanding the evolution of its genes and chromosomes, but also bring yellowhorn breeding into the genomic era.<br>Findings: Here, we generated 15 pseudomolecules of yellowhorn chromosomes, on which 97.04% of scaffolds were anchored, using the combined Illumina HiSeq, PacBio Sequel and Hi-C technologies. The length of the final yellowhorn genome assembly was 504.2 Mb with a contig N50 size of 1.04 Mb and a scaffold N50 size of 32.17 Mb. Genome annotation revealed that 68.67% of the yellowhorn genome was composed of repetitive elements. Gene modelling predicted 24,672 protein-coding genes. By comparing orthologous genes, the divergence time of yellowhorn and its close sister species longan (Dimocarpus longan) was estimated at approximately 33.07 million years ago. Gene cluster and chromosome synteny analysis demonstrated that the yellowhorn genome shared a conserved genome structure with its ancestor in some chromosomes.<br>Conclusions: This genome assembly represents a high-quality reference genome for yellowhorn. Integrated genome annotations provide a valuable dataset for genetic and molecular research in this species. We did not detect whole-genome duplication in the genome. The yellowhorn genome carries syntenic blocks from ancient chromosomes. These data sources will enable this genome to serve as an initial platform for breeding better yellowhorn cultivars.
**Corresponding Author:**	Libing Wang, Ph.D.<br><br>CHINA
**Corresponding Author Secondary Information:**	
**Corresponding Author's Institution:**	
**Corresponding Author's Secondary Institution:**	
**First Author:**	Quanxin Bi
**First Author Secondary Information:**	
**Order of Authors:**	Quanxin Bi
	Yang Zhao

	Wei Du
	Ying Lu
	Lang Gui
	Zhimin Zheng
	Haiyan Yu
	Yifan Cui
	Zhi liu
	Tianpeng Cui
	Deshi Cui
	Xiaojuan Liu
	Yingchao Li
	Siqi Fan
	Xiaoyu Hu
	Guanghui Fu
	Jian Ding
	Chengjiang Ruan
	Libing Wang, Ph.D.
**Order of Authors Secondary Information:**	
**Response to Reviewers:**	See the personal cover letter in the last section of the GIGA-D-18-00337_R2
**Additional Information:**	
**Question**	**Response**
Are you submitting this manuscript to a special series or article collection?	No
**Experimental design and statistics**<br><br>Full details of the experimental design and statistical methods used should be given in the Methods section, as detailed in our Minimum Standards Reporting Checklist. Information essential to interpreting the data presented should be made available in the figure legends.<br><br>Have you included all the information requested in your manuscript?	Yes
**Resources**<br><br>A description of all resources used, including antibodies, cell lines, animals and software tools, with enough	Yes

Powered by Editorial Manager® and ProduXion Manager® from Aries Systems Corporation

information to allow them to be uniquely identified, should be included in the Methods section. Authors are strongly encouraged to cite Research Resource Identifiers (RRIDs) for antibodies, model organisms and tools, where possible.<br><br>Have you included the information requested as detailed in our Minimum Standards Reporting Checklist?	
**Availability of data and materials**<br><br>All datasets and code on which the conclusions of the paper rely must be either included in your submission or deposited in publicly available repositories (where available and ethically appropriate), referencing such data using a unique identifier in the references and in the "Availability of Data and Materials" section of your manuscript.<br><br>Have you have met the above requirement as detailed in our Minimum Standards Reporting Checklist?	Yes

Powered by Editorial Manager® and ProduXion Manager® from Aries Systems Corporation

Manuscript Click here to access/download;Manuscript;YellowhornBi.docx

Click here to view linked References

Pseudomolecule-level assembly of the Chinese oil tree yellowhorn (*Xanthoceras sorbifolium*) genome

Quanxin Bi[1,2,†], Yang Zhao[1,†], Wei Du[2,†], Ying Lu[3], Lang Gui[3], Zhimin Zheng[4,5], Haiyan Yu[1,6], Yifan Cui[1], Zhi Liu[4,5],Tianpeng Cui[7], Deshi Cui[7], Xiaojuan Liu[1], Yingchao Li[1], Siqi Fan[1], Xiaoyu Hu[1], Guanghui Fu[1], Jian Ding[2], Chengjiang Ruan[2,*], Libing Wang[1,*]

[1] State Key Laboratory of Tree Genetics and Breeding, Research Institute of Forestry, Chinese Academy of Forestry, Beijing 100091, China.

[2] Key Laboratory of Biotechnology and Bioresources Utilization, State Ethnic Affairs Commission & Ministry of Education, Dalian Minzu University, Dalian 116600, China.

[3] National Demonstration Center for Experimental Fisheries Science Education, Key Laboratory of Exploration and Utilization of Aquatic Genetic Resources (Ministry of Education) and International Research Center for Marine Biosciences (Ministry of Science and Technology), Shanghai Ocean University, Shanghai 201306, China.

[4] State Key Laboratory of Tree Genetics and Breeding, Northeast Forestry University, Harbin 150040, China.

[5] Key Laboratory of Saline-alkali Vegetation Ecology Restoration (SAVER), Ministry of Education, Alkali Soil Natural Environmental Science Center (ASNESC), Northeast Forestry University, Harbin, China

[6] Beijing ABT Biotechnology Co., Ltd., Beijing 102200, China.

[7] Zhangwu Deya yellowhorn Professional Cooperatives, Zhangwu 123200, China.

*Correspondence address: Libing Wang, State Key Laboratory of Tree Genetics and Breeding, Research Institute of Forestry, Chinese Academy of Forestry, Beijing 100091, China; E-mail: wlibing@caf.ac.cn; Chengjiang Ruan, Key Laboratory of Biotechnology and Bioresources Utilization, State Ethnic Affairs Commission & Ministry of Education, Dalian Minzu University, Dalian 116600, China; E-mail: ruan@dlnu.edu.cn.

[†]These authors contributed equally to this article.

Abstract

Background: Yellowhorn (*Xanthoceras sorbifolium*) (NCBI Taxonomy ID: 99658) is a species of the Sapindaceae family native to China and is an oil tree that can withstand cold and drought conditions. A pseudomolecule-level genome assembly for this species will not only contribute to understanding the evolution of its genes and chromosomes, but also bring yellowhorn breeding into the genomic era.

Findings: Here, we generated 15 pseudomolecules of yellowhorn chromosomes, on which 97.04% of scaffolds were anchored, using the combined Illumina HiSeq, PacBio Sequel and Hi-C technologies. The length of the final yellowhorn genome assembly was 504.2 Mb with a contig N50 size of 1.04 Mb and a scaffold N50 size of 32.17 Mb. Genome annotation revealed that 68.67% of the yellowhorn genome was composed of repetitive elements. Gene modelling predicted 24,672 protein-coding genes. By comparing orthologous genes, the divergence time of yellowhorn and its close sister species longan (*Dimocarpus longan*) was estimated at approximately 33.07 million years ago. Gene cluster and chromosome synteny analysis demonstrated that the yellowhorn genome shared a conserved genome structure with its ancestor in some chromosomes.

Conclusions: This genome assembly represents a high-quality reference genome for yellowhorn. Integrated genome annotations provide a valuable dataset for genetic and molecular research in this species. We did not detect whole-genome duplication in the genome. The yellowhorn genome carries syntenic blocks from ancient chromosomes. These data sources will enable this genome to serve as an initial platform for breeding better yellowhorn cultivars.

Keywords: *Xanthoceras sorbifolium*, yellowhorn, PacBio sequencing, genome assembly, Hi-C, genome annotation, conserved chromosome

Data description

Background

Yellowhorn (*Xanthoceras sorbifolium*) is a woody oil species [1] that belongs to the Sapindaceae family and the monotypic genus *Xanthoceras*. As an endemic and economically important species in Northern China, it is widely used for soil and water conservation due to its capacity to survive on arid, saline, and alkaline land and in extreme temperatures even below −40°C [2, 3]. Almost 7.5×10^5 tons of yellowhorn seeds are harvested in autumn every year [4] (**Fig. 1**). The oil content of its seed kernels can be as high as 67%, of which 85%–93% is unsaturated fatty acid, including 37.1%–46.2% linoleic acid and 28.6%–37.1% oleic acid, which are essential fatty acids in the human diet [5]. Recently, as a major woody oil plant species, yellowhorn has drawn governmental and popular attention because of the shortage of vegetable oil resources in China. Notably, an essential nutrient for brain growth and maintenance—nervonic acid, which is rarely found in plants—accounts for 3.04% of the seed oil of yellowhorn [6, 7]. Recent results indicate that xanthoceraside, a novel triterpenoid saponin extracted from yellowhorn husks, has an antitumor effect and the potential to treat Alzheimer's [8-10]. In this study, we generated a high-quality yellowhorn genome assembly and conducted annotation and genomic structure and evolution analyses. Our data provide a rich resource of genetic information for developing yellowhorn resources and understanding the special place of *Xanthoceras* and Sapindaceae in plant evolution.

Sequenced individual and sample collection

Tender leaves were collected from an individual of *X. sorbifolium* cv. Zhongshi 4, which is a new variety issued by the National Forestry and Grassland Administration (Variety rights No. 20180121), in Zhangwu, Liaoning, China. This tree was produced via clone of a plus tree from natural population in Tongliao, Inner Mongolia, China. The leaves were frozen in liquid nitrogen and stored at −80°C until DNA extraction.

Estimation of genome size through flow cytometry analysis

One-month-old leaves from the sequenced yellowhorn individual were subjected to flow cytometry analysis to estimate the genome size as described by Galbraith [11]. *Glycine max* var. William 82 (2C genome size=2.28 pg) [12-13] and *Populus trichocarpa* var. Nisqually 1 (2C genome size=0.99 pg) [14] were used as standard references. The soybean and yellowhorn samples were chopped together using a razor blade and the nuclei were stained with propidium iodide. To avoid peaks that were too close to be distinguished when run simultaneously, the poplar and yellowhorn samples were run separately. Each sample was measured three times on the flow cytometer. Over 3,000 nuclei were analysed per sample with a FACSAria flow cytometer (Becton Dickinson and Company, NJ, USA). A total of 16 samples were analysed using soybean and poplar as standard species. The software BDFACSDiva (version 8.0.1) was used for data analysis with the coefficient of variation controlled at 5%. Compared with the soybean internal standard (peak at 25,413) and poplar reference (peak at 10,363), the peak fluorescence intensity values of yellowhorn samples were 11,968 and 11,558, respectively. Referencing the soybean genome size (1,115 Mb) and poplar genome size (485±10 Mb) [13-15], the yellowhorn genome size was estimated to be approximately 525.94 Mb and 540.93 Mb, which were relatively close (**Fig.2a**).

Illumina short-read sequencing and heterozygosity analysis

DNA was extracted from the leaves of the same individual using a DNA Secure Plant Kit (TIANGEN, China). The DNA concentration and quality were assessed by 1% agarose gel electrophoresis and with a 2.0 Fluorometer (Life Technologies, CA, USA). One shotgun library with an insert size of 350 bp was prepared using a NEB Next® Ultra DNA Library Prep Kit (NEB, USA). A total of 34.51 Gb raw sequencing data were generated by the Illumina HiSeq X Ten sequencing platform. Primary data analysis was carried out using the standard Illumina pipeline [16]. Short reads were processed with Trimmomatic version 0.33 (Trimmomatic, RRID:SCR_011848) [17,18] and Cutadapt (version 1.13) [19] to remove adapters, leading and trailing bases with a quality score below 20, and reads with an average

per-base quality of 20 over a 4 bp sliding window. Trimmed reads <70 nucleotides long were discarded. Finally, 34.40 Gb clean reads were used for the following analysis and error correction of PacBio reads.

K-mer analysis was performed to estimate the genomic characteristics as mentioned by Marçais [20]. After filtering out low-quality, duplicate and contaminating reads from 34.4 Gb Illumina sequencing data, 21.17 Gb high-quality clean reads were used to generate a *K*-mer (*K* = 17) depth distribution curve using Jellyfish (v2.1.1) (with the parameters -m 17 -t 10 –s 550M) and GCE v1.0.0 [21]. The frequency of 17-mer occurrence (17-mer depth) and the frequency of those 17-mers' species at a given sequencing depth were counted and drawn distribution curves of *K*-mer frequency **(Fig.2b).** Based on the flow cytometry results and computational method [21-22], the middle peak (~34×) was homogzygous peak. The left peak of 17× was heterozygous peak and the right tiny peak (66×) observed in Fig.2b was caused by repeat sequences. Depending on the formula reported by Liu [21], the heterozygosity was estimated at approximately 0.75%.

PacBio SMRT sequencing

Genomic DNA (gDNA) was extracted following the ~40 kb SMRTbell™ Libraries Protocol (https://www.pacb.com/wp-content/uploads/Procedure-Checklist-Preparing-Greater-Than-30-kb-SMRTbell-Libraries-Using-Needle-Shearing-and-BluePippin-Size-Selection-on-Sequel-and-RSII-Systems.pdf). The DNA was purified with a Mobio PowerClean® Pro DNA Clean-Up Kit and its quality was assessed by standard agarose gel electrophoresis and Thermo Fisher Scientific Qubit Fluorometry. The genomic DNA was sheared to a size range of about 40 kb using g-TUBE (Covaris) and 0.45 × AMPure beads were used to enrich and purify large fragments of DNA. Damaged DNA and ends were enzymatically repaired as recommended by Pacific Biosciences. Following this procedure, hairpin adapters were ligated using a blunt-end ligation reaction. The remaining damaged DNA fragments and fragments without adapters at both ends were digested using exonuclease. Subsequently, the resulting SMRTbell templates were purified by Blue Pippin electrophoresis (Sage Sciences) and sequenced on a PacBio RS II instrument using P6-C4

sequencing chemistry. A primary filtering analysis was performed on the sequencer, and the secondary analysis was performed utilizing the SMRT analysis pipeline version 2.1.0 (Pacific Biosciences). In total, we generated 66.44 Gb (roughly 122.83-fold coverage of the yellowhorn genome) of single-molecule sequencing data (6,105,692 PacBio post-filtered reads), with an average read length of 10,882 bp (**Fig. S1; Table S1**).

Genome assembly

After stringent filtering and correction steps using *k*-mer frequency-based methods [23], we assembled contigs using the PacBio reads. Preliminary assembly with the assembler Falcon v0.7 (Falcon, RRID:SCR_016089) (https://github.com/PacificBiosciences/FALCON/wiki/Manual) (falcon_sense_option = --output_multi --min_idt 0.70 --min_cov 4 --max_n_read 300 --n_core 8 overlap_filtering_setting = --max_diff 100 --max_cov 100 --min_cov 2 --n_core 12 --bestn 10) generated a total length of 598.65 Mb of contigs with a N50 length of 1.11 Mb, using the 66.44 Gb PacBio long reads. The software Quiver (based on pbsmrtpipe.pipelines.sa3_ds_resequencing in smrtlink_5.0.1; http://pbsmrtpipe.readthedocs.io/en/master/getting_started.html) was used to polish the PacBio consensus sequence clusters. The assembly was corrected with Pilon version 1.22 (Pilon, RRID:SCR_014731) (https://github.com/broadinstitute/pilon/wiki) using the Illumina short reads. Finally, heterozygous sequences were identified and removed using the Purge Haplotigs pipeline, with the parameters -a 75 (https://bitbucket.org/mroachawri/purge_haplotigs) [24]. Contigs from organelle DNA sources can also be identified and filtered out when the processing with Purge Haplotigs. After the heterozygous sequences were removed, a final assembly from the PacBio reads (504.20 Mb) was generated (**Table 1**).

Pseudomolecule construction and three-dimensional chromatin conformation analysis

The Hi-C technology is an efficient strategy for pseudomolecule construction and enables the generation of genome-wide three-dimensional chromosome architectures. We constructed Hi-C fragment libraries of 350 bp and

sequenced them using the Illumina Hi-Seq platform (Illumina, San Diego, CA, USA) for chromosome pseudomolecule construction. Mapping of the Hi-C reads and assignment to restriction fragments were performed as described in Burton [25]. A total of 53.39 Gb of trimmed reads, representing around 98.70-fold coverage of the yellowhorn genome, were mapped to the assembly with the aligner BWA version 0.7.10 (BWA, RRID:SCR_010910; parameters: bwa index -a bwtsw fasta bwa aln -M 3 -O 11 -E 4 -t 2 fq1 bwa aln -M 3 -O 11 -E 4 -t 2 fq2) [26]. Only uniquely aligned reads with high alignment quality (>20) were selected for pseudomolecule construction. Duplicate removal and quality assessment were performed using HiC-Pro (version 2.8.1) with the following parameters: mapped_2hic_fragments.py -v -S -s 100 -l 1000 -a -f -r -o [27]. In total, 50.56% of the Hi-C data were grouped into valid interaction pairs. A total of 2,836 contigs (N50 length at 1.04 Mb) were assembled after error correction. LACHESIS (parameters: cluster_min_re_sites=48; cluster_max_link_density=2; cluster_noninformative_ratio =2; order_min_n_res_in_trun=14; order_min_n_res_in_shreds=15) [25] was used to assign the order and orientation of each group, with a scaffold N50 of 32.17 Mb.

Using the 98.70-fold coverage of Hi-C reads, 489.28 Mb (97.04%) of the assembly were anchored onto the 15 pseudomolecules, which were in agreement with the yellowhorn karyotype (2n=30) identified by Li [28]. The assembly (477.59 Mb, 94.76%) was ordered by the frequency distribution of valid interaction pairs (**Table 2, Fig. S2**). The coverage of the assembly reached 93.96% and the ratio of unclosed gaps was 0.15‰ (**Table 1**). The assembly was of sufficient quality to be used as a reference for studying yellowhorn biology and plant genomics.

Transcriptome sequencing

RNA was extracted from four tissues (flowers, leaves and roots) of the same individual used for DNA sequencing using the Easy Spin RNA extraction kit (Sangon Biotech, Shanghai, China; No. SK8631). The concentration of each RNA sample was checked using a NanoDrop spectrophotometer (Thermo Fisher Scientific Inc., USA) and a QUBIT® Fluorometer (Life Technologies). The RNA integrity was checked using a Bioanalyzer 2100 (Agilent Technologies).

Iso-Seq libraries were prepared according to the Isoform Sequencing protocol (Iso-Seq) using the Clontech SMARTer PCR cDNA Synthesis Kit and the BluePippin Size Selection System protocol as described by Pacific Biosciences (PN 100-092-800-03). A mixed sample was sequenced on the Pacific Biosciences RS II platform using P6-C4 chemistry.

The sequence data were processed using the SMRTlink 4.0 software. Circular consensus sequences were derived from the subread BAM files with the parameters: min_length 200, max_drop_fraction 0.8, no_polish TRUE, min_zscore -999, min_passes 1, min_predicted_accuracy 0.8, max_length 18000. Separation of the full length and non-full length reads was conducted using pbclassify.py (ignorepolyA false, minSeqLength 200). The non-full length and full length fasta files produced were then fed into the cluster step to cluster the isoforms, and subjected to final Arrow polishing with the parameters hq_quiver_min_accuracy 0.99, bin_by_primer false, bin_size_kb 1, qv_trim_5p 100, qv_trim_3p 30. The LoRDEC software (version 0.3) was used to correct sequencing errors in the consensus transcripts using the Illumina reads as a reference (parameters: -k 19 -s 3) [29]. The corrected consensus transcripts were clustered using CD-HIT (version 4.6.8) (-c 0.99 -T 6 -G 0 -aL 0.90 -AL 100 -aS 0.99 -AS 30) [30] to reduce sequence redundancy and improve the performance of other sequence analyses.

A total of 110,584 non-redundant unigenes were generated from 142,396 transcripts in the final RNA assemblies, which were used as evidence to assist with gene prediction. Among the 110,584 non-redundant transcripts, 8,466 (7.66%) were non-coding mRNAs. Each gene had an average of 2–7 transcripts, among which the longest transcript representing that gene was kept in the final gene model set.

Evaluation of assembly quality

The completeness of the final assembly was evaluated using CEGMA version 2.5 (CEGMA, RRID:SCR_015055) [31] (http://korflab.ucdavis.edu/dataseda/) and BUSCO version 3.0.2 (BUSCO, RRID:SCR_015008) [32-33] (https://gvolante.riken.jp/analysis.html). The CEGMA outputs showed that 94.76% of the core eukaryotic genes (235 out of 248 core eukaryotic genes) were present in our assembly. The BUSCO test, referencing the embryophyta protein

set (run_BUSCO.py -i plant_species.fa -o plant_species-l embryophyta_odb9/-m proteins), identified 94.7% of plant gene sets as complete (1364 out of 1440 BUSCOs), including 89.0% single-copy and 5.7% duplicated genes (**Table S2**). All of these results suggested a high assembly quality for the yellowhorn genome.

Annotation of repetitive sequences

A *de novo* repeat database was constructed using RepeatScout version 1.0.5 (RepeatScout, RRID:SCR_014653) [34], LTR-FINDER (version 1.0.7) [35], MITE-Hunter (version 1.0) [36] and PILER (version 1.0) with default parameters [37]. The predicted repeats were classified using PASTEClassifier (version 1.0) with default parameters [38-39]. Then, RepeatMasker version 4.0.7 (RepeatMasker, RRID:SCR_012954) [40] was used with the following parameters "-nolow -no_is -norna -engine wublast -qq -frag 20000" to identify repeat sequences by aligning them against known gene and genome sequences, based on Repbase (version 19.06) [41] and the *de novo* repeat database.

The predicted repeats represented 346.39 Mb (68.67%) of the yellowhorn genome assembly. Among these repeats, two types of LTR-retrotransposons were the most abundant, including 98.68 Mb of *Copia*-type (19.57%) and 88.24 Mb of *Gypsy*-type (17.50%) repeats (**Table S3**). Accumulation of LTR-retrotransposons is an important contributor to genome expansion and diversity [42]. The insertion time of LTR-retrotransposons in the genome was estimated by calculating the sequence variance between the LTR arms of each LTR-retrotransposon, using a substitution rate of 1.3×10^{-8} substitutions per site per year [43]. To calculate the insertion age of each LTR retrotransposon, the 5′ and 3′ LTRs of each element were aligned with MUSCLE version 3.8.31 (MUSCLE, RRID:SCR_011812) using default setting parameters [44] (https://www.ebi.ac.uk/Tools/msa/muscle/). Distmat (with default parameters) was used to estimate the DNA divergence between the LTR sequences with the Kimura-2-parameter base substitution model [45] and DNA divergence was converted to divergence time. A comparison of the insertion ages for LTR-retrotransposons showed similar insertion profiles among the genomes of clementine [46] (annotation version 1.0), longan [47] (annotation version 1.0), grape [48] (*V. vinifera*, annotation version GenomeScope.12X) and yellowhorn **(Fig. 3a)**. We

observed that the yellowhorn genome carried more young LTR-retrotransposons, with the highest proportion of LTR-retrotransposons with insertion ages less than 0.2 million years ago (mya). This might have resulted from rapid changes of its growing environment, such as the effects of pathogens and interference from human activities in recent years. The genomes sequenced by pure next-generation sequencing technology might show less LTR-retrotransposons because the sequence similarity between LTR arms and among different LTR-retrotransposons probably caused assembly errors in these regions, which may have led to underestimation of the LTR-retrotransposons in clementine and longan. Comparison of the insertion ages suggested a similar insertion age between *Copia*-type and *Gypsy*-type LTR-retrotransposons (**Fig. S3**).

Prediction of protein-coding genes

Annotation of protein-coding genes in the yellowhorn genome was conducted by combining *de novo* prediction, homology information, and RNA-seq data. For the *de novo* prediction, Genscan (version 3.1) [49], Augustus (Augustus: Gene Prediction, RRID:SCR_008417) (version 3.1) [50], GlimmerHMM version 3.0.4 (GlimmerHMM, RRID:SCR_002654) [51], GeneID (version 1.4) [52], and SNAP (version 2006-07-28) [53] were used to analyse the repeat-masked genome with default parameters. For the similarity-based prediction, the Uniprot protein sequences from three sequenced plants, Arabidopsis (TAIR 10, http://brassicadb.org/brad/datasets/pub/BrassicaceaeGenome/Arabidopsis_thaliana/), longan (V1.0, http://gigadb.org/dataset/100276) and grape (Genomescope 12×, https://www.ncbi.nlm.nih.gov/genome/?term=Vitis+vinifera+genome), were aligned against the *ab initio* gene models in the yellowhorn genome using GeMoMa (version 1.3.1) [54]. When multiple transcripts were predicted at the same location, the highest GeMoMa scoring transcript was chosen as the optimal model [55]. The RNA-seq data were aligned to the reference genome with PASA (version 2.0.2) [56] under default parameters. All predictions from the three methods were combined with EVidenceModeler (v1.1.1) (Mode:STANDARD S-ratio: 1.13 score>1000) [57] to

produce a consensus gene set. During the EVM integration, higher weights were assigned to the predicted PASA and GeMoMa models than the *ab initio* models. PASA was used to modify the final gene models.

The RNA-seq reads were then aligned to the yellowhorn genome assembly with TopHat (TopHat, RRID:SCR_013035) (v2.0.10, implemented with bowtie2) [58] to identify candidate exon regions and splicing donor and acceptor sites to evaluate the gene prediction results. Infernal version 1.1 (Infernal, RRID:SCR_011809) (default parameters) [59] was used to identify non-coding rRNA and microRNA genes based on Rfam (version 12.1) [60] and miRbase (version 21) [61]. TRNAscan-SE (version 1.3.1) (default parameters) [62] was used to identify tRNA genes.

GenBlastA v1.0.4(-e 1e-5) was used to perform pseudogene prediction by scanning the yellowhorn genome for sequences homologous to the known protein-coding genes it contained, and premature stop codons or frame shift mutations in those sequences were identified by GeneWise version 2.4.1 (GeneWise, RRID:SCR_015054) with the parameters: -both -pseudo [63-64].

Functional annotation of the protein-coding genes was carried out by searching against the NR, KOG, GO, KEGG, and TrEMBL databases. Additionally, the gene models were aligned to the Pfam database using Hmmer version 3.0 (Hmmer, RRID:SCR_005305) (parameters, -E 0.00001 --domE 0.00001 --cpu 2 --noali –acc)] [64-70]. GO terms were allocated to the genes using the Blast2GO version2.2.31 (Blast2GO, RRID:SCR_005828) pipeline [70].

In total, we predicted 24,672 protein-coding genes (**Table S4**) and 1,913 pseudogenes, with an average gene length of 4,199 bp, average intron length of 2,560 bp and average coding sequence length of 1,580 bp. Of these genes, 99.02% (24,429) carried at least one conserved functional domain (**Table S5**). Their functions were classified using GO terms (**Fig. S4**) and the KOG database (**Fig. S5**). For the non-coding mRNA genes, 642 tRNA, 108 microRNA and 316 rRNA genes were predicted in the yellowhorn genome.

Chromosome synteny between the yellowhorn and reference genomes

To investigate the evolution of the yellowhorn chromosomes, gene collinearity was determined by anchoring the

aligned yellowhorn genes to the reference genomes of clementine, Arabidopsis and grape using the Multiple Collinearity Scan toolkit (MCscan) (version 0.8) [71]. The parameters of the MCscan alignment were as follows: $/MCScanX xxx.blast$-s 10 --b $2 (inter-species) blastp -query b.fa -db adb -out xyz.blast -evalue 1e-10 -num_threads 16 -outfmt 6 -num_alignments 5. A total of 367, 409 and 386 syntenic blocks were identified on the basis of the orthologous gene orders, corresponding to 28,372, 18,650 and 23,400 genes in each genome, respectively. The average gene number per block was 77.3, 45.6 and 60.6 genes, respectively. This suggested that yellowhorn and clementine shared the highest collinearity, which was consistent with their close phylogenetic relationship as members of the Sapindales clade. The alignments of syntenic chromosomes were visualized between yellowhorn and the other genomes. The frequency of large-scale fragment rearrangements between yellowhorn and clementine, including inversions and translocations, was considerably lower than between yellowhorn and the other two genomes (**Fig. 4**). In particular, structural variation between yellowhorn and grape was so frequent that it was too difficult to speculate on the syntenic relationships among the chromosomes (**Fig. 4b**). The chromosome alignments between yellowhorn linkage groups and clementine pseudomolecules revealed that most of the cross-chromosome rearrangements were different from those between yellowhorn and Arabidopsis (**Fig. 4d, 4e**). Yellowhorn Linkage groups 2 and 11 were found to be syntenic to single clementine pseudomolecules, Scaffold 5 and 3, respectively, and Linkage groups 3, 4, 5, 7, 8, 10, 12, 14 and 15 were each aligned to two reference chromosomes of clementine. Comparatively, frequency of chromosome rearrangement was a little higher between the yellowhorn linkage groups and Arabidopsis chromosomes. Arabidopsis Chromosome 1 was predominantly syntenic to yellowhorn Linkage group 4, which demonstrated that the yellowhorn genome contained some conserved genome structure from its originals (**Fig. 4d**). Intriguingly, similar chromosomal fusion events were found among some chromosomes. Aligned fragments of Arabidopsis Chromosomes 1, 3 and 5 were fused to form yellowhorn linkage groups 1 and 14, similarly to clementine Scaffolds 1, 2 and 3. Yellowhorn Linkage group 6 was aligned to clementine scaffolds 1, 3, 4 and 6, but had extensive collinearity with Arabidopsis Chromosome 3 (**Fig. 4d, 4e**). However, phylogenetic analysis suggested a distant relationship between Arabidopsis and yellowhorn.

These findings suggested that Arabidopsis and yellowhorn share a chromosome of their origins, despite extensive rearrangements. Overall, these findings shed new light on the evolution of eudicot plant chromosomes.

Identification of gene clusters and duplication

Gene clustering was conducted using OrthoMCL version 5 (OrthoMCL DB: Ortholog Groups of Protein Sequences, RRID:SCR_007839, parameters: Pep_length 10 Stop_coden 20 PercentMatchCutoff 50 EvalueExponentCutoff -5 Mcl 1.5 #1.2~4.0) [72] among the protein sequences of 10 high-quality typical eudicot genomes representative of important families, including *D. longan* (Sapindaceae, Sapindales) [46], *Citrus clementina* (Rutaceae, Sapindales) [47], *Brassica rapa* (Brassicaceae, Brassicales), *Arabidopsis thaliana* (Brassicaceae, Brassicales) [73-74], *Theobroma cacao* (Sterculiaceae, Malvales) [75], *Gossypium raimondii* (Malvaceae, Malvales) [76], *Quercus robur* (Fagaceae, Fagales) [77], *Vitis vinifera* (Vitaceae, Vitales) [78], *Cucumis sativus* (Cucurbitaceae, Cucurbitales) [79] and *Malus* × *domestica* (Rosaceae, Rosales) [80], as well as yellowhorn (Additional file: Table S6). The yellowhorn genes were clustered into a total of 14,828 families, including 169 yellowhorn-specific gene families (Additional file: Table S7). Comparison of gene copy numbers among the 11 eudicot genomes indicated that the yellowhorn genome had a similar proportion of single and multiple copy genes to the other analysed genomes (**Fig. 3b**). Intriguingly, the species-specific genes of yellowhorn were similar to those of *T. cacao*, which implied that the yellowhorn genes might have conserved the similar gene structure with their origins.

Over 300 one-to-one single-copy genes shared by all 11 genomes were identified and used to construct a phylogenetic tree using PHYML (version 3.0) (**Fig. 3c**) [81]. The TIM2+I+G model was used to construct the evolutionary tree as determined by jmodeltest. The software Muscle (version 3.8.31) (https://www.ebi.ac.uk/Tools/msa/muscle/) [44] was used to align the orthologs. The alignment outputs were treated with Gblocks (version 14.1) with the parameters -t = p -b5 = h -b4 = 5 -b3 = 15 -d = y -n= y [82]. Divergence times were estimated using MCMCtree (version 4.7a) (http://abacus.gene.ucl.ac.uk/software/paml.html) [83] with the

parameters: burn-in=10,000, sample-number=100,000, sample-frequency=2. The TimeTree database (http://www.timetree.org/), r8s (parameter: r8s -b -f r8s_in.txt > r8s_out.txt) and divergence time (Whelan [84] and Yang [85]) were used to calibrate the time. The fossil calibration times used in the evolutionary trees were as follows: (((Qrob,(Csat,Mdom)),((Ccle,(Xsor,Dlon)),((Tcac,Grai),(Brapa,Atha)'<30.9>20.4'))),Vvin)'<115>105'. The credibility intervals for the divergence time estimates were as follows: UTREE 1 = (((Qrob: 93.929608, (Csat: 83.608799, Mdom: 83.608799) [&95%={67.268, 96.218}]: 10.320809) [&95%={78.104, 105.034}]: 9.748170, ((Ccle: 64.380901, (Xsor: 33.069679, Dlon: 33.069679) [&95%={18.376, 48.565}]: 31.311222) [&95%={46.354, 81.164}]: 27.870851, ((Tcac: 38.243394, Grai: 38.243394) [&95%={21.870, 56.407}]: 43.965024, (Brapa: 26.409279, Atha: 26.409279) [&95%={20.721, 30.886}]: 55.799139) [&95%={67.279, 94.364}]: 10.043334) [&95%={77.382, 103.299}]: 11.426026) [&95%={89.679, 113.000}]: 6.145826, Vvin: 109.823604) [&95%={104.966, 114.982}]. Yellowhorn and longan in the Sapindaceae family showed the closest relationship, with the divergence time estimated at approximately 33.07 mya. Using the orthologous gene pairs of yellowhorn and longan identified by gene collinearity and paralogous pairs identified by gene clustering, 4DTv (four-fold degenerate synonymous sites of the third codons) values were calculated for all of the duplicated pairs. A species divergence peak (4DTv~0.1) was observed in the yellowhorn vs. longan ortholog 4DTv distribution but no obvious peak could be seen in the yellowhorn and longan paralog curves (**Fig. 3d**). In a self-alignment of the chromosomes based on gene synteny, no large-scale gene duplications were found in the yellowhorn genome (**Fig. S2**), suggesting that the yellowhorn genome has not undergone whole-genome or large-fragment duplication.

List of Abbreviations:

bp: base pair; BUSCO: Benchmarking Universal Single-Copy Ortholog; CDS: coding sequence; GO: Gene Ontology; kb: kilobases; KEGG: Kyoto Encyclopedia of Genes and Genomes; LTR: long terminal repeat; Mb: megabases; Mya: million years ago; NCBI: National Center for Biotechnology Information; PE: paired-end; RNA-Seq: RNA sequencing;

SMRT: Single-Molecule Real-Time; SRA: Sequence Read Archive.

Additional File

Additional file 1: Tables S1 to S7

Table S1: PacBio data statistics.

Table S2: Genome quality assessed by the BUSCO test.

Table S3: Repetitive sequence content.

Table S4: Prediction of protein-coding genes.

Table S5: Function annotation of protein-coding genes.

Table S6: Data used in orthoMCL analysis.

Table S7: Annotation and locus information of 169 yellowhorn-specific gene families.

Additional file 2: Figures S1 to S5

Figure S1: Length distribution of the three types of PacBio reads produced.

Figure S2: Interaction frequency distribution of Hi-C links among chromosomes.

Figure S3. Distribution of insertion ages of *Copia*-type and *Gypsy*-type LTR-retrotransposons.

Figure S4: Function classification of protein-coding genes against the GO term database.

Figure S5: KOG function classification of protein-coding genes.

Funding

This work was financially supported by the Central Public-Interest Scientific Institution Basal Research Fund

(CAFYBB2019QD001), the National “12th Five-Year” Plan for Science & Technology Support of China (2015BAD07B0106), the National Natural Science Foundation of China (31800571, 31870594, 31760213) and the National Key Research and Development Plan of China (2016YFC050080506).

Availability of supporting data

The raw sequence data have been deposited in NCBI under project accession number PRJNA483857. The Biosample numer was SAMN09748200. The Short Read Archive (SRA) accession number of trasncriptome sequencing, PacBio SMRT sequencing, Illumina short-read sequencing and Illumina sequencing for Hi-C was SRR7768197, SRR7768198, SRR7768199 and SRR7768201, respectively (in SRP159119). The accession number of *Xanthoceras sorbifolium* Genome sequencing and assembly was QUWJ 00000000. All supplementary figures and tables are provided in Additional Files. Additional supporting data, including the genome assembly, annotations and phylogenetic tree files, are available via the GigaScience database GigaDB [86].

Conflict of Interest

The authors declare that they have no competing financial interests.

Author Contributions

QXB, HYY, YL, CJR and LBW conceived and designed the study; TPC, XJL, YCL, SQF, XYH, GHF, YFC, JD, DSC, ZMZ and ZL prepared materials and conducted the experiments; QXB, YZ, WD, YL and LG wrote the manuscript.

Legend

Fig. 1 Images of yellowhorn plants. (**a**) Yellowhorn tree in an artificial forest. (**b**) Ripe fruit, which dehisce into three parts by the carpels. (**c**) A harvest scene of yellowhorn in northern China. (**d**) Seeds in ripe fruits, which number 18–24 in one fruit.

Fig. 2 Estimation of genome size. (**a**) Test results of yellowhorn, poplar and yellowhorn + soybean samples using flow cytometry. (**b**) Distribution of 17-mer frequency. The *x*-axis and *y*-axis indicate the 17-mer frequency and number, respectively. The leftmost truncated peak at a low occurrence frequency (1–2) was mainly due to random base errors in the raw sequencing reads.

Fig. 3 Genome evolution. (**a**) Distribution of insertion ages of LTR-retrotransposons. The *x*-axis represents the estimated insertion age (mya) of the LTR-retrotransposons. The *y*-axis represents the number of intact LTR-retrotransposons. (**b**) Comparison of copy numbers in gene clusters of analysed eudicot genomes. According to the identified gene clusters, the genes were grouped into single-copy, multiple-copy and species-specific (specific) genes. (**c**) Constructed phylogenetic tree and divergence time estimation. The black numbers represent estimated divergence times (mya), which are measured with a scale bar of 20 million years, and green numbers represent bootstrap values. Grape (*V. vinifera*) was used as an outgroup. (**d**) Genome duplication in eudicot genomes as revealed through 4DTv analyses. The percentages of the orthologous pairs (Y vs. L) between yellowhorn (Y) and longan (L) and paralogous gene pairs within the yellowhorn (Y vs. Y) and longan (L vs. L) genomes are plotted against their calculated 4DTv values.

Fig. 4 Chromosome synteny. The circularized blocks represent the chromosomes of yellowhorn and other genomes. Aligned genes identified by MCscanX are connected by lines, with their chromosome locations shown in different colours. (**a**) Chromosome alignment of yellowhorn and Arabidopsis. (**b**) Chromosome alignment of yellowhorn and grape. (**c**) Chromosome alignment of yellowhorn and clementine. Coloured ribbons connect the aligned genes. Yellowhorn linkage groups are labelled LG 1 to 15, Arabidopsis chromosomes labelled Chr 1 to 5, grape chromosomes are labelled C1 to 19 and CUn (chromosome location unknown) and clementine scaffolds are labelled Sc 1 to 9. Scale, 10 Mb. (**d**) Chromosome rearrangements between Arabidopsis and yellowhorn. (**e**) Chromosome rearrangements

between clementine and yellowhorn. Arabidopsis and clementine chromosomes are represented as bars of different colours. Synteny and rearrangement of the yellowhorn chromosomes are indicated by different blocks, corresponding to the reference Arabidopsis and clementine chromosomes.

Editor's Note

Please also note another, independent Data Note published in *GigaScience*, also presenting a genome assembly of *Xanthoceras sorbifolium* [87]. We independently received two submissions on the Yellowhorn genome, from two different teams, within a short period of time. We reviewed both submissions in parallel and decided to publish them "back-to-back" in the journal, on the same day.

References

[1] Wang Q, Yang L, Ranjitkar S et al. Distribution and in situ conservation of a relic Chinese oil woody species yellowhorn *Xanthoceras sorbifolium* Bunge. Can J For Res 2017; 47: 1450-6.

[2] Board E. Flora of China vol.47. 1985; 47: 72.

[3] Yu HY, Fan SQ, Bi QX et al. Seed morphology, oil content and fatty acid composition variability assessment in yellow horn (*Xanthoceras sorbifolium* Bunge) germplasm for optimum biodiesel production. Ind Crop Prod 2017; 97: 425-30.

[4] Yao ZY, Qi JH, Yin LM, et al. Biodiesel production from *Xanthoceras sorbifolia* in China: Opportunities and challenges. Renew Sust Energy Rev 2013; 24: 57-65.

[5] Venegas-Calerón M, Ruíz-Méndez MV, Martínez-Force E et al. Characterization of *Xanthoceras sorbifolium* Bunge seeds: Lipids, proteins and saponins content. Ind Crop Prod 2017; 109: 192-8.

[6] Krishnan H. Modification of seed composition to promote health and nutrition. Crop Sci Soc Amer 2009: 263-71.

[7] Taylor DC, Guo Y, Katavic V et al. New Seed Oils for Improved Human and Animal Health: Genetic Manipulation of the Brassicaceae for Oils Enriched in Nervonic Acid. Modification of Seed Composition to Promote Health and Nutrition 2009: 51.

[8] Qi Y, Ji XF, Chi TY et al. Xanthoceraside attenuates amyloid β peptide 1-42 -induced memory impairments by reducing neuroinflammatory responses in mice. Eur J Pharmacol 2017; 820: 18-30.

[9] Ji XF, Chi TY, Liu P et al. The total triterpenoid saponins of *Xanthoceras sorbifolia* improve learning and memory impairments through against oxidative stress and synaptic damage. Phytomedicine 2017; 25: 15-24.

[10] Zhang Y, Xiao LU, Xiao B et al. Research progress and application prospect of *Xanthoceras sorbifolia* for treating Alzheimer's disease. Drug Eval Res 2018; 25: 912-7.

[11] Galbraith DW, Harkins KR, Maddox JM et al. Rapid flow cytometric analysis of the cell cycle in intact plant tissues. Science 1983; 220: 1049-51.

[12] Pellicer J and Leitch I J. The Application of Flow Cytometry for Estimating Genome Size and Ploidy Level in Plants. Methods mol biolo (Clifton, N.J.) 1115:279-307.

[13] Schmutz J, Cannon SB, Schlueter J, et al. Genome sequence of the palaeopolyploid soybean. Nature 2010; 463:178-83.

[14] Tuskan GA, Difazio S, Jansson S, et al. The genome of black cottonwood, *Populus trichocarpa* (Torr. & Gray). Science 2016; 313: 1596-604.

[15] Dolezel J, Greilhuber, J, Suda, J, et al. Estimation of nuclear DNA content in plants using flow cytometry. Nat Protoc 2007; 2: 2233-44.

[16] Toh H, Shirane K, Miura F, et al. Software updates in the Illumina HiSeq platform affect whole-genome bisulfite sequencing. BMC Genomics 2017; 18: 31.

[17] Alberto CM, Sanso AM, Xifreda CC et al. Chromosomal studies in species of Salvia (Lamiaceae) from Argentina. Bot J Linn Soc 2015; 141: 483-90.

[18] Bolger AM, Lohse M, Usadel B et al. Trimmomatic: a flexible trimmer for Illumina sequence data. Bioinformatics 2014; 30: 2114-20.

[19] Martin M. Cutadapt removes adapter sequences from high-throughput sequencing reads. Embnet J 2011; 17: 10-2.

[20] Marçais G, Kingsford C. A fast, lock-free approach for efficient parallel counting of occurrences of k-mers. Bioinformatics 2011; 27: 764-70.

[21] Liu BH, Shi YJ, Yuan JY et al. Estimation of genomic characteristics by analyzing kmer frequency in de novo genome projects. Quant Biol 2013; doi:10.1016/S0925-4005(96)02015-1.

[22] Kajitani R, Toshimoto K, Noguchi H et al. Efficient de novo assembly of highly heterozygous genomes from whole-genome shotgun short reads. Genome Res 2014; 24: 1384-95

[23] Li R, Fan W, Tian G et al. The sequence and de novo assembly of the giant panda genome. Nature 2010; 463: 311-29.

[24] Roach M J, Schmidt S and Borneman A R. Purge Haplotigs: allelic contig reassignment for third-gen diploid genome assemblies. BMC Bioinformatics 2018; 19:460-75.

[25] Burton J N, Adey A, Patwardhan RP et al. Chromosome-scale scaffolding of de novo genome assemblies based on chromatin interactions. Nat Biotechnol 2013; 31: 1119-25.

[26] Li H and Durbin R. Fast and accurate short read alignment with BurrowsZ Wheeler transform. Bioinformatics, 2009; 25: 1754-60.

[27] Servant N, Varoquaux N, Lajoie BR et al. HiC-Pro: an optimized and flexible pipeline for Hi-C data processing. Genome Biol 2015; 16: 259-70.

[28] Li MX. karyotyoe analysis of some oil plants. Acta Botanica Boreali-Occidentalia Sinica 1987; 7: 246-51.

[29] Salmela L, Rivals E. LoRDEC: accurate and efficient long read error correction. Bioinformatics 2014; 30: 3506-14.

[30] Fu L, Niu B, Zhu Z et al. CD-HIT: accelerated for clustering the next-generation sequencing data. Bioinformatics 2012; 28: 3150-2.

[31] Parra G, Bradnam K, Korf I et al. CEGMA: a pipeline to accurately annotate core genes in eukaryotic genomes. Bioinformatics 2007; 23: 1061-7.

[32] Simao FA, Waterhouse RM, Ioannidis P et al. BUSCO: assessing genome assembly and annotation completeness with single-copy orthologs. Bioinformatics 2015; 31: 3210-2.

[33] Nishimura O, Hara Y, Kuraku S. Volante for standardizing completeness assessment of genome and transcriptome assemblies. Bioinformatics 2017; 33: 3635-37.

[34] Price AL, Jones NC, Pevzner PA et al. De novo identification of repeat families in large genomes. Bioinformatics 2005; 21: i351-8.

[35] Xu Z, Wang H. LTR_FINDER: an efficient tool for the prediction of full-length LTR retrotransposons. Nucleic Acids Res 2007; 35: 265-8.

[36] Han Y, Wessler SR. MITE-Hunter: a program for discovering miniature inverted-repeat transposable elements from genomic sequences. Nucleic Acids Res 2010; 38: e199.

[37] Edgar RC, Myers EW et al. PILER: identification and classification of genomic repeats. Bioinformatics 2005; 21: i152-8.

[38] Wicker T, Sabot F, Hua-Van A et al. A unified classification system for eukaryotic transposbale elments. Nat rev Genet 2007; 8: 973-82.

[39] Hoede C, Arnoux S, Moisset M, et al. PASTEC: An Automatic Transposable Element Classification Tool. Plos one 2014; 9: e91929.

[40] Tarailo-Graovac M, Chen N. Using RepeatMasker to identify repetitive elements in genomic sequences. Curr Protoc Bioinformatics 2004, p.4-10.

[41] Jurka J, Kapitonov VV, Pavlicek A et al. Repbase Update, a database of eukaryotic repetitive elements. Cytogenet Genome Res 2005; 110: 462-7.

[42] Kidwell MG, Lisch D. Transposable elements as sources of variation in animals and plants. Proc Natl Acad Sci U S

A. 1997; 94: 7704-11.

[43] Zuccolo A, Sebastian S, Yu Y et al. Assessing the Extent of Substitution Rate Variation of Retrotransposon Long Terminal Repeat Sequences in *Oryza sativa* and *Oryza glaberrima*. Rice, 2010; 3: 242-50.

[44] Edgar RC. MUSCLE: multiple sequence alignment with high accuracy and high throughput. Nucleic acids research 2004, 32: 1792-7.

[45] Kimura M. A simple method for estimating evolutionary rates of base substitutions through comparative studies of nucleotide sequences. J Mol Evol 1980; 16: 111-20.

[46] Lin Y, Min J, Lai R et al. Genome-wide sequencing of longan (*Dimocarpus longan* Lour.) provides insights into molecular basis of its polyphenol-rich characteristics. Gigascience 2017; 6: 1-14.

[47] Wu GA, Prochnik S, Jenkins J et al. Sequencing of diverse mandarin, pummelo and orange genomes reveals complex history of admixture during citrus domestication. Nat Biotechnol 2014; 32: 656-62.

[48] Jaillon O, Aury JM, Noel B et al. The grapevine genome sequence suggests ancestral hexaploidization in major angiosperm phyla. Nature 2007; 449: 463-7.

[49] Burge C, Karlin S et al. Prediction of complete gene structures in human genomic DNA. J mol boil 1997; 268: 78-94.

[50] Stanke M, Waack S et al. Gene prediction with a hidden Markov model and a new intron submodel. Bioinformatics 2003; 19: 215-25.

[51] Majoros WH, Pertea M, Salzberg S L et al. Tigr Scan and Glimmer HMM: two open source ab initio eukaryotic gene-finders. Bioinformatics 2004; 20: 2878-9.

[52] Blanco E, Parra G, Guigó R et al. Using geneid to identify genes. Cur Protoc Bioinformatics 2007; 18: 3-4.

[53] Korf I. Gene finding in novel genomes. BMC Bioinformatics 2004; 5: 59-68.

[54] Jens K, Michael W, Erickson J L et al. Using intron position conservation for homology-based gene prediction. Nucleic Acids Res 2016; 44: e89.

[55] Campbell MA, Haas BJ, Hamilton JP et al. Comprehensive analysis of alternative splicing in rice and comparative analyses with Arabidopsis. BMC Genomics 2006; 7: 327-43.

[56] Tang S, Lomsadze A, Borodovsky M et al. Identification of protein coding regions in RNA transcripts. Nucleic Acids Res 2014; 43: 58-68.

[57] Haas BJ, Papanicolaou A, Yassour M et al. De novo transcript sequence reconstruction from RNA-seq using the Trinity platform for reference generation and analysis. Nat Protoc 2013; 8: 1494-512.

[58] Trapnell C, Pachter L, Salzberg S L et al. TopHat: discovering splice junctions with RNA-Seq. Bioinformatics 2009; 25: 1105-11.

[59] Nawrocki EP, Eddy SR et al. Infernal 1.1: 100-fold faster RNA homology searches. Bioinformatics 2013; 29: 2933-5.

[60] Griffiths-Jones S, Moxon S, Marshall M et al. Rfam: annotating non-coding RNAs in complete genomes. Nucleic Acids Res 2005; 33: 121-4.

[61] Griffithsjones S, Grocock RJ, Van DS et al. miRBase: microRNA sequences, targets and gene nomenclature. Nucleic Acids Res 2006; 34: 140-4.

[62] Lowe TM, Eddy SR et al. tRNAscan-SE: a program for improved detection of transfer RNA genes in genomic sequence. Nucleic Acids Res 1997; 25: 955-64.

[63] She R, Chu JK, Pei J et al. GenBlastA: enabling BLAST to identify homologous gene sequences. Genome Res 2009; 19:143-9.

[64] Birney E, Clamp M, Durbin R et al. GeneWise and Genomewise. Genome Res 2004; 14: 988-96.

[65] Marchlerbauer A, Lu S, Anderson JB et al. CDD: a Conserved Domain Database for the functional annotation of proteins. Nucleic Acids Res 2011; 39: 225-9.

[66] Tatusov RL, Natale DA, Garkavtsev IV et al. The COG database: new developments in phylogenetic classification of proteins from complete genomes. Nucleic Acids Res 2001; 29: 22-8.

[67] Dimmer EC, Huntley RP, Alamfaruque Y et al. The UniProt-GO Annotation database in 2011. Nucleic Acids Res 2012; 40: 565-70.

[68] Du JL; Yuan ZF; Ma ZW et al. KEGG-PATH: Kyoto encyclopedia of genes and genomes-based pathway analysis using a path analysis model. Mol Biosystems 2014;10: 2141-7.

[69] Boeckmann B, Bairoch A, Apweiler R et al. The Swiss-Prot knowledgebase and its supplement TREMBL in 2003. Nucleic Acids Res 2003; 31: 365-70.

[70] Altschul S, Gish W, Miller W et al. Basic local alignment search tool. J Mol Biol 1990; 215: 403-10.

[71] Tang HB, Bowers JE, Wang XY, et al. Perspective- Synteny and collinearity in plant genomes. Science 2008; 320: 486-8.

[72] Li L, Jr SC, Roos DS et al. OrthoMCL: identification of ortholog groups for eukaryotic genomes. Genome Res 2003; 13: 2178-89.

[73] Wang XW, Wang HZ, Wang J et al. The genome of the mesopolyploid crop species *Brassica rapa*. Nature Genet 2011; 43: 1035-40.

[74] Theologis A, Ecker JR, Palm CJ et al. Sequence and analysis of chromosome 1 of the plant *Arabidopsis thaliana*. Nature 2000; 408: 816-20.

[75] Argout X, Salse J, Aury JM et al. The genome of *Theobroma cacao*. Nat Genet 2013; 43:101-8.

[76] Wang K, Wang Z, Li F et al. The draft genome of a diploid cotton *Gossypium raimondii*. Nat Genet 2012; 44:1098-103.

[77] Plomion C, Aury JM, Amselem J et al. Oak genome reveals facets of long lifespan. Nat Plants 2017; 4: 440-52.

[78] Jaillon O, Aury JM, Noel B et al. The grapevine genome sequence suggests ancestral hexaploidization in major angiosperm phyla. Nature 2007; 449: 463-7.

[79] Huang S, Li R, Zhang Z et al. The genome of the cucumber, *Cucumis sativus* L. Nat Genet 2009; 41: 1275-81.

[80] Velasco R, Zharkikh A, Affourtit J et al. The genome of the domesticated apple (*Malus* × *domestica* Borkh.). Nat

Genet 2010; 42: 833-9.

[81] Guindon S, Dufayard JF, Lefort V et al. New algorithms and methods to estimate maximum-likelihood phylogenies: assessing the performance of PhyML 3.0. Syst Biol 2010; 59: 307-21.

[82] Talavera G, Castresana J et al. Improvement of phylogenies after removing divergent and ambiguously aligned blocks from protein sequence alignments. Syst Biol 2007; 56: 564-77.

[83] Battistuzzi FU, Billingross P, Paliwal A et al. Fast and slow implementations of relaxed-clock methods show similar patterns of accuracy in estimating divergence times. Mol Biol Evol 2011; 28: 2439-42.

[84] Whelan S, Goldman N. A general empirical model of protein evolution derived from multiple protein families using a maximum likelihood approach. Mol Biol Evol 2001; 18: 691-9.

[85] Yang Z, Nielsen R, Hasegawa M. Models of amino acid substitution and applications to mitochondrial protein evolution. Mol Biol Evol 1998; 15:1600-11.

[86] Bi Q, Zhao Y, Du W, Lu Y, Gui L, Zheng Z et al. Supporting data for "Pseudomolecule-level assembly of the Chinese oil tree yellowhorn (Xanthoceras sorbifolium) genome" GigaScience Database 2019. http://dx.doi.org/10.5524/100606

[87] Liang Q, Li H, Li S, Yuan F, Sun J, Duan Q et al. The genome assembly and annotation of yellowhorn (Xanthoceras sorbifolium Bunge). GigaScience 2019. [PLEASE INSERT DOI HERE].

Table1 Overview of assembly and annotation for the yellowhorn genome.

Total length	504,196,643 bp
Length of unclosed gaps	73,800 bp
N50 length (initial contigs)	1,044,891 bp
N50 length (scaffolds)	32,173,403 bp
N90 length (scaffolds)	25,069,408 bp
Number of scaffolds (＞N90 length)	21
Largest scaffold	40,097,451 bp
GC content	36.95%
Number of predicted protein-coding genes	24,672
Number of predicted noncoding RNA genes	1,066
Content of repetitive sequences	68.67%
Length of genome anchored on linkage groups	489,286,946 bp (97.04%)

 Click here to access/download;Table;Table 2.xlsx

Table 2. Quantity of the contigs anchored with Hi-C.

Group	Number of anchored contigs	Sequence Length (bp)
Lachesis Group 1	68	40,738,791
Lachesis Group 2	92	40,039,835
Lachesis Group 3	38	37,159,809
Lachesis Group 4	112	35,552,403
Lachesis Group 5	84	35,291,867
Lachesis Group 6	62	35,706,508
Lachesis Group 7	66	33,002,525
Lachesis Group 8	46	32,947,898
Lachesis Group 9	66	30,804,552
Lachesis Group 10	62	30,699,318
Lachesis Group 11	68	29,306,026
Lachesis Group 12	56	29,390,540
Lachesis Group 13	47	29,816,145
Lachesis Group 14	71	25,601,946
Lachesis Group 15	72	23,228,783
Total (Ratio %)	1,010 (35.61)	489,286,946 (97.04)

Figure 2

Click here to access/download;Figure;Fig.2.tif

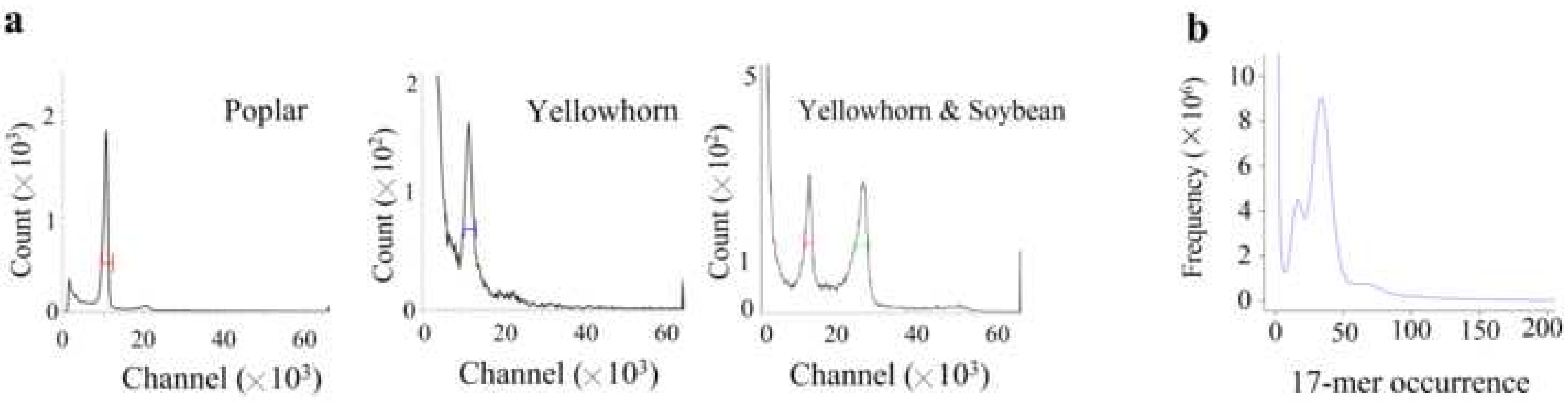

Click here to access/download
Supplementary Material
Tables_AdditionalFiles_1.docx

Click here to access/download
Supplementary Material
Figures_AdditionalFiles_2.docx

Click here to access/download
Supplementary Material
Figures_supporting_data.xlsx

Personal Cover Click here to access/download;Personal Cover;response 5-10.docx

GIGA-D-18-00337R2

Pseudomolecule-level assembly of the Chinese oil tree yellowhorn (*Xanthoceras sorbifolium*) genome

Quanxin Bi; Yang Zhao; Wei Du; Ying Lu; Lang Gui; Zhimin Zheng; Haiyan Yu; Yifan Cui; Zhi Liu; Tianpeng Cui; Deshi Cui; Xiaojuan Liu; Yingchao Li; Siqi Fan; Xiaoyu Hu; Guanghui Fu; Jian Ding; Chengjiang Ruan; Libing Wang, Ph.D.

GigaScience

Dear editor:

Thank you so much for your thorough review and constructive suggestions. We are so sorry to bring you so much trouble because of our careless. We also thanks the reviewer's professional suggestions and we have responded to the reviewer's comments point-to-point and made corresponding revisions to the manuscript entitled "Pseudomolecule-level assembly of the Chinese oil tree yellowhorn (*Xanthoceras sorbifolium*) genome".

We look forward to a favorable decision from you.

Thanks and Best wishes

Yours sincerely

Libing Wang

Detailed response to the reviewer

The authors would like to thank the reviewers for their kind and constructive comments and fell that they have strengthened the manuscript. Responses to the reviewer's comment are listed below. All the responses have been highlighted in blue color.

Reviewer #2: The Authors appear to have gone to a good deal of effort to address the points I raised previously and I appreciate the work that they have put in. Unfortunately, I still have a concern regarding the k-mer analysis and evidence for heterozygosity in the sequenced individual, which I do not feel has been adequately addressed in the Authors' response.

On page 5, lines 21-25, the Authors say that they use the same formula as Varshney et al., (2012; doi:10.1038/nbt.2022) to calculate estimated genome size from the k-mers: Genome size = k-mer number / peak depth. The Authors determined the peak depth to be 34x. However, an important point is that the k-mer frequency plot in Varshney et al., (their Supplementary Figure 2) has a single clear peak, which they use to define the overall depth. This is not unexpected because Varshney et al., (2012) sequenced an inbred line, and the single clear peak in their k-mer plot reflects the fact that the majority of the genome is homozygous (they estimated 0.067% heterozygosity on the basis of SNP calling against the genome assembly). However, in outbreeding diploid individuals we normally expect to see two peaks - one reflecting k-mers that overlap homozygous positions and another from k-mers that overlap heterozygous positions. The multiplicity (count) of the heterozygous peak should be c. half that of the homozygous peak, as shown in Fig 2 of Kajitani et al (2014; doi/10.1101/gr.170720.113). In the k-mer plot in Fig. 2b of Bi et al., there are two clear peaks, one at c. 35x and the other closer to 70x; this means that a

proportion of the 17-mers have a significantly higher depth than that used when calculating the genome size (i.e. 34x), which could lead to an overestimation of the size of the genome based on this method if not accounted for. However, a more important point (given that the Authors also generated genome size estimates by flow cytometry, so do not have to rely on the k-mer based estimate) is the evidence for heterozygosity this indicates. I suggest that the rightmost of the peaks in Fig. 2b (i.e. at c. 70x) is the homogzygous peak and the other is the heterozygous peak, which would indicate a high level of heterozygosity within the sequenced yellowhorn individual.

Response: Thanks for your comments. As you mentioned and our *K*-mer analysis (see below), the heterozygosity of yellowhorn is at a high level, really. According the reviewer #2's suggestion, we did not rely on the estimated genome size by K-mer analysis and used the results of flow cytometry as yellowhorn genome size in revision 2 (See the" Estimation of genome size through a flow cytometry analysis" section in revision 2).

Sorry for our neglect, we had used a quite misleading figure as shown in Figure.2b in the previous manuscript, which affected reviewer's judgments. To avoid misleading, we have changed the Fig.2b (Fig.5.1a, see the screenshot below) in previous manuscript by Fig.5.1b (see the screenshot below) in revision 2.

In *K*-mer analysis, we counted the 17-mer occurrency (17-mer depth) and the frequency of those 17-mers at a given sequencing depth and drew distribution curves of K-mer frequency (Fig.5.1a and Fig.5.1b in genome survey test report, see the screenshot below). The Fig.5.1a (the screenshot below), which is used as Fig.2b in our previous manuscript, the X-axis represents the frequency of 17-mer occurrence (17-mer sequencing depth) and Y-axis represents the product of frequency of 17-mer occurrence and the species of 17-mers in this frequency (total number of

17-mer individuals in this frequency). The repeat peak (66×) was more obvious than the heterozygous peak (17×), which misleading the readers.

However, the Fig.5.1b, the X-axis represents frequency of 17-mer occurrence and the Y-axis represents the frequency of those 17-mers' species at a given sequencing depth. After checking, in order to clearly and intuitively show distribution of 17-mer frequency, the distribution curve of 17-mer frequency should be Fig.5.1b (Li et al, 2010; Zhang et al, 2012; Gao et al, 2018; Zhao et al, 2018; Gao et al, 2018; Wan et al, 2018). Therefore, we changed the Fig.5.1a (Fig.2b in the previous manuscript) by Fig.5.1b in revision 2. The figures supporting data is shown in Figures_supporting_data.xlxs of supplymentary materials. There were two clear peaks (17×and 34×) in Fig.5.1b, indicating high heterozygosity. The tiny peak (66×) observed in Fig.5.1b was caused by the repetitive sequences.

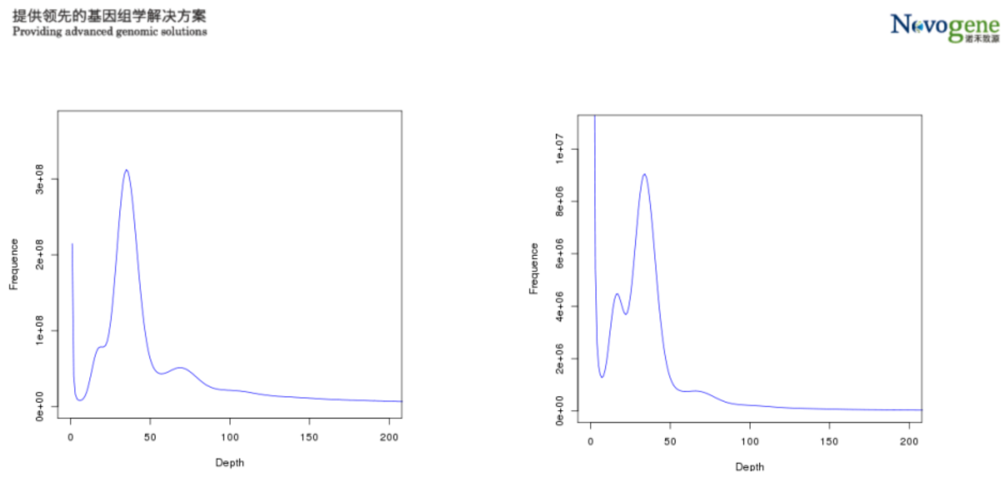

图 5.1a Kmer=17 Depth 和 K-mer 个数频率分布图

图 5.1b Kmer=17 Depth 和 K-mer 种类数频率分布图

Fig.5.1a 17-mer Depth and frequency distribution of number of *K*-mer

Fig.5.1b 17-mer Depth and frequency distribution of *K*-mer species

References

Gao F, Wang X, Li XM et al. Long-read sequencing and de novo genome assembly of *Ammopiptanthus nanus*, a desert shrub. Gigascience. 2018, 7: giy074.

Gao Y, Wang HB, Liu C et al. De novo genome assembly of the red silk cotton tree (*Bombax ceiba*). Gigascience. 2018,7:1–7.

Guan R, Zhao YP, Zhang H, Fan GY et al. Draft genome of the living fossil *Ginkgo biloba*. GigaScience , 2016, 5:49-62.
Li RQ, Fan W, Tian G et al.The sequence and de novo assembly of the giant panda genome. Nature, 2010, 463: 311–317.
Lin Y, Min J, Lai R et al. Genome-wide sequencing of longan (*Dimocarpus longan* Lour.) provides insights into molecular basis of its polyphenol-rich characteristics. Gigascience 2017; 6: 1-14.
Wan T, Liu ZM, Li LF et al. A genome for gnetophytes and early evolution of seed plants. *Nature Plants* 2018, 4: 82–89.
Zhang GF, Fang XD, Guo XM et al. The oyster genome reveals stress adaptation and complexity of shell formation. Nature, 2012, 490:49-54.
Zhao HS, Wang SB, Wang JL et al. The chromosome-level genome assemblies of two rattans (*Calamus simplicifolius* and *Daemonorops jenkinsiana*). Gigascience. 2018. 7 : giy097.

The reason I believe this is as follows - the Authors report that they have 34.40 Gb of cleaned Illumina HiSeq reads (first line on page 5) and that the estimated 1C genome size for yellowhorn is c. 535Mb (average of the two estimates reported on page 4) based on flow cytometry, so the expected whole genome coverage from the Illumina reads is c. x64. Therefore, in a k-mer plot generated from the Illumina reads we would expect to see the homozygous peak at around c. 65x, which is approximately the position of the rightmost peak in Fig. 2b. Consequently, the much higher leftmost peak reflects the heterozygous content of the genome.

Response: Thanks for your comments. As mentioned above, there were two clear peaks (17× and 34×) in Fig.5.1b (Fig.2b in revision 2). Homozygous peak depth was at 34×, and the peak of 17× was heterozygous peak. The tiny peak (66×) observed in Fig.5.1b was caused by the repetitive sequences. The reasons are as follows.

As mentioned in our last response, before conducting whole genome sequencing, a genome survey was performed to estimate the genomic characteristics of yellowhorn. A total of 34.51 Gb raw sequencing data were generated by Illumina platform. After removed the adapter reads, low quality reads, 34.4 G clean data were obtained. Next, data filtering was performed again to remove

the duplication (6.08 G) and contaminated reads (2.92 G). In addition, the end base of sequencing reads (4.23G) also need to be removed because the quality of sequencing base at the end of sequence was low. After filtering the low-quality, duplicated and contaminated reads, 21.17 Gb high-quality useful sequencing data were utilized to generate a *K*-mer (K = 17) depth distribution curve, and generated 18458632032 *17*-mer. The previous description is not clear. Now we have added related information in a revised version 2.

According to the formula mentioned by Liu and Michael S. Waterman's group (Liu et al, 2013; Li et al, 2003), one read with length L generates L—*K*+1 *K*-mers, thus

$$C_{base} = C_{k\text{-}mer} \times L / (L - K + 1)$$

$$G = N_{base}/C_{base} = N_{k\text{-}mer}/C_{k\text{-}mer}$$

Let n_{base}, $n_{k\text{-}mer}$ be the total number of bases and *K*-mers from reads data, and C_{base}, $C_{k\text{-}mer}$ be the expected coverage depth for bases and *K*-mer. After filtering the end base of sequencing reads, the L is 125 bp. Through flow cytometry analysis, the yellowhorn genome size was estimated to be approximately 525.94 Mb to 540.93 Mb, thus the C_{base} was ~ 39× and the $C_{k\text{-}mer}$ was ~ 34×.The homogzygous peak depth was at 34×. The sequencing depth of heterozygous sequence is approximately half that of homozygous sequence, and the repeat sequences can cause depth doubling (Zhang et al. 2012; Kajitani et al, 2014). The peak of 17× was heterozygous peak and the tiny peak (66×) observed in Fig.5.1b was caused by repeat sequences. In addition, *K*-mer analysis was used to estimate the heterozygosity according to the methods mentioned by Liu et al (Liu et al, 2013; Zhao et al, 2018; Dong et al, 2108). The heterozygosity was estimated at 0.75%, which was a high level.

References

Zhang GF, Fang XD, Guo XM et al. The oyster genome reveals stress adaptation and complexity of shell formation. Nature, 2012, 490:49-54.

Liu BH, Shi YJ, Yuan JY et al. Estimation of genomic characteristics by analyzing kmer frequency in de novo genome projects. Quant Biol. 2013; doi:10.1016/S0925-4005(96)02015-1.

Li, X. and Waterman, M.S. Estimating the repeat structure and length of DNA sequences using L-tuples, Genome res. 2003, 13, 1916-1922.

Kajitani R, Toshimoto K, Noguchi H et al. Efficient de novo assembly of highly heterozygous genomes from whole-genome shotgun short reads. Genome Res. 2014, 24: 1384-1395

Zhao HS, Wang SB, Wang JL et al. The chromosome-level genome assemblies of two rattans (*Calamus simplicifolius* and *Daemonorops jenkinsiana*). Gigascience. 2018. 7 : giy097.

Dong AX, Xin HB, Li ZJ et al. High quality assembly of the reference genome for scarlet sage, *Salvia splendens,* an economically important ornamental plant. Gigascience. 2018, 7: giy068.

As I mentioned in my previous review, the fact that the initial genome assembly (598.65Mb) is significantly larger than expected based on the genome size estimated by flow cytometery is further evidence for heterozygosity. The Authors did not specifically address this point in their response. Furthermore, it is indicated that c. 95Mb of the assembly is removed during filtering for heterozygous sequences, again suggesting a relatively high level of heterozygosity in the sequenced individual.

Response:

We also found that the heterozygosity (0.75%) of yellowhorn is a high level, using *K*-mer analysis. In addition, the distribution curve of 17-mer frequency shown that the heterozygosity was about ~0.5% to ~1.0% according to Fig.2b in Kajitani et al (2014), which was consistant with the result we calculated. This result was not mentioned in our previous manuscript and we added it in revision 2.

In addition, we performed *K*-mer analysis using Illumina data again and performed genome survey on other 3 yellowhorn cultivars (Zhongshi 2, Zhongshi 5 and Zhongshi 9) to identify the heterozygosity in yellowhorn genome. And 35.71 Gb, 33.18 Gb and 39.55Gb clean data were

obtained from Illumina platform. After filtering the low-quality, duplicated and contaminated reads, 24.62 Gb, 22.89 Gb and 29.13 Gb high-quality useful sequencing data were utilized to *K*-mer analysis. The results (Response Table 1, Response Fig.2) indicated that the heterozygosity of three yellowhorn cultivars were 0.66 %, 0.77% and 0.89%, respectively, which agreed with the heterozygosity (0.75%) of sequencing individual.

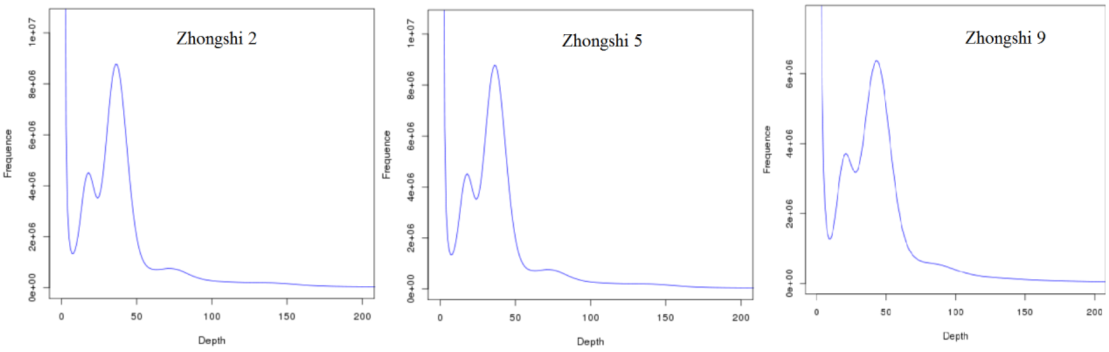

Response Fig.2 Distribution of 17-mer of three yellowhorn cultivars.

Preliminary assembly was performed by Falcon v0.7. Falcon begins by error-correcting PacBio raw sequence data through long-read to long-read sequence alignments and subsequently constructs a string graph of the overlapping reads, which contain sets of “haplotype-fused” contigs and variant sequence (Chin et al, 2016). For the integrity of assembly, the assembly algorithm in Falcon would specifically take into account heterozygous sequence and amount of heterozygous sequences were assembled (Chin et al, 2016). As mentioned above, the estimated heterozygosity of sequencing individual was 0.75%, with a high level of heterozygosity. The initial assembly contained amount of heterozygous sequences, which would be filtered by downstream analysis.

References

Liu BH, Shi YJ, Yuan JY et al. Estimation of genomic characteristics by analyzing kmer frequency in de novo genome projects. Quant Biol. 2013; doi:10.1016/S0925-4005(96)02015-1.

Chin CS, Peluso P, Sedlazeck FJ et al. Phased Diploid Genome Assembly with Single Molecule Real-Time Sequencing. doi: 10.1101/056887v1.

Kajitani R, Toshimoto K, Noguchi H et al. Efficient de novo assembly of highly heterozygous genomes from whole-genome shotgun short reads. Genome Res. 2014, 24: 1384-1395

In the revised version of the manuscript, the Authors estimate % heterozygosity by calling SNPs from reads mapped to the genome assembly, which gives an estimate of 0.30%. This is a much lower level of heterozygosity than would be expected if my assertion regarding the peaks in the k-mer frequency plot is correct. However, there is a lack of detail regarding the parameters used for SNP calling, such as thresholds for mapping quality and any filtering that was applied to the set of raw SNPs.

Nevertheless, if we assume that this estimate of heterozygosity is more or less correct, the discrepancy between the expected genome coverage from the Illumina reads and the coverage suggested from the most prominent peak in the k-mer plot based on these reads still needs to be explained. Moreover, it remains the case that the size of the initial genome assembly was significantly larger than the expected genome size based on flow cytometry, and that a substantial amount of sequence was removed during filtering with Purge Haplotigs, which would not be expected if the level of heterozygosity was really only 0.30%.

Response: As reviewer's statement and our above analysis, we also think the heterozygosity estimated by calling SNPs (0.3%) is a much lower level.

When performed SNP calling, Illumina reads were aligned to the assembly and variable sites were identified by Bowtie 2.2.5 and GATK (V2.8.1). This protocol could accurately detect the SNP sites in genome assembly and had been widely used for calculating the heterozygosity of genome (Li et al, 2010; Peng et al, 2013). It was the reason that we calculated the heterozygosity by calling SNPs in our previous manuscript. The parameters of HaplotypeCaller module in GATK to filter unreliable SNPs were as follows: QD < 2.0 jj MQ < 40.0 jj FS > 60.0 jj QUAL <30.0 jj MQrankSum < -12.5 jj ReadPosRankSum < -8.0 -clusterSize 2 -clusterWindowSize 5.

Thank you so much for your comments. We also found the heterozygosity calculated by SNP calling is a much lower level. And there are possible reasons for the low heterozygosity estimated by SNP calling.

First, the average PE read depth was about 40-fold coverage in our study. NGS data could suffer from high error rates due to multiple factors, including base-calling and alignment errors, thus the more deeply sequencing depth was required to ensure the accuracy of SNP calling results (Nielsen et al, 2013). In addition, amount of SNP sites that may be filtered if the sequencing depth of SNP position was lower than the threshold, whereas these sites would be counted as heterozygous sites in *K*-mer analysis. Furthermore, the yellowhorn genome had high repeat rates (reached 68.67%), which had a great influence on the accuracy of SNP calling.

And as reviewer's mentioned, the initial assembly was ~598 Mb but the final assembly was ~504 Mb, indicated the ~95 Mb of the assembly is removed as heterozygous sequences. However the estimated genome size was ~540M, thus there were ~35 Mb genome sequences were filtered as heterozygous sequences for their high heterozygosity, which had a great influence on SNP calling.

In a word, the SNP result was less than the truth heterozygosity of yellowhorn genome. The heterozygosity of yellowhorn genome was about 0.75%, estimated by *K*-mer analysis instead of SNP calling (see the "Illumina short-read sequencing and heterozygosity analysis" section in revision 2).

References

Nielsen R, Paul J S, Albrechtsen A et al. Genotype and SNP calling from next-generation sequencing data. Nat Rev. 2013, 12,443-451.

Li RQ, Fan W, Tian G et al.The sequence and de novo assembly of the giant panda genome. Nature. 2010, 463:

311–317.
Peng ZH, Lu Y, Li LB et al. The draft genome of the fast growing non-timber forest species Moso Bamboo (*Phyllostachys heterocycla*). Nat genet. 2013, 45: 456-463.

In addition to the issue discussed above, the English language requires some further improvement. There are a number of small improvements that could be made to increase the clarity and readability of the text. Issues include things like the use of a plural when the singular form should be used (e.g. "individuals" when "individual" is meant) or vice versa (e.g. use of "assemblies" when "assembly" is meant), spelling errors (e.g. "pepline" instead of "pipeline", "eudicot" misspelt as "endicot" in several places) and missing words or incorrect word choice (e.g. "produced via clone" rather than "produced via cloning', "from natural population" rather than "from a natural population"). I recommend that the manuscript be further reviewed for English language prior to publication.

Response: Thanks for your comments about our English writing. We have corrected all errors you pointed out in our revised version 2. The revisions in the manuscript have been highlighted in red color.

According to editor and reviewer’s suggestion, we also submitted our Revision 2 manuscript to a language editing company (Edanz Group) to improve our English. And we thank Robbie Lewis, MSc, from Liwen Bianji, Edanz Group China (www.liwenbianji.cn/ac), for editing this manuscript.
