# Supplementary material for: Pseudomolecule-level assembly of the Chinese oil tree yellowhorn (Xanthoceras sorbifolium) genome
Source: Gigascience. 2019 Jun 26;8(6):giz070. doi: 10.1093/gigascience/giz070 (PMC6593361; doi:10.1093/gigascience/giz070)
Supplement: giz070_Supplemental_Files [file giz070_supplemental_files.zip › Figures_AdditionalFiles_2.docx]

Additional file 2

***Pseudomolecule-level assembly of the Chinese oil tree yellowhorn (Xanthoceras sorbifolium) genome***

Figure S1: Length distribution of the three types of PacBio reads produced.

Figure S2: Interaction frequency distribution of Hi-C links among chromosomes.

Figure S3. Distribution of insertion ages of *Copia*-type and *Gypsy*-type LTR-retrotransposons.

Figure S4: Function classification of protein-coding genes against the GO term database.

Figure S5: KOG function classification of protein-coding genes.

**Figure S1**. Length distribution of the three types of PacBio reads produced.


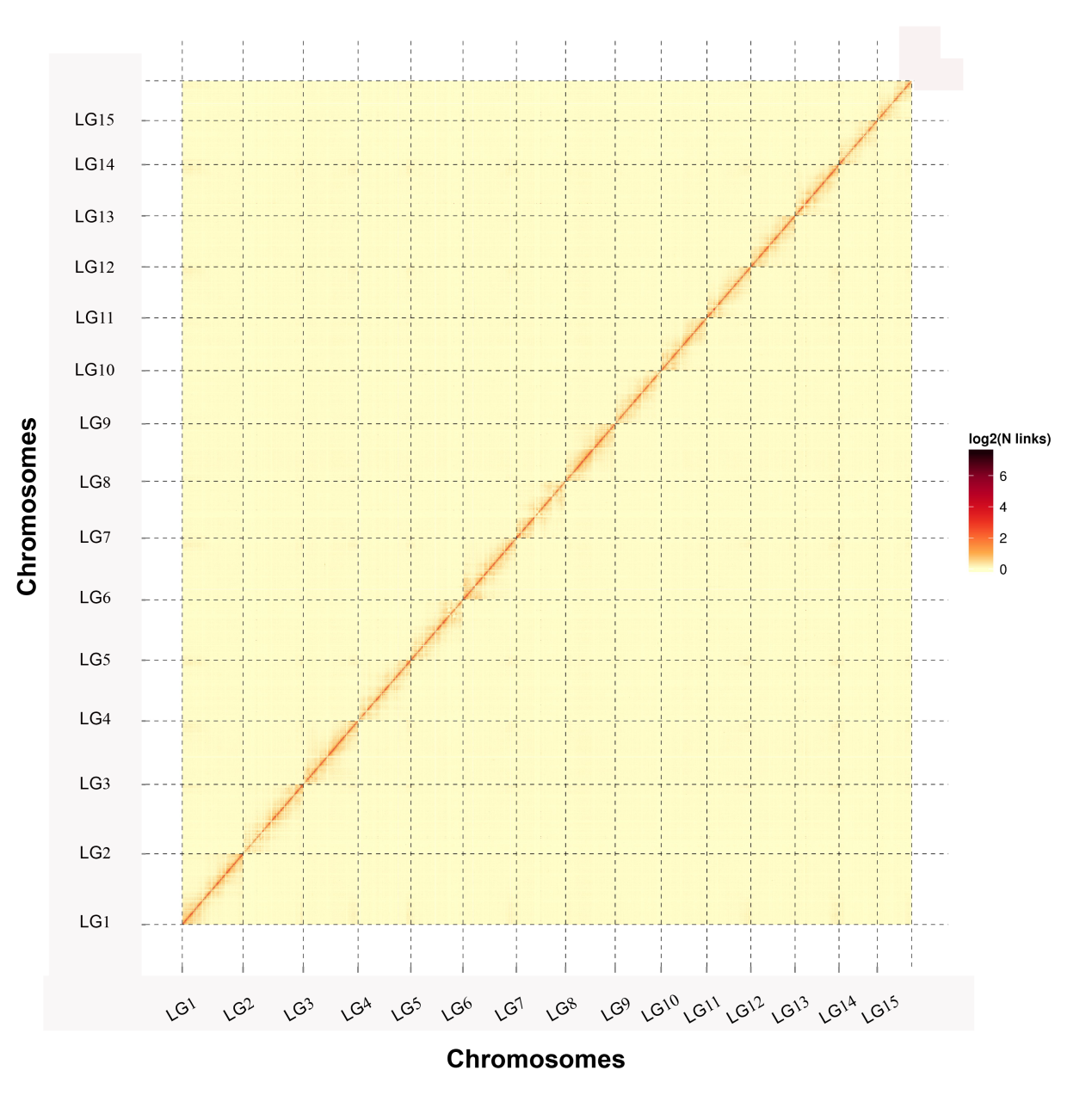


**Figure S2**. Interaction frequency distribution of Hi-C links among chromosomes.


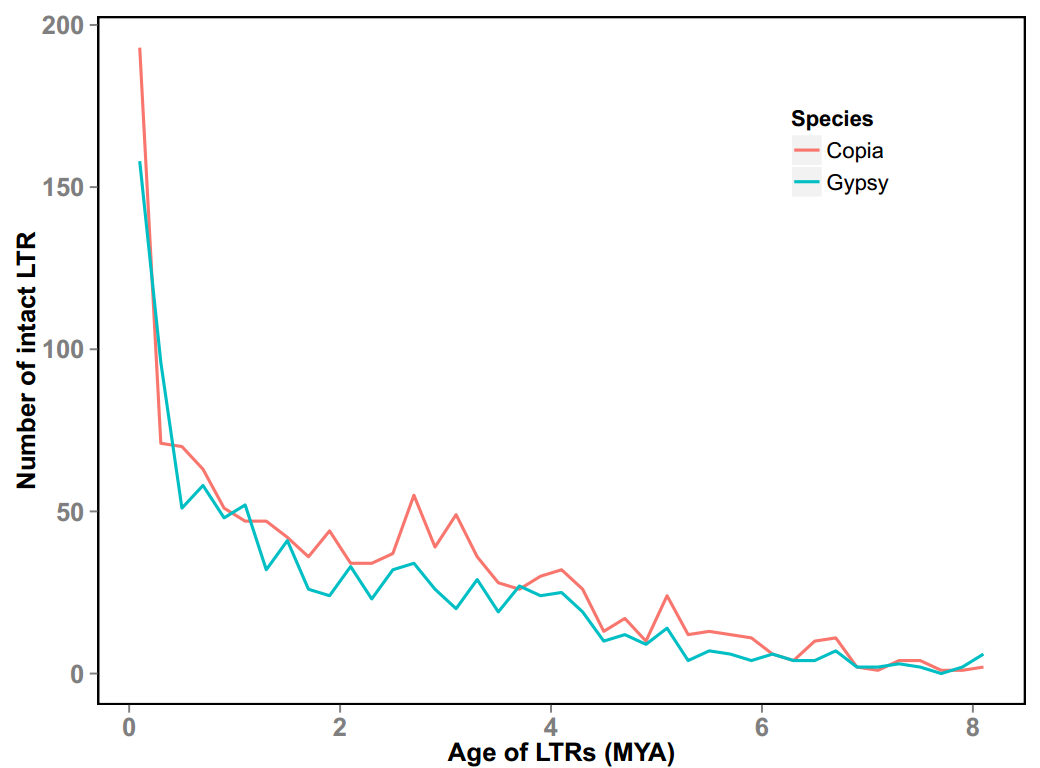


**Figure S3.** Distribution of insertion ages of *Copia*-type and *Gypsy*-type LTR-retrotransposons.


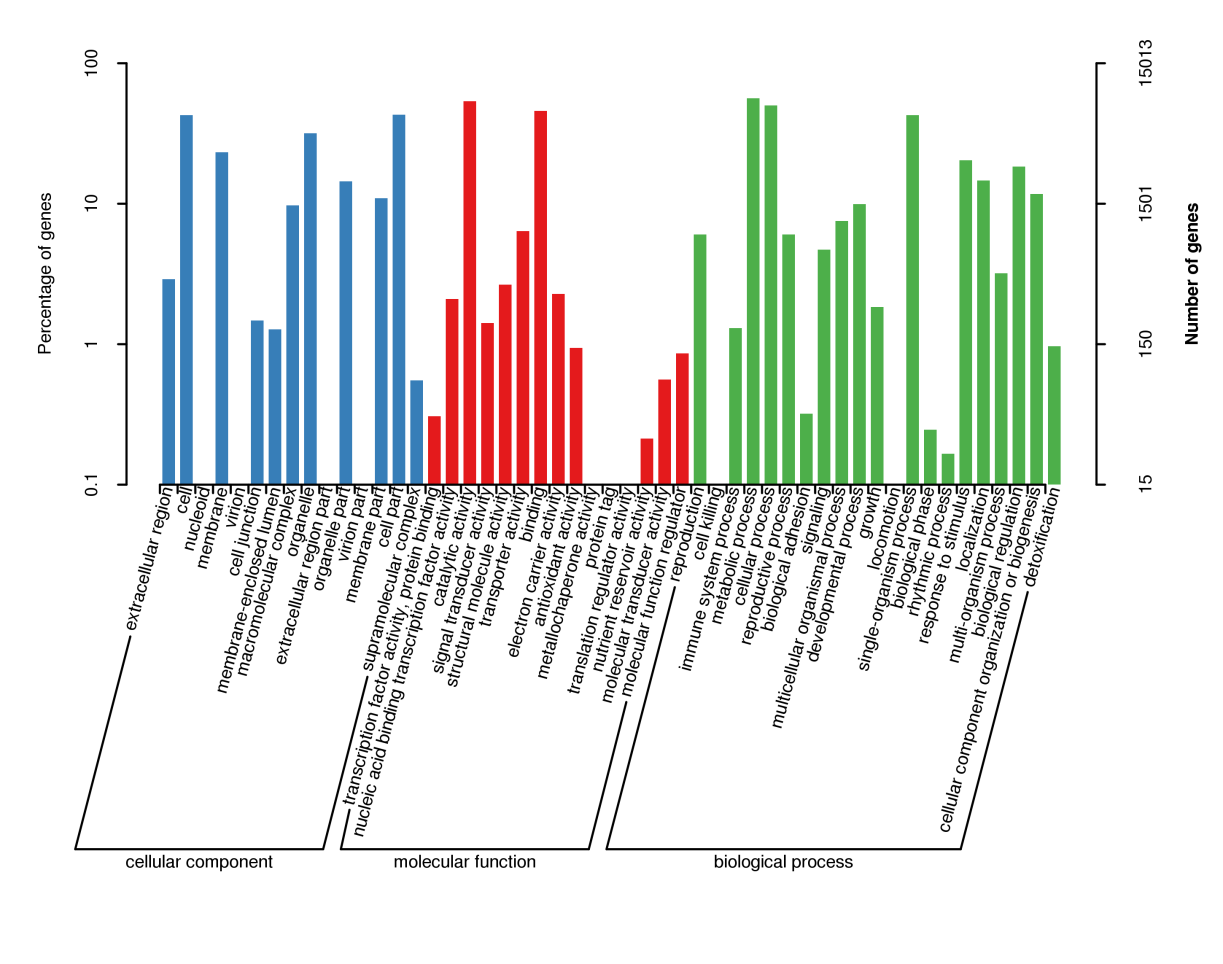


**Figure S4**. Function classification of protein-coding genes against the GO term database.


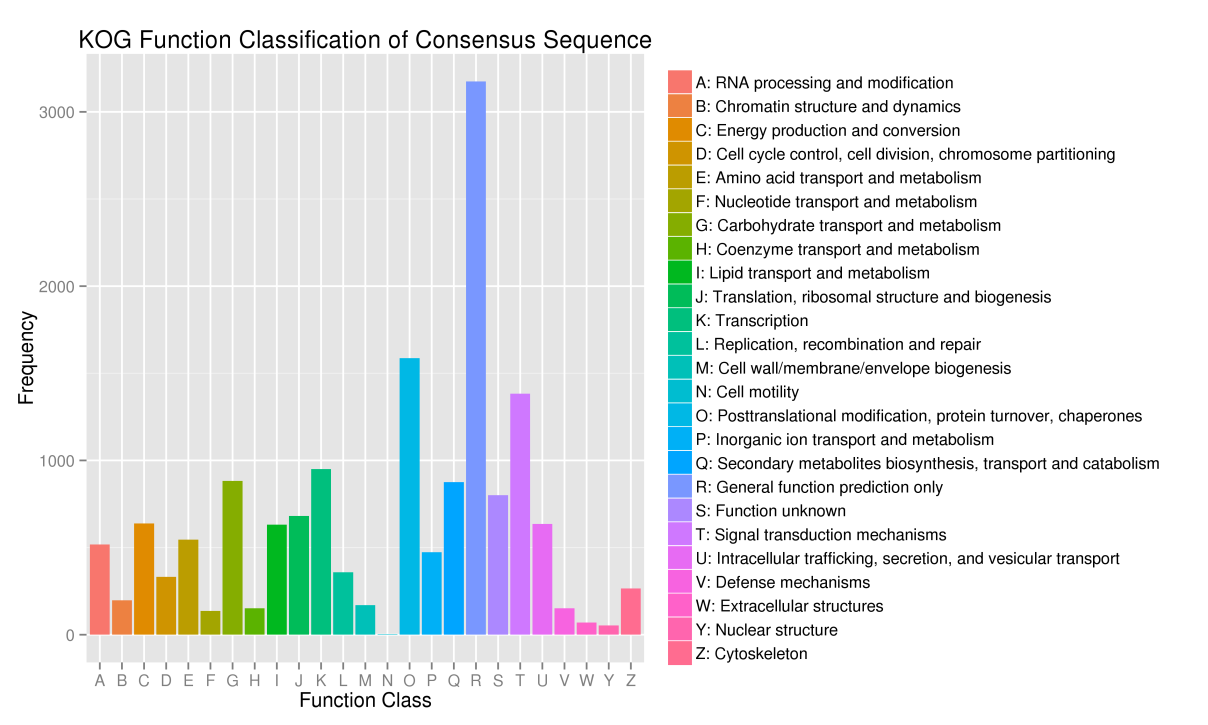


**Figure S5**. KOG function classification of protein-coding genes.
