# Supplementary material for: Pseudomolecule-level assembly of the Chinese oil tree yellowhorn (Xanthoceras sorbifolium) genome
Source: Gigascience. 2019 Jun 26;8(6):giz070. doi: 10.1093/gigascience/giz070 (PMC6593361; doi:10.1093/gigascience/giz070)
Supplement: giz070_Supplemental_Files [file giz070_supplemental_files.zip › Tables_AdditionalFiles_1.docx]

Additional file 1

***Pseudomolecule-level assembly of the Chinese oil tree yellowhorn (Xanthoceras sorbifolium) genome***

Table S1: PacBio data statistics.

Table S2: Genome quality assessed by the BUSCO test.

Table S3: Repetitive sequence content.

Table S4: Prediction of protein-coding genes.

Table S5: Function annotation of protein-coding genes.

Table S6: Data used in orthoMCL analysis.

Table S7: Annotation and locus information of 169 yellowhorn-specific gene families.

**Table S1.** PacBio data statistics.

| Read type | Read base (bp) | Read Number | Maximum read length (bp) | Average read length (bp) | Read N50 length (bp) |
| --- | --- | --- | --- | --- | --- |
| Polymerase | 66,514,387,859 | 4,554,059 | 101,573 | 14,605 | 24,742 |
| Insertsize | 54,525,277,533 | 4,554,059 | 85,523 | 11,972 | 18,936 |
| Subreads | 66,442,751,726 | 6,105,692 | 85,523 | 10,882 | 17,598 |

**Table S2.** Genome quality assessed by the BUSCO test.

**Summary:** C, 94.7% [S, 89.0%; D, 5.7%]; F, 1.7%; M, 3.6%; n, 1440

Complete BUSCOs (C) 1,364

Complete and single-copy BUSCOs (S) 1,282

Complete and duplicated BUSCOs (D) 82

Fragmented BUSCOs (F) 24

Missing BUSCOs (M) 52

Total BUSCO groups searched 1,440

Notes:

1) BUSCO version: 3.0.2

2) The lineage dataset: embryophyta_odb9 (Creation date: 2016-02-13, number of species: 30, number of BUSCOs: 1440)

3) BUSCO was run at https://gvolante.riken.jp/analysis.html.

**Table S3.** Repetitive sequence content..

| **Type** | **Quantity** | **Length (bp)** | **% of genome** |
| --- | --- | --- | --- |
| ClassI/DIRS | 33,900 | 34,492,801 | 6.84 |
| ClassI/LINE | 103,521 | 48,918,448 | 9.70 |
| ClassI/LTR | 3,746 | 2,855,215 | 0.57 |
| ClassI/LTR/Copia | 167,425 | 98,677,964 | 19.56 |
| ClassI/LTR/Gypsy | 112,090 | 88,241,857 | 17.49 |
| ClassI/PLE\|LARD | 7,843 | 6,927,083 | 1.37 |
| ClassI/SINE | 5,606 | 1,241,802 | 0.25 |
| ClassI/TRIM | 1,951 | 880,546 | 0.17 |
| ClassI/Unknown | 18,293 | 4,668,853 | 0.93 |
| ClassII/Crypton | 548 | 111,160 | 0.02 |
| ClassII/Helitron | 17,238 | 5,819,506 | 1.15 |
| ClassII/MITE | 2,252 | 690,102 | 0.14 |
| ClassII/Maverick | 2,650 | 1,479,420 | 0.29 |
| ClassII/TIR | 84,179 | 40,827,673 | 8.09 |
| ClassII/Unknown | 8,399 | 2,874,579 | 0.57 |
| PotentialHostGene | 90,395 | 25,403,790 | 5.04 |
| SSR | 3,984 | 1,157,168 | 0.23 |
| Unknown | 266,818 | 94,657,787 | 18.77 |
| Total with overlap: | 930,838 | 459,925,754 | 91.18 |
| Total without overlap: | 930,838 | 346,368,166 | 68.67 |

**Table S4.** Prediction of protein-coding genes

| **Method** | **Software** | **Species** | **Gene number** |
| --- | --- | --- | --- |
| *Ab initio* | Genscan | - | 20,501 |
|  | Augustus | - | 30,925 |
|  | GlimmerHMM | - | 27,101 |
|  | GeneID | - | 31,517 |
|  | SNAP | - | 34,890 |
| Homology-based | GeMoMa | *Arabidopsis thaliana* | 23,088 |
|  |  | *Vitis vinifera* | 22,672 |
|  |  | *Dimocarpus longan* | 31,407 |
| RNAseq | PASA | - | 30,217 |
| Integration | EVM | - | 24,672 |

**Table S5**. Function annotation of protein-coding genes.

| **database** | **Annotated number** | **Percentage (%)** |
| --- | --- | --- |
| GO | 15,013 | 60.85% |
| KEGG | 8,599 | 34.85% |
| KOG | 13,836 | 56.07% |
| Pfam | 20,795 | 84.27% |
| Swissprot | 18,063 | 73.20% |
| TrEMBL | 24,315 | 98.53% |
| eggNOG_Annotation | 461 | 1.87% |
| NR | 24,316 | 98.53% |
| NT | 24,008 | 97.27% |
| **Genes with at least one functional domain** | **24,429** | **99.02%** |

**Table S6**.Data used in orthoMCL analysis

| **species** | **Assembly/Annotation version** | **File name/Dataset name** | **Source** |
| --- | --- | --- | --- |
| Citrus clementina | V1.0 | GCF_000493195.1_Citrus_clementina_v1.0_protein.faa | Citrus genome database |
| Dimocarps. Longan | V1.0 | longan.pep | GIGADB DATASETS |
| Brassica rapa | V3.0 | Brapa_genome_v3.0_pep.fasta.gz | Brassica Database |
| Arabidopsis thaliana | TAIR10 | TAIR10_GFF3_genes.gff.representative.pep. | Brassica Database |
| Theobroma cacao | v1.1/V2 | GCF_000208745.1_Criollo_cocoa_genome_V2_protein | Cacao Genome Database |
| Gossypium raimondii | V1.0 | G.raimondii_BGI-CGP_v1.0_protein.fasta | CottonGen |
| Quercus robur | V2_2N/ | Qrob_PM1N_CDS_aa_20161004.fa | Oak Genome Sequencing |
| Vitis vinifera | GenomeScope.12X | Vitis_vinifera.IGGP_12x.pep.all.fa | Phytozome V12.1 |
| Cucumis sativus | V3.0 | Cucumber_V3_chr_201810.pep.fa.gz | CuGenDB |
| Malus × domestica | GDDH13 v1.1 | GDDH13_1-1_prot.fasta.bz2 | Genome Database for rosaceae |

| **Table S7** Annotation and locus information of 169 yellowhorn-specific gene families. | | |
| --- | --- | --- |
| Gene Family_number | Pfam_ann | Descriptioin |
| GF_22893 | PF00107.21 | Zinc-binding dehydrogenase |
| GF_16968 | PF13522.1 | Glutamine amidotransferase domain |
| GF_20338 | PF00338.17 | Ribosomal protein S10p/S20e |
| GF_20403 | PF00892.15 | EamA-like transporter family |
| GF_17473 | PF00010.21 | Helix-loop-helix DNA-binding domain |
| GF_20393 | PF03080.10 | Domain of unknown function (DUF239) |
| GF_22897 | PF00300.17 | Histidine phosphatase superfamily (branch 1) |
| GF_22896 | PF05651.8 | Putative sugar diacid recognition |
| GF_20351 | PF02321.13 | Outer membrane efflux protein |
| GF_20370 | PF00657.17 | GDSL-like Lipase/Acylhydrolase |
| GF_20385 | PF00413.19 | Matrixin |
| GF_20365 | PF01176.14 | Translation initiation factor 1A / IF-1 |
| GF_20416 | PF00355.21 | Rieske [2Fe-2S] domain |
| GF_20389 | PF14541.1 | Xylanase inhibitor C-terminal |
| GF_20452 | PF00075.19 | RNase H |
| GF_20404 | PF13857.1 | Ankyrin repeats (many copies) |
| GF_17438 | PF04970.8 | Lecithin retinol acyltransferase |
| GF_17423 | PF13962.1 | Domain of unknown function |
| GF_20399 | PF13360.1 | PQQ-like domain |
| GF_20461 | PF05495.7 | CHY zinc finger |
| GF_20398 | PF02365.10 | No apical meristem (NAM) protein |
| GF_16131 | PF00628.24 | PHD-finger |
| GF_20401 | PF00665.21 | Integrase core domain |
| GF_20373 | PF00069.20 | Protein kinase domain |
| GF_20457 | PF00575.18 | S1 RNA binding domain |
| GF_20415 | PF00069.20 | Protein kinase domain |
| GF_20383 | PF05056.7 | Protein of unknown function (DUF674) |
| GF_20430 | PF03087.9 | Arabidopsis protein of unknown function |
| GF_20419 | PF03140.10 | Plant protein of unknown function |
| GF_20361 | PF06376.7 | Protein of unknown function (DUF1070) |
| GF_20349 | PF00319.13 | SRF-type transcription factor (DNA-binding and dimerisation domain) |
| GF_20356 | PF00234.17 | Protease inhibitor/seed storage/LTP family |
| GF_22905 | PF00015.16 | Methyl-accepting chemotaxis protein (MCP) signalling domain |
| GF_20339 | PF04576.10 | Zein-binding |
| GF_20422 | PF07714.12 | Protein tyrosine kinase |
| GF_13523 | PF02458.10 | Transferase family |
| GF_20397 | PF00072.19 | Response regulator receiver domain |
| GF_20337 | PF13600.1 | N-terminal domain of unknown function (DUF4140) |
| GF_20390 | PF00249.26 | Myb-like DNA-binding domain |
| GF_20407 | PF07899.6 | Frigida-like protein |
| GF_20410 | PF13041.1 | PPR repeat family |
| GF_20394 | PF00083.19 | Sugar (and other) transporter |
| GF_20446 | PF06201.8 | PITH domain |
| GF_20458 | PF00428.14 | 60s Acidic ribosomal protein |
| GF_17454 | PF00709.16 | Adenylosuccinate synthetase |
| GF_20343 | PF03885.8 | Protein of unknown function (DUF327) |
| GF_16133 | PF00201.13 | UDP-glucoronosyl and UDP-glucosyl transferase |
| GF_20447 | PF13639.1 | Ring finger domain |
| GF_20336 | PF01734.17 | Patatin-like phospholipase |
| GF_20358 | PF02458.10 | Transferase family |
| GF_19234 | PF03359.8 | Guanylate-kinase-associated protein (GKAP) protein |
| GF_17442 | PF03330.13 | Rare lipoprotein A (RlpA)-like double-psi beta-barrel |
| GF_17464 | PF14543.1 | Xylanase inhibitor N-terminal |
| GF_20406 | PF01734.17 | Patatin-like phospholipase |
| GF_20381 | PF00565.12 | Staphylococcal nuclease homologue |
| GF_15288 | PF00078.22 | Reverse transcriptase (RNA-dependent DNA polymerase) |
| GF_20391 | PF11319.3 | Protein of unknown function (DUF3121) |
| GF_22892 | PF03874.11 | RNA polymerase Rpb4 |
| GF_20374 | PF03936.11 | Terpene synthase family, metal binding domain |
| GF_20346 | PF03106.10 | WRKY DNA -binding domain |
| GF_20429 | PF02668.11 | Taurine catabolism dioxygenase TauD, TfdA family |
| GF_22907 | PF05698.9 | Bacterial trigger factor protein (TF) C-terminus |
| GF_22894 | PF00394.17 | Multicopper oxidase |
| GF_20396 | PF00067.17 | Cytochrome P450 |
| GF_20354 | PF13921.1 | Myb-like DNA-binding domain |
| GF_20388 | PF12776.2 | Myb/SANT-like DNA-binding domain |
| GF_20462 | PF00121.13 | Triosephosphate isomerase |
| GF_20413 | PF13962.1 | Domain of unknown function |
| GF_20387 | PF09748.4 | Transcription factor subunit Med10 of Mediator complex |
| GF_20428 | PF03652.10 | Uncharacterised protein family (UPF0081) |
| GF_20386 | PF03870.10 | RNA polymerase Rpb8 |
| GF_20350 | PF04450.7 | Peptidase of plants and bacteria |
| GF_20463 | PF10123.4 | Mu-like prophage I protein |
| GF_20353 | PF05147.8 | Lanthionine synthetase C-like protein |
| GF_20423 | PF00413.19 | Matrixin |
| GF_20414 | PF07683.9 | Cobalamin synthesis protein cobW C-terminal domain |
| GF_20380 | PF02481.10 | DNA recombination-mediator protein A |
| GF_20371 | PF00931.17 | NB-ARC domain |
| GF_20424 | PF02458.10 | Transferase family |
| GF_20451 | PF01925.14 | Sulfite exporter TauE/SafE |
| GF_20456 | PF03151.11 | Triose-phosphate Transporter family |
| GF_17486 | PF00010.21 | Helix-loop-helix DNA-binding domain |
| GF_17512 | PF03195.9 | Protein of unknown function DUF260 |
| GF_17445 | PF00009.22 | Elongation factor Tu GTP binding domain |
| GF_20411 | PF00467.24 | KOW motif |
| GF_20409 | PF00327.15 | Ribosomal protein L30p/L7e |
| GF_20395 | PF02389.10 | Cornifin (SPRR) family |
| GF_16969 | PF00188.21 | Cysteine-rich secretory protein family |
| GF_14653 | PF14244.1 | gag-polypeptide of LTR copia-type |
| GF_14657 | PF08387.5 | FBD |
| GF_20341 | PF12609.3 | Wound-induced protein |
| GF_22906 | PF03313.10 | Serine dehydratase alpha chain |
| GF_20363 | PF00067.17 | Cytochrome P450 |
| GF_20421 | PF04554.8 | Extensin-like region |
| GF_20357 | PF02453.12 | Reticulon |
| GF_20417 | PF00931.17 | NB-ARC domain |
| GF_20427 | PF00141.18 | Peroxidase |
| GF_20372 | PF13962.1 | Domain of unknown function |
| GF_16970 | PF04851.10 | Type III restriction enzyme, res subunit |
| GF_15294 | PF14227.1 | gag-polypeptide of LTR copia-type |
| GF_20376 | PF00582.21 | Universal stress protein family |
| GF_20379 | PF01852.14 | START domain |
| GF_22895 | PF02309.11 | AUX/IAA family |
| GF_15297 | PF00571.23 | CBS domain |
| GF_20445 | PF03030.11 | Inorganic H+ pyrophosphatase |
| GF_17425 | PF00641.13 | Zn-finger in Ran binding protein and others |
| GF_20342 | PF02458.10 | Transferase family |
| GF_17480 | PF00428.14 | 60s Acidic ribosomal protein |
| GF_20400 | PF10604.4 | Polyketide cyclase / dehydrase and lipid transport |
| GF_20366 | PF00687.16 | Ribosomal protein L1p/L10e family |
| GF_20450 | PF00326.16 | Prolyl oligopeptidase family |
| GF_22891 | PF00728.17 | Glycosyl hydrolase family 20, catalytic domain |
| GF_22908 | PF13414.1 | TPR repeat |
| GF_20360 | PF12796.2 | Ankyrin repeats (3 copies) |
| GF_20375 | PF02682.11 | Allophanate hydrolase subunit 1 |
| GF_22890 | PF00266.14 | Aminotransferase class-V |
| GF_17448 | PF12796.2 | Ankyrin repeats (3 copies) |
| GF_20369 | PF00194.16 | Eukaryotic-type carbonic anhydrase |
| GF_20454 | PF12776.2 | Myb/SANT-like DNA-binding domain |
| GF_20359 | PF03732.12 | Retrotransposon gag protein |
| GF_20431 | PF00069.20 | Protein kinase domain |
| GF_17516 | PF01397.16 | Terpene synthase, N-terminal domain |
| GF_20455 | PF00141.18 | Peroxidase |
| GF_20384 | PF00685.22 | Sulfotransferase domain |
| GF_19240 | PF05608.7 | Protein of unknown function (DUF778) |
| GF_20426 | PF01107.13 | Viral movement protein (MP) |
| GF_20459 | PF03514.9 | GRAS domain family |
| GF_20377 | PF04560.15 | RNA polymerase Rpb2, domain 7 |
| GF_20364 | PF00413.19 | Matrixin |
| GF_22888 | PF00118.19 | TCP-1/cpn60 chaperonin family |
| GF_19231 | PF00183.13 | Hsp90 protein |
| GF_20408 | PF03195.9 | Protein of unknown function DUF260 |
| GF_20345 | PF03168.8 | Late embryogenesis abundant protein |
| GF_20432 | PF00295.12 | Glycosyl hydrolases family 28 |
| GF_22898 | PF02056.11 | Family 4 glycosyl hydrolase |
| GF_17420 | PF01569.16 | PAP2 superfamily |
| GF_17428 | PF00486.23 | Transcriptional regulatory protein, C terminal |
| GF_20402 | PF02458.10 | Transferase family |
| GF_17446 | PF00125.19 | Core histone H2A/H2B/H3/H4 |
| GF_20348 | PF02458.10 | Transferase family |
| GF_16155 | PF00112.18 | Papain family cysteine protease |
| GF_20367 | PF00411.14 | Ribosomal protein S11 |
| GF_16118 | PF00705.13 | Proliferating cell nuclear antigen, N-terminal domain |
| GF_20453 | PF00931.17 | NB-ARC domain |
| GF_20368 | PF13639.1 | Ring finger domain |
| GF_20448 | PF03937.11 | Flavinator of succinate dehydrogenase |
| GF_17507 | PF12697.2 | Alpha/beta hydrolase family |
| GF_15341 | PF03959.8 | Serine hydrolase (FSH1) |
| GF_19233 | PF00015.16 | Methyl-accepting chemotaxis protein (MCP) signalling domain |
| GF_20378 | PF02365.10 | No apical meristem (NAM) protein |
| GF_20344 | PF03140.10 | Plant protein of unknown function |
| GF_20412 | PF00569.12 | Zinc finger, ZZ type |
| GF_22889 | PF03934.8 | Type II secretion system (T2SS), protein K |
| GF_20347 | PF04526.8 | Protein of unknown function (DUF568) |
| GF_19232 | PF01522.16 | Polysaccharide deacetylase |
| GF_20418 | PF04652.11 | Vta1 like |
| GF_20355 | PF00197.13 | Trypsin and protease inhibitor |
| GF_20460 | PF12796.2 | Ankyrin repeats (3 copies) |
| GF_20382 | PF13639.1 | Ring finger domain |
| GF_20340 | PF00141.18 | Peroxidase |
| GF_17413 | PF13041.1 | PPR repeat family |
| GF_20420 | PF08240.7 | Alcohol dehydrogenase GroES-like domain |
| GF_17426 | PF00112.18 | Papain family cysteine protease |
| GF_20449 | PF00106.20 | short chain dehydrogenase |
| GF_22887 | PF13525.1 | Outer membrane lipoprotein |
| GF_17424 | PF05712.8 | MRG |
| GF_20405 | PF03016.10 | Exostosin family |
| GF_20352 | PF07859.8 | alpha/beta hydrolase fold |
| GF_20425 | PF07928.7 | Vps54-like protein |
