# Supplementary material for: Pseudomolecule-level assembly of the Chinese oil tree yellowhorn (Xanthoceras sorbifolium) genome
Source: Gigascience. 2019 Jun 26;8(6):giz070. doi: 10.1093/gigascience/giz070 (PMC6593361; doi:10.1093/gigascience/giz070)
Supplement: giz070_Response_to_Reviewer_Comments_Original_Submission [file giz070_response_to_reviewer_comments_original_submission.pdf]

Author's Response To Reviewer Comments

Close

See the personal cover letter in the last section of the GIGA-D-18-00337_R1

Close
