# Supplementary material for: Pseudomolecule-level assembly of the Chinese oil tree yellowhorn (Xanthoceras sorbifolium) genome
Source: Gigascience. 2019 Jun 26;8(6):giz070. doi: 10.1093/gigascience/giz070 (PMC6593361; doi:10.1093/gigascience/giz070)
Supplement: giz070_Reviewer_1_Report_Original_Submission — John Mackay, Ph.D. -- 10/28/2018 Reviewed [file giz070_reviewer_1_report_original_submission.pdf]

Reviewer Report

Title: Pseudomolecule-level assembly of the Chinese oil tree yellowhorn (Xanthoceras sorbifolium) genome

Version: Original Submission **Date:** 10/28/2018

Reviewer name: John Mackay, Ph.D.

Reviewer Comments to Author:

This manuscript on the genome of Chinese yellowhorn by Bi et al. describes the development and analysis of the assembly of 15 chromosome level pseudo-molecules from a single genotype. The work combines a few different sequencing methods that produced short and long reads and physical map information, along with long RNA-Seq and flow cytometry to independently evaluate the genome size. The combination of methods and bioinformatic steps have apparently produced quite a robust assembly although use of the term "reference genome" may be premature.
The major strength of the paper is the successful integration of the different sequencing methods to produce the assembly that was shown to be complete and of a size that is concordant with the determination by flow cytometry. The evolutionary analysis based on synteny analyses with other angiosperm reference genomes is also well done.
The use of PacBio sequencing was advantageous to obtain long RNA transcript sequences however I found that the determination of the number of genes requires further explanation. The transcriptome assemply contained over 142k sequences but the authors conclude that there are 24,672 genes in the genome; it needs to be explained how the later was obtained and why there is a difference between the two - is it a technical issue or is there a biological explination? Furthermore, the authors report that there are 172 gene families specific to yellowhorn but do not explain what these genes may encode.
The manuscript is generally clearly and concisely written but I noted several typos that need correction (especially in the summary) and recommend the manuscript be carefully edited for language. Here are a few significant ones:
Page 2
Line 5: should read "can withstand very cold and drought conditions"
Line 5: I not sure what is meant by "tertiary legacy"
Line 8: ... understanding...
Line 10: ... genomic era.
Line 13: replace speudomoleculars with speudomolecules
Line 16: The final genome assembly
Line 35: The first sentence needs revising. On what basis is it a "reference genome" it is not discussed in the paper as such.
Line 38: We did not detect any whole-genome...
Line 41: What is meant by "fragment"? Signature?
Page 7
Line 52: replace clustering by clustered

Page 10
Line 52: what is meant by "typical dicot"? Please be more explicit in describing how the species were selected.
Page 11:
Line 44: What is the meaning of "Paralog curse"?

Level of Interest

Please indicate how interesting you found the manuscript: Choose an item.

Quality of Written English

Please indicate the quality of language in the manuscript: Choose an item.

Declaration of Competing Interests

Please complete a declaration of competing interests, considering the following questions:

- Have you in the past five years received reimbursements, fees, funding, or salary from an organisation that may in any way gain or lose financially from the publication of this manuscript, either now or in the future?
- Do you hold any stocks or shares in an organisation that may in any way gain or lose financially from the publication of this manuscript, either now or in the future?
- Do you hold or are you currently applying for any patents relating to the content of the manuscript?
- Have you received reimbursements, fees, funding, or salary from an organization that holds or has applied for patents relating to the content of the manuscript?
- Do you have any other financial competing interests?
- Do you have any non-financial competing interests in relation to this paper?

If you can answer no to all of the above, write 'I declare that I have no competing interests' below. If your reply is yes to any, please give details below.

I declare that I have no competing interests

I agree to the open peer review policy of the journal. I understand that my name will be included on my report to the authors and, if the manuscript is accepted for publication, my named report including any attachments I upload will be posted on the website along with the authors' responses. I agree for my report to be made available under an Open Access Creative Commons CC-BY license (http://creativecommons.org/licenses/by/4.0/). I understand that any comments which I do not wish to be included in my named report can be included as confidential comments to the editors, which will not be published.

I agree to the open peer review policy of the journal

To further support our reviewers, we have joined with Publons, where you can gain additional credit to further highlight your hard work (see: https://publons.com/journal/530/gigascience). On publication of this paper, your review will be automatically added to Publons, you can then choose whether or not to claim your Publons credit. I understand this statement.

Yes Choose an item.
