# Supplementary material for: Pseudomolecule-level assembly of the Chinese oil tree yellowhorn (Xanthoceras sorbifolium) genome
Source: Gigascience. 2019 Jun 26;8(6):giz070. doi: 10.1093/gigascience/giz070 (PMC6593361; doi:10.1093/gigascience/giz070)
Supplement: giz070_Reviewer_2_Report_Original_Submission — Laura Kelly -- 11/11/2018 Reviewed [file giz070_reviewer_2_report_original_submission.pdf]

Reviewer Report

Title: Pseudomolecule-level assembly of the Chinese oil tree yellowhorn (Xanthoceras sorbifolium) genome

Version: Original Submission **Date:** 11/11/2018

Reviewer name: Laura Kelly

Reviewer Comments to Author:

This paper reports the whole genome sequence of Xanthoceras sorbifolium (yellowhorn), a tree species whose uses include oil production. Details of the genes and repeats annotated in the X. sorbifolium are reported, as well as the results of some comparative genomic analyses incorporating data from other already published plant genome sequences. Hi-C data are used to join X. sorbifolium scaffolds into pseudomolecules, and the final assembly approaches chromosomal level.
However, there is insufficient detail provided for several of the analyses and some other aspects of the manuscript require improvements or clarification, details of which I outline below.
Title
I recommend rewording the title to remove reference to "conservation of original chromosomes" because the inferences made regarding the conservation of ancestral characteristics within the X. sorbifolium genome are not well supported by the data presented; see further comments on this point below.
Abstract
Lines 13/14: Change "pseudomoleculars" to "pseudomolecules"; also needs correcting at some other places in the text.
Line 16: Change "The final assembly genome" to "The final genome assembly".
Lines 38-41: Change "We did not detect the whole-genome duplication" to "We did not detect evidence of a whole-genome duplication".
Background
There are various small edits that could be made to improve the clarity of the language used.
Line 32-33: I do not recognise the word "Alzheimerand". I assume this is a typo; should it read "Alzheimer's"?
Sequenced individuals and sample collection
Please clarify if the DNA used for whole genome sequencing comes a single individual or multiple individuals, as suggested by the title of this section; the subsequent section "Illumina short read sequencing" also indicates that multiple individuals were sequenced because it states that DNA was extracted from leaf tissue from "seedlings".
Also, was a voucher specimen of the sequenced individual(s) made? If so, please provide details of the specimen (e.g. collector's number) and state the herbarium or other collection in which it is lodged.
Illumina short read sequencing
Page 4, lines 46-49: "leaf tissues of the same soil-grown seedlings of same plants". I am not clear what these samples are supposed to be the same as. Is it the same seedlings as sampled for the PacBio

libraries? Also, as mentioned above, clarification is needed regarding how many individuals were used for the whole genome sequencing. If multiple individuals were used, the Authors need to state how many individuals were sampled and whether they were grown from seeds from a single mother tree, or seeds taken from multiple trees of the Zhongshi 4 cultivar.

Page 5, lines 1-2: Please clarify what is meant by "HCS 2.0.12.0, RTA 1.17.21.3" in relation to "the standard Illumina pipeline".

Estimation of the genome size by a K-mer analysis

Page 5, line 16: The Authors state that "level of heterozygosity" was estimated. However, as it is not entirely clear whether the genome sequence data represents a single or multiple individuals, I'm not sure if heterozygosity is being estimated, or whether it is in fact polymorphism.

Page 5, line 21-22: Please specify the details of parameter settings used for Canu; if the default settings were used this should be stated. Also, other than the k-mer size, were any other parameters settings modified for Jellyfish?

The reported "heterozygosity" level of 0.36% does not make sense to me given the fact that there is a very prominent peak for heterozygous positions in the k-mer plot in Fig. 2a. In my experience, the type of k-mer frequency profile shown in Fig. 2a suggests a level of heterozygosity far in excess of the value reported; moreover, the fact that it is later reported that the initial genome assembly was significantly larger than the estimated genome size also suggests the actual level of allelic variation within the sequencing data is higher than 0.36%. I have found that with high levels of heterozygosity (e.g. >5%) some k-mer analysis software may fail to properly detect the peaks for heterozygous and homozygous positions. From Fig. 2a, the "hetero" peak seems to be at c. 32x and the "homo" peak at c. 64x. There is also a small shoulder at c. 15x; if this had been erroneously detected as the hetero peak and the c. 32x peak as the homo peak then it would lead to a severe underestimation of the level of heterozygosity in the sequence data, which could explain the mismatch between the value reported and what can be seen in Fig. 2a. The Authors need to provide more details of exactly how the k-mers were used to calculate % heterozygosity, genome size (GS), etc., and also confirm that the hetero and homo peaks were correctly identified during the analysis.

Estimation of genome size through a flow cytometry analysis

I think it would made sense to move the this section so that it is before the sections on sequencing, seeing as depth of genome coverage is reported in the sequencing sections and at that point the genome size of X. sorbifolium had not been given.

It is good to see that the Authors have attempted to estimate GS via flow cytometry (FC) rather than just relying on the estimate from the k-mer analysis. However, there are some issues with the FC analysis. The choice of Populus trichocarpa as a standard for flow cytometry is an unusual one, as this species is not among those that are routinely used for GS estimation by FC in plants (e.g. see Table 1 in Pellicer and Leitch, 2014; DOI 10.1007/978-1-62703-767-9_14). Could the Authors explain why they chose to use P. trichocarpa? Also, no details of the source of the P. trichocarpa material are given (i.e. ex situ collection or original provenance), nor do the Authors specify whether they used the same genotype (Nisqually 1) as used for estimating the reference value for this species. If a different genotype was used, then its GS might differ to that of Nisqually 1, which would in turn create error in the GS estimate for X. sorbifolium. Moreover, the approach used by the Authors does not follow best practice for GS estimation by FC, because the standard and test samples were run separately. Because the exact

position of the 2C peak on the flow histogram for a given sample can differ between runs, in order to obtain an accurate estimate of GS, it is important that the standard and test sample are analysed simultaneously so that the relative position of the peaks can be measured. Can the Authors explain why the P. trichocarpa and X. sorbifolium samples were run separately on the flow cytometer? If the reason was that the peaks were too close together to be easily distinguished when run simultaneously (due to the relatively similar GS of the two species) then the Authors should select an alternative standard (see Pellicer and Leitch, 2014 for details of recommended protocols).

Furthermore, please clarify if the 16 samples from P. trichocarpa were from a single individual, or each from a separate individual, and whether each sample was only run once on the flow cytometer, or multiple times. It would also be useful if other specific details, such as the fluorochrome and isolation buffer used, were provided.

Genome assembly

Please provide details of specific parameter settings used for each piece of software mentioned in this section. In particular, please provide more details of how heterozygous sequences were identified and removed. For example, what criteria were used when deciding which haplotypes to discard and which to keep?

Also, no mention is made of exclusion of organellar sequences; was this performed during the assembly steps, or were these already excluded during the preparation of the sequencing libraries?

Pseudomolecules construction and three-dimensional chromatin conformation analysis

Please give details of any specific parameter settings used for the BWA and HiC-Pro software.

I don't think the actual chromosome number of X. sorbifolium (2n = 30) is mentioned anywhere in the paper; it would make sense to include reference to the chromosome number in this section.

Transcriptome sequencing

Please clarify if the tissues used from RNA extraction were from a single or multiple individuals, and whether the same plant(s) was sampled as for the DNA extractions.

Page 7, lines 51/52: Please specify any parameter settings used with the CD-HIT software.

Evaluation of assemble quality

Correct the title of this section to "Evaluation of assembly quality".

Page 8, lines 16: "including 83.2% single-copy and 11.5% duplicated genes"; these values don't match those in Table S2, please double-check.

Annotation of the repetitive sequences

Please provide details of specific parameter settings used for each piece of software mentioned in this section; currently they are only given for RepeatMasker.

Page 8, line 40/41: Change "of the yellowhorn genome in length" to "of the yellowhorn genome assembly".

Page 8, line 43/44: Please clarify why the results from X. sorbifolium are compared with Citrus sinensis in particular; is this the next most closely related species with a whole genome assembly available after longan? Also, if all the percentages of repeats quoted are expressed in terms of percentage of the assembly size then the comparison may not be very informative if the assemblies for the other species are less complete.

Page 8, line 49: Please double-check all of the percentage values in Table S3, as some appear to be slightly wrong.

Page 8, line 57/58: Please provide details of any software used for the calculation of LTR insertion times; if custom scripts were used, these should be provided.
Page 8, line 60: Please provide details of the source of data used for clementine, longan and grape (either here or in a Table); please specify which versions of genome assemblies and annotations were used.
Page 9, lines 2-5: The Authors make some very broad suggestions about why X. sorbifolium may have a larger number of young LTR insertions compared with the other species, but later they indicate that the differences between species could largely be due to differences in assembly quality, which makes the preceding text somewhat redundant.
Page 9, line 18/19: Correct "LTR-retrotranpsons" to "LTR-retrotranposons".
Page 9, line 21/22: Change "which led to an under-estimated quantity of the" to "which may have led to an under-estimation of the".
Also, Table S3 lists "PotentialHostGene" among the types of repeats, but these are not mentioned in the text. This category of "repeats" makes up c. 5% of the genome assembly; could these be protein-coding genes that have been masked erroneously?
Was any pre-masking of repeat libraries done for captured gene fragments present within repeats, that might cause host genes to be masked as repeats by mistake? Also, were high-copy number genes, such as rDNA genes, accounted for? Or will these also have been masked as repeats? These points need clarifying; if protein-coding genes have been masked as repeats by mistake this would lead to an inflated estimate of the proportion of repetitive DNA and an underestimation of the number of genes within the X. sorbifolium genome.
Prediction of RNA genes
Where not already given, please provide details of specific parameter settings used for each piece of software mentioned in this section. In particular, please give more details of the filtering thresholds used with EVidenceModeler.
Page 9, line 40/41: Please specify which version of the A. thaliana annotation was used. Also "homology-based prediction" should really read "similarity-based prediction".
Page 9, lines 43/47: Please reword "were used as the reference databases aligned the homolog genes in the yellowhorn genome". As currently written, I am not sure what this is supposed to mean.
Page 9, line 57/59: Sentence starting "Finally, the ab initio predicted transcripts", please explain more clearly what is actually been done in this final step. Also, please clarify if any filtering of the GeMoMa gene predictions was done prior to this point.
Page 10, line 13: Change "was used to pseudogene prediction" to "was used to perform pseudogene prediction".
Page 10, line 21/22: Change "The genes were annotated" to "The genes were annotated functionally".
Page 10, line 26/27: Please specify which version of BLAST2GO was used.
Page 10, line 38: 24,429 is not 98.97% of 24,672; please double-check these values.
Identification of gene clusters and duplication
Page 10, line 51/52: Please specify any parameters settings used for OrthoMCL and state whether any filtering of the input sequences was performed: e.g., to remove multiple splice variants, organellar sequences or very short sequences. Also, change "dicot" to "eudicot"; this needs correcting in several other places as well.

Page 10: For the ten species included in the OrthoMCL analysis, as well as citing the original publications for the genome sequencing, please specify the versions of the genome assemblies and annotations used for each taxon and state where the data were obtained from (e.g. TAIR, Phytozome, etc.).
Page 10, line 54/55: Change "Cruciferous" to "Brassicaceae".
Page 11, line 7/8: Change "species-special" to "species-specific".
Page 11, lines 10-16: It cannot be concluded that X. sorbifolium genes "might keep more structural characters of their ancestors" simply because this species appears to have relatively few genes that are specific to itself alone.
Page 11, lines 21-22: Please provide further details of how the phylogenetic analysis was done with PHYML; e.g., were DNA or protein sequences analysed? Which model of sequence evolution was used? How was support assessed? Etc. Also, the tree in Fig. 3c is rooted on Cucumis sativus, whereas the appropriate outgroup for the set of taxa included in the analysis would be Vitis vinifera. However, even if the tree was rerooted on v. vinifera, the topology is incongruent with the results obtained by previous studies (see for example the summary in Figure 1 of THE ANGIOSPERM PHYLOGENY GROUP 2016, Botanical Journal of the Linnean Society, 181: 1-20). What is the explanation for this? One possibility is that not all gene families (i.e. OrthoMCL clusters) analysed are comprised of solely orthologous sequences; just because the gene clusters/families are single copy doesn't mean all of their members are orthologous, and inclusion of paralogous sequences could confound phylogenetic inference of species relationships. Also, support values (e.g. bootstrap percentages) need to be added to Fig. 3c.
Page 11, line 24: Change "the orthologs" to "the putative orthologs".
Page 11, line 27: Details of how the divergence time estimation was carried out with MCMCtree are lacking. The Authors need to report the parameter settings used, including which molecular clock model was used, and provide details of any fossils used for calibrating the tree. Also, there are no credibility intervals reported for the divergence time estimates in Fig. 3c and the main text; these need to be added.
Page 11, line 29/30: Sentence starting "As two species" needs rewording to improve clarity.
Page 11, line 43/44: Change both instances of "curse" to "curve".
Chromosome synteny between yellowhorn and reference genomes
It might make more sense to move this section to before the section "Identification of gene clusters and duplication" as the results of the synteny analysis are mentioned in that section.
Please specify any parameter settings used for MCscan.
Page 12, line 40/41: Correct "systemic" to "syntenic".
Page 12, line 40/41: Correct "collineartiy" to "collinearity".
The arguments made in this section relating to evidence for conservation of "ancient" chromosomes and support for the "tertiary legacy" status of X. sorbifolium are not convincing to me and I find the text quite confusing and hard to follow. Further clarification is required if this part of the manuscript is to be retained.
Legends
When referring to "mellow fruit", do the Authors mean "ripe fruit"?
Table1 & 2
I suggest replacing "quantity" with "number". E.g. "Number of scaffolds" rather than "Quantity of scaffolds".

Level of Interest

Please indicate how interesting you found the manuscript: Choose an item.

Quality of Written English

Please indicate the quality of language in the manuscript: Choose an item.

Declaration of Competing Interests

Please complete a declaration of competing interests, considering the following questions:

- Have you in the past five years received reimbursements, fees, funding, or salary from an organisation that may in any way gain or lose financially from the publication of this manuscript, either now or in the future?
- Do you hold any stocks or shares in an organisation that may in any way gain or lose financially from the publication of this manuscript, either now or in the future?
- Do you hold or are you currently applying for any patents relating to the content of the manuscript?
- Have you received reimbursements, fees, funding, or salary from an organization that holds or has applied for patents relating to the content of the manuscript?
- Do you have any other financial competing interests?
- Do you have any non-financial competing interests in relation to this paper?

If you can answer no to all of the above, write 'I declare that I have no competing interests' below. If your reply is yes to any, please give details below.

I declare that I have no competing interests

I agree to the open peer review policy of the journal. I understand that my name will be included on my report to the authors and, if the manuscript is accepted for publication, my named report including any attachments I upload will be posted on the website along with the authors' responses. I agree for my report to be made available under an Open Access Creative Commons CC-BY license (http://creativecommons.org/licenses/by/4.0/). I understand that any comments which I do not wish to be included in my named report can be included as confidential comments to the editors, which will not be published.

I agree to the open peer review policy of the journal

To further support our reviewers, we have joined with Publons, where you can gain additional credit to further highlight your hard work (see: https://publons.com/journal/530/gigascience). On publication of

this paper, your review will be automatically added to Publons, you can then choose whether or not to claim your Publons credit. I understand this statement.

Yes Choose an item.
