# Supplementary material for: Pseudomolecule-level assembly of the Chinese oil tree yellowhorn (Xanthoceras sorbifolium) genome
Source: Gigascience. 2019 Jun 26;8(6):giz070. doi: 10.1093/gigascience/giz070 (PMC6593361; doi:10.1093/gigascience/giz070)
Supplement: giz070_Reviewer_2_Report_Revision_1 — Laura Kelly -- 4/18/2019 Reviewed [file giz070_reviewer_2_report_revision_1.pdf]

Reviewer Report

Title: Pseudomolecule-level assembly of the Chinese oil tree yellowhorn (Xanthoceras sorbifolium) genome

Version: Revision 1 **Date:** 4/18/2019

Reviewer name: Laura Kelly

Reviewer Comments to Author:

The Authors appear to have gone to a good deal of effort to address the points I raised previously and I appreciate the work that they have put in. Unfortunately, I still have a concern regarding the k-mer analysis and evidence for heterozygosity in the sequenced individual, which I do not feel has been adequately addressed in the Authors' response.

On page 5, lines 21-25, the Authors say that they use the same formula as Varshney et al., (2012; doi:10.1038/nbt.2022) to calculate estimated genome size from the k-mers: Genome size = k-mer number / peak depth. The Authors determined the peak depth to be 34x. However, an important point is that the k-mer frequency plot in Varshney et al., (their Supplementary Figure 2) has a single clear peak, which they use to define the overall depth. This is not unexpected because Varshney et al., (2012) sequenced an inbred line, and the single clear peak in their k-mer plot reflects the fact that the majority of the genome is homozygous (they estimated 0.067% heterozygosity on the basis of SNP calling against the genome assembly). However, in outbreeding diploid individuals we normally expect to see two peaks - one reflecting k-mers that overlap homozygous positions and another from k-mers that overlap heterozygous positions. The multiplicity (count) of the heterozygous peak should be c. half that of the homozygous peak, as shown in Fig 2 of Kajitani et al (2014; doi/10.1101/gr.170720.113). In the k-mer plot in Fig. 2b of Bi et al., there are two clear peaks, one at c. 35x and the other closer to 70x; this means that a proportion of the 17-mers have a significantly higher depth than that used when calculating the genome size (i.e. 34x), which could lead to an overestimation of the size of the genome based on this method if not accounted for. However, a more important point (given that the Authors also generated genome size estimates by flow cytometry, so do not have to rely on the k-mer based estimate) is the evidence for heterozygosity this indicates. I suggest that the rightmost of the peaks in Fig. 2b (i.e. at c. 70x) is the homogzygous peak and the other is the heterozygous peak, which would indicate a high level of heterozygosity within the sequenced yellowhorn individual. The reason I believe this is as follows - the Authors report that they have 34.40 Gb of cleaned Illumina HiSeq reads (first line on page 5) and that the estimated 1C genome size for yellowhorn is c. 535Mb (average of the two estimates reported on page 4) based on flow cytometry, so the expected whole genome coverage from the Illumina reads is c. x64. Therefore, in a k-mer plot generated from the Illumina reads we would expect to see the homozygous peak at around c. 65x, which is approximately the position of the rightmost peak in Fig. 2b. Consequently, the much higher leftmost peak reflects the heterozygous content of the genome.

As I mentioned in my previous review, the fact that the initial genome assembly (598.65Mb) is significantly larger than expected based on the genome size estimated by flow cytometery is further evidence for heterozygosity. The Authors did not specifically address this point in their response.

Furthermore, it is indicated that c. 95Mb of the assembly is removed during filtering for heterozygous sequences, again suggesting a relatively high level of heterozygosity in the sequenced individual. In the revised version of the manuscript, the Authors estimate % heterozygosity by calling SNPs from reads mapped to the genome assembly, which gives an estimate of 0.30%. This is a much lower level of heterozygosity than would be expected if my assertion regarding the peaks in the k-mer frequency plot is correct. However, there is a lack of detail regarding the parameters used for SNP calling, such as thresholds for mapping quality and any filtering that was applied to the set of raw SNPs. Nevertheless, if we assume that this estimate of heterozygosity is more or less correct, the discrepancy between the expected genome coverage from the Illumina reads and the coverage suggested from the most prominent peak in the k-mer plot based on these reads still needs to be explained. Moreover, it remains the case that the size of the initial genome assembly was significantly larger than the expected genome size based on flow cytometry, and that a substantial amount of sequence was removed during filtering with Purge Haplotigs, which would not be expected if the level of heterozygosity was really only 0.30%.
In addition to the issue discussed above, the English language requires some further improvement. There are a number of small improvements that could be made to increase the clarity and readability of the text. Issues include things like the use of a plural when the singular form should be used (e.g. "individuals" when "individual" is meant) or vice versa (e.g. use of "assemblies" when "assembly" is meant), spelling errors (e.g. "pepline" instead of "pipeline", "eudicot" misspelt as "endicot" in several places) and missing words or incorrect word choice (e.g. "produced via clone" rather than "produced via cloning', "from natural population" rather than "from a natural population"). I recommend that the manuscript be further reviewed for English language prior to publication.

Level of Interest

Please indicate how interesting you found the manuscript: Choose an item.

Quality of Written English

Please indicate the quality of language in the manuscript: Choose an item.

Declaration of Competing Interests

Please complete a declaration of competing interests, considering the following questions:

- Have you in the past five years received reimbursements, fees, funding, or salary from an organisation that may in any way gain or lose financially from the publication of this manuscript, either now or in the future?
- Do you hold any stocks or shares in an organisation that may in any way gain or lose financially from the publication of this manuscript, either now or in the future?
- Do you hold or are you currently applying for any patents relating to the content of the manuscript?

- Have you received reimbursements, fees, funding, or salary from an organization that holds or has applied for patents relating to the content of the manuscript?
- Do you have any other financial competing interests?
- Do you have any non-financial competing interests in relation to this paper?

If you can answer no to all of the above, write 'I declare that I have no competing interests' below. If your reply is yes to any, please give details below.

I declare that I have no competing interests

I agree to the open peer review policy of the journal. I understand that my name will be included on my report to the authors and, if the manuscript is accepted for publication, my named report including any attachments I upload will be posted on the website along with the authors' responses. I agree for my report to be made available under an Open Access Creative Commons CC-BY license (http://creativecommons.org/licenses/by/4.0/). I understand that any comments which I do not wish to be included in my named report can be included as confidential comments to the editors, which will not be published.

I agree to the open peer review policy of the journal

To further support our reviewers, we have joined with Publons, where you can gain additional credit to further highlight your hard work (see: https://publons.com/journal/530/gigascience). On publication of this paper, your review will be automatically added to Publons, you can then choose whether or not to claim your Publons credit. I understand this statement.

Yes Choose an item.
