# Supplementary material for: Pseudomolecule-level assembly of the Chinese oil tree yellowhorn (Xanthoceras sorbifolium) genome
Source: Gigascience. 2019 Jun 26;8(6):giz070. doi: 10.1093/gigascience/giz070 (PMC6593361; doi:10.1093/gigascience/giz070)
Supplement: giz070_Reviewer_3_Report_Original_Submission — Pedro Martinez Garcia -- 11/30/2018 Reviewed [file giz070_reviewer_3_report_original_submission.pdf]

Reviewer Report

Title: Pseudomolecule-level assembly of the Chinese oil tree yellowhorn (Xanthoceras sorbifolium) genome

Version: Original Submission **Date:** 11/30/2018

Reviewer name: Pedro Martinez Garcia

Reviewer Comments to Author:

Thank you for the opportunity to review this Data Note for GigaScience. The MS GICA-D-18-00337 entitled "Reference genome of a Chinese yellowhorn Xanthoceras sorbifolium provides insights into its conservation of original chromosomes" reports on a study aiming to present a high quality genome and to determine the evolutionary history of this species. The presence of whole genome duplication is not detected and the genome structure has received a detailed explanation.
That said, I would like to ask if does the accumulation of LTR-retrotransposons enriched with specific protein coding genes?. Also, I would like to know (if it is possible, I think so) which category is younger? Copia or Gypsy?.
I think a better description about K-mer analysis must be provided, in addition, to cite the paper MarÃ§ais et al. Also some minor changes must be addressed before this MS can be accepted.
Some minor edits
Page 3 Line 33 : change the word Alzheimerand
Page 6 Line 24: please check the N50 number: 1.39Mb or 1.04Mb, please clarify it.
Page 12, Line1: please add a short comment about MCScan's representation, circular shape or circle plot.
Page 12 Line 57, please improve the sentence "as mentioned above analysis of genes …,", something as: "as mentioned before,"

Level of Interest

Please indicate how interesting you found the manuscript: Choose an item.

Quality of Written English

Please indicate the quality of language in the manuscript: Choose an item.

Declaration of Competing Interests

Please complete a declaration of competing interests, considering the following questions:

- Have you in the past five years received reimbursements, fees, funding, or salary from an organisation that may in any way gain or lose financially from the publication of this manuscript, either now or in the future?

- Do you hold any stocks or shares in an organisation that may in any way gain or lose financially from the publication of this manuscript, either now or in the future?
- Do you hold or are you currently applying for any patents relating to the content of the manuscript?
- Have you received reimbursements, fees, funding, or salary from an organization that holds or has applied for patents relating to the content of the manuscript?
- Do you have any other financial competing interests?
- Do you have any non-financial competing interests in relation to this paper?

If you can answer no to all of the above, write 'I declare that I have no competing interests' below. If your reply is yes to any, please give details below.

'I declare that I have no competing interests'

I agree to the open peer review policy of the journal. I understand that my name will be included on my report to the authors and, if the manuscript is accepted for publication, my named report including any attachments I upload will be posted on the website along with the authors' responses. I agree for my report to be made available under an Open Access Creative Commons CC-BY license (http://creativecommons.org/licenses/by/4.0/). I understand that any comments which I do not wish to be included in my named report can be included as confidential comments to the editors, which will not be published.

I agree to the open peer review policy of the journal

To further support our reviewers, we have joined with Publons, where you can gain additional credit to further highlight your hard work (see: https://publons.com/journal/530/gigascience). On publication of this paper, your review will be automatically added to Publons, you can then choose whether or not to claim your Publons credit. I understand this statement.

Yes Choose an item.
